# New Trisulfated Steroids from the Vietnamese Marine Sponge *Halichondria vansoesti* and Their PSA Expression and Glucose Uptake Inhibitory Activities

**DOI:** 10.3390/md17080445

**Published:** 2019-07-27

**Authors:** Kseniya M. Tabakmakher, Tatyana N. Makarieva, Vladimir A. Denisenko, Roman S. Popov, Pavel S. Dmitrenok, Sergey A. Dyshlovoy, Boris B. Grebnev, Carsten Bokemeyer, Gunhild von Amsberg, Nguyen X. Cuong

**Affiliations:** 1 G.B. Elyakov Pacific Institute of Bioorganic Chemistry, Far Eastern Branch of the Russian Academy of Sciences, Pr. 100-let Vladivostoku 159, 690022 Vladivostok, Russia; 2 Department of Oncology, Hematology and Bone Marrow Transplantation with Section Pneumology, Hubertus Wald-Tumorzentrum, University Medical Center Hamburg-Eppendorf, 20251 Hamburg, Germany; 3 Martini-Klinik Prostate Cancer Center, University Hospital Hamburg-Eppendorf, 20251 Hamburg, Germany; 4 Institute of Marine Biochemistry, Vietnam Academy of Science and Technology (VAST), Hanoi 100000, Vietnam

**Keywords:** marine sponge, *Halichondria vansoesti*, trisulfated steroids, topsentiasterol sulfates, halistanol sulfates, anticancer activity, PSA expression, glucose uptake

## Abstract

Seven new unusual polysulfated steroids—topsentiasterol sulfate G (**1**), topsentiasterol sulfate I (**2**), topsentiasterol sulfate H (**3**), bromotopsentiasterol sulfate D (**4**), dichlorotopsentiasterol sulfate D (**8**), bromochlorotopsentiasterol sulfate D (**9**), and 4*β*-hydroxyhalistanol sulfate C (**10**), as well as three previously described—topsentiasterol sulfate D (**7**), chlorotopsentiasterol sulfate D (**5**) and iodotopsentiasterol sulfate D (**6**) have been isolated from the marine sponge *Halichondria vansoesti*. Structures of these compounds were determined by detailed analysis of 1D- and 2D-NMR and HRESIMS data, as well as chemical transformations. The effects of the compounds on human prostate cancer cells PC-3 and 22Rv1 were investigated.

## 1. Introduction

Biologically active trisulfated steroids are characteristic secondary metabolites found in some marine sponges. These polar steroids comprise several structural subgroups in the sponges. The first compound, bearing a common 2*β*,3α,6α-trisulfoxy steroid nucleus, halistanol sulfate, was isolated in 1981 from the Okinawan sponge *Halichondria* cf. *moorei* [1] (Figure S1). The subgroup also includes sokotrasterol sulfate from the sponge *Halichondria* sp. [2], halistanol sulfates A-J and polasterol B, found in the sponges *Epipolasis* sp. [3,4], *Pseudoaxinissa digitata* [5], and *Halichondria* sp. [6], ophirapsranol trisulfate from *Topsentia ophiraphidites* [7], four sterols isolated from the sponges *Trachyopsis halichondroides* and *Cymbastela coralliophila* [8], amaranzoles A-F from *Phorbas amaranthus* [9,10], and topsentinol K trisulfate from the sponge *Topsentia* sp. [11]. Another subgroup of these metabolites consists of ibisterol sulfates and lembesterol A from the sponges *Topsentia* sp. [12], *Xestospongia* sp. [13], and *Petrosia strongilata* [14]. In their steroid nuclei, 2*β*,3α,6α-trisulfoxy functionality is combined with a C-9(11)-double bond and a methyl group at C-14. One more subgroup includes topsentiasterol sulfates A–E from the sponge *Topsentia* sp. [15], Sch 575867 from the deep-water sponge belonging to the family Astroscleridae [16], spheciosterol sulfates from the sponge *Spheciospongia* sp. [17], as well as chloro- and iodotopsentiasterol sulfates D, isolated from the sponge *Topsentia* sp. in our laboratory [18]. These compounds contain a common Δ^9(11)^-unsaturated, 4*β*-hydroxy-14α-methyl, 2*β*,3α,6α-trisulfated steroid nucleus.

In addition to unusual structural features, trisulfated steroids possess promising biological properties [19]. In fact, a broad range of activities has been described to trisulfate steroids such as antibacterial [1,15,20], antifungal [15,16,21], antiviral (including anti-HIV and anti-HSV effects) [5,12,13,22,23], antiparasitic [21], and antiplatelet activities [24]. In addition, the inhibition of different enzymes [6,11,18], promotion of angiogenesis [25], and antitumor activity against various tumor cell lines [7,15,17,26] have been described. Thus, the search for new trisulfated steroids from sponges, including the analyses of their chemical structures and physiological properties, continues to be a promising area of research. Hopefully, this may lead to the development of a new generation of drugs for a broad spectrum of diseases.

**Figure 1:**
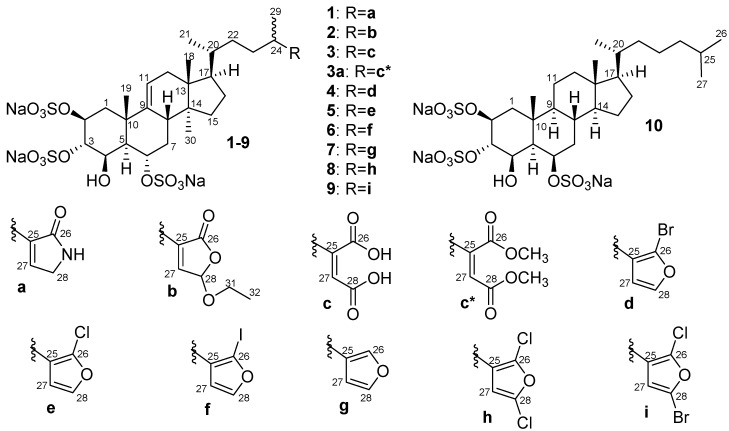
The structures of **1**−**10**.

In the course of our ongoing interest in new biologically active secondary metabolites of marine invertebrates, *Halichondria vansoesti* sponge, collected in Vietnamese waters during the 49th scientific cruise aboard the R/V ‘Academic Oparin’, was investigated. As a result, ten trisulfated steroids **1**–**10** were isolated (Figure 1). Using NMR spectroscopy, including ^1^H, ^13^C, HSQC, COSY, HMBC, and NOESY, as well as high-resolution mass spectrometry and chemical transformations, **1**–**4** and **8**–**10** were identified as new, unusual analogues of topsentiasterol sulfates and halistanol sulfates. Compounds **5**–**7** were previously known as chlorotopsentiasterol sulfate D, iodotopsentiasterol sulfates D [18], and topsentiasterol sulfate D, respectively [15]. Herein, we report the isolation, structural elucidation, proposed biosynthetic pathways, and the study of the biological activities of the isolated compounds.

## 2. Results and Discussion

Concentrated EtOH extract of the sponge *Halichondria vansoesti* was partitioned between aqueous EtOH and *n*-hexane. The aqueous EtOH-soluble materials were further applied on a reversed-phase column chromatography (YMC-gel) and eluted successively with H_2_O→EtOH:H_2_O (3:7)→EtOH:H_2_O (2:3)→EtOH:H_2_O (1:1)→EtOH:H_2_O (3:2) resulting in several subfractions. Subfractions obtained by elution with EtOH:H_2_O (3:7) to EtOH:H_2_O (6:4) were further purified by a reversed-phase HPLC (YMC-ODS-A) to give **1**, **2** and **4**–**10**. The subfraction, eluted with H_2_O, was further extracted with BuOH, after which the butanol solution was concentrated and subjected to a reversed-phase HPLC (YMC-ODS-A) to obtain 3.

The molecular formula of **1**, C_30_H_44_NNa_3_O_14_S_3_, was established from the [M_3Na_ − Na]^−^ ion peak at *m*/*z* 784.1724 in the (−)-HRESIMS. In addition, the peaks at *m*/*z* 380.5927 and 246.0657, corresponding to doubly-, and triply-charged ions ([M_3Na_ − 2Na]^2−^ and [M_3Na_ − 3Na]^3−^), respectively, were indicated in the (−)-HRESIMS of **1** (Figure S2).

The data of 1D- and 2D-NMR spectra of **1** (Table 1 and Table 2, Figures S3–S7) indicated that this compound contained five methyl groups, including three angular methyl groups in the steroid nucleus (δ_H_ 0.70/δ_C_ 15.6, δ_H_ 0.82/δ_C_ 19.4, δ_H_ 1.44/δ_C_ 26.0) and two methyl groups of the side chain (δ_H_ 0.92/δ_C_ 19.5, δ_H_ 1.13/δ_C_ 20.1), eight methylene groups (including a *N*-substituted methylene), eleven methine groups, including four oxygenated methines (δ_H_ 4.98/δ_C_ 76.4, δ_H_ 4.83/δ_C_ 76.6, δ_H_ 4.78/δ_C_ 77.1, δ_H_ 4.49/δ_C_ 69.2), three quaternarysp^3^ carbons (δ_C_ 15.6, δ_C_ 26.0, δ_C_ 19.4), two trisubstituted double bonds (δ_H_ 5.35/δ_C_ 118.2, 147.1, δ_H_ 6.85/δ_C_ 139.4, 144.3), and an amide carbon (δ_C_ 177.6).

**Figure 2:**
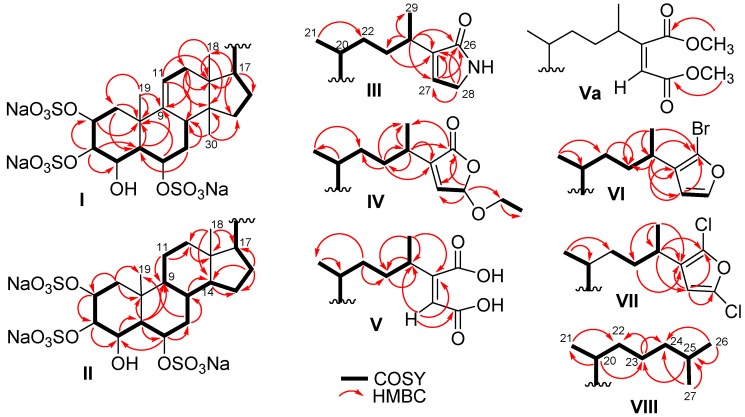
Substructures of **1**–**4** and **8**–**10** with the key COSY (bold line) and HMBC (arrow line) correlations.

**Figure 3:**
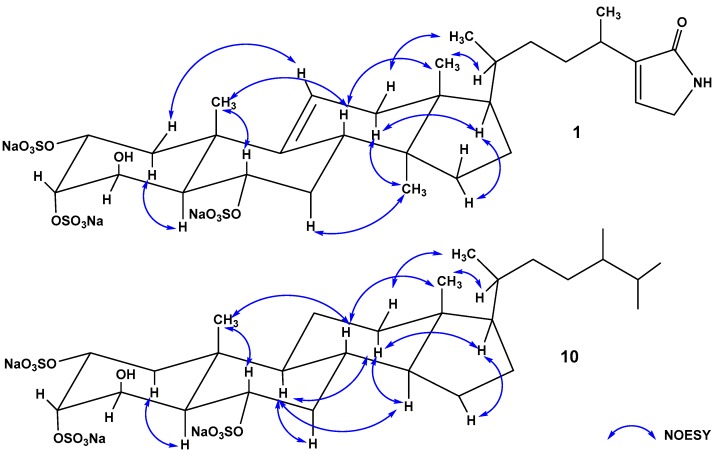
Key NOESY correlations of **1** and **10**.

Further analysis of the 1D- and 2D-NMR data of **1**, and the comparison of its NMR data with those in the literature revealed that **1** contains a Δ^9(11)^-4*β*-hydroxy-14α-methyl-2*β*,3α,6α-trisulfated steroid core (Figure 2, substructure I) and the same side chain, containing C-20 (21) to C-24 (29) (Figure 2, substructure III), as that found in the previously described topsentiasterol sulfates A–E [14], Sch 575867 [16], spheciosterol sulfates A–C [17], and chloro- and iodotopsentiasterol sulfates D (**5**,**6**) [18]. The ^1^H and ^13^C NMR spectra of **1** (Table 1 and Table 2, Figures S3 and S4) were almost identical to those of topsentiasterol sulfate C [15]. The only exceptions were the signals of the protons linked to C-27 and C-28, which were shifted to lower frequencies (δ_H_ 6.85/δ_C_ 139.4, δ_H_ 3.92/δ_C_ 48.3) in the spectrum of **1**. Moreover, the HRESIMS (Figure S2) data showed that the molecular mass of **1** was 1 amu less than that of topsentiasterol sulfate C. Based on the above data, and in combination with 2D NMR data (Figures S5–S7), the presence of the 1,5-dihydro-*2H*-pyrrol-2-one portion in the terminal part of the side chain of **1** (Figure 2, substructure III) was proposed. To the best of our knowledge, this is the first report on the 1,5-dihydro-*2H*-pyrrol-2-one moiety found in polysulfated steroids from sponges. Comparison of the NOESY (Figure S8) data of the steroid nucleus of **1** with those of topsentiasterol sulfate C and the related analogs [15,16,17,18] suggests that all the stereogenic centers of these compounds have the same relative configurations. Key NOESY correlations of the steroid core of **1** are shown in Figure 3. Thus, **1** is a new analogue of the topsentiasterol sulfate C [15], containing a unique structural element with a nitrogen atom in the side chain. Therefore, it was named topsentiasterol sulfate G.

Detailed studies of the 1D- and 2D-NMR spectra of **1**–**4**, **8** and **9** (including the determination of the relative configuration of stereogenic centers using NOESY data, Table 1 and Table 2, Figure 3) were performed. The generated data were compared to those of the known analogs, such as topsentiasterol sulfates A–E [15], Sch 575867 [16], spheciosterol sulfates A–C [17], chlorotopsentiasterol sulfate D (**5**), and iodotopsentiasterol sulfate D (**6**) [18]. Indeed, signals of the steroid nuclei in these compounds and in the isolated polysulfated steroids were almost identical. Therefore, it was proposed that **1**–**4**, **8** and **9** have the same Δ^9(11)^-4*β*-hydroxy-14α-methyl-2*β*,3α,6α-trisulfated steroid nucleus, with a variation of the side chain.

The molecular formula of **2**, C_32_H_47_Na_3_O_16_S_3_, was established from the [M_3Na_ − Na]^−^ ion peak at *m*/*z* 829.1834 in the (−)-HRESIMS. In addition, the peaks at *m*/*z* 403.0980 and 261.0694 in the (−)-HRESIMS of **2** were observed, corresponding to the doubly- and triply-charged ions ([M_3Na_ − 2Na]^2−^ and [M_3Na_ − 3Na]^3−^, respectively) (Figure S9).

The ^1^H and ^13^C NMR data (CD_3_OD, Table 1 and Table 2, Figures S10 and S11) of the side chain of **2** resemble those of topsentiasterol sulfate A [15], except for the presence of the methyl group at δ_H_ 1.24 t, *J* = 7.1/δ_C_ 16.1 (C-32) and a methylene group at δ_H_ 3.74, 3.85/δ_C_ 67.1 (C-31). Further analyses of the 2D-NMR spectral data, including COSY and HMBC spectra (Figures S12 and S14), revealed the following correlations: H-32/H-31, H-31/C-32, H-31/C-28, H-28/C-31 (Figure 2, substructure IV). In addition, HRESIMS spectrum showed that the molecular weight of **2** is 28 amu more than that of topsentiasterol sulfate A [15]. Based on these data, **2** was elucidated as the ethyl ester of topsentiasterol sulfate A [15]. Since **2** has not been previously reported, it was named topsentiasterol sulfate I.

The molecular formula of **3**, C_30_H_43_Na_3_O_17_S_3_, was established from the [M_3Na_ − H]^−^ ion peak at *m*/*z* 839.1253 in the (−)-HRESIMS spectrum. In addition, the peaks at *m*/*z* 408.0685 and 264.3826 in the HRESIMS of **3** corresponding to the doubly- and triply-charged ions ([M_3Na_-Na-H]^2−^ and [M_3Na_-2Na-H]^3−^, respectively) were observed (Figure S16).

The ^13^C and ^1^H NMR of **3** (CD_3_OD+CDCl_3_, ~10:1, Table 1 and Table 2, Figures S17 and S18) exhibited the signals of two carboxyl carbon (δ_C_ 166.8 and 171.9) and a trisubstituted double bond (δ_H_ 5.92/δ_C_ 125.9, 157.9). The HMBC spectrum of **3**, recorded in DMSO-*d*_6_ (Figure S21), displayed correlations from H-29 to C-26 and H-27 to C-28. Based on these data and mass spectrometry data, the presence of 2-substituted maleic acid in the side chain of **3** was suggested (Figure 2, substructure V). The *Z*-configuration of the double bond in this fragment was established using NOESY experiment, in which a correlation from H-29 to H-27 was observed (Figure S22).

**Figure 4:**
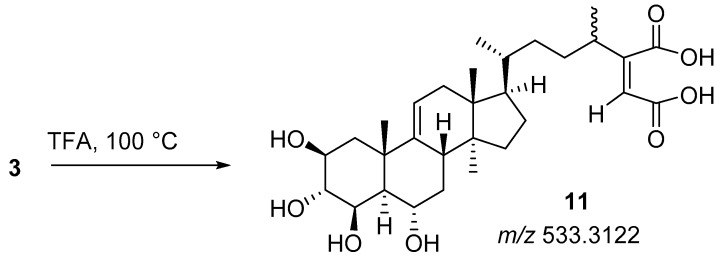
The scheme of the desulfation reaction of **3**.

To confirm the structure of **3**, a methylation with diazomethane was carried out. The structure of the resulting product **3a** was clarified using 2D-NMR and HRESIMS. Cross peaks from OMe-26 to C-26 and OMe-28 to C-28 were observed in the HMBC spectrum (Figure 2, substructure Va). In addition, the peaks of the [M_3Na_ − Na]^−^and [M_3Na_ − 2Na]^2−^ ions were observed in the (−)-HRESIMS at *m*/*z* 845.1764 and 411.0943, respectively (Figure S23). These data revealed that a dimethyl maleate was at the terminal of the side chain of the methylated derivative, as in the previously described topsensterol A, a polyhydroxylated steroid from the sponge *Topsentia sp*. [27]. Additionally, the desulfation reaction of **3** with trifluoroacetic acid was carried out. The structure of the obtained product (**11**) was established from the analysis of the HRESIMS data (Figure 4, Figure S24).

Thus, **3** is a new analogue of polysulfated steroids from sponges, with a 2-substituted maleic acid in the terminal part of the side chain.

Compound **4** was isolated as an inseparable mixture with the previously reported chlorotopsentiasterol sulfate D (**5**) and iodotopsentiasterol sulfate D (**6**) [18] (2:7:1). Detailed analysis of the HRESIMS (Figure S25), 1D- and 2D-NMR spectra (Table 1 and Table 2, Figures S26–S31) of the mixture, as well as the comparison of these data with those for the previously described compounds [15,16,17,18], led to the identification of the structure **4**. The molecular formula of **4** was determined as C_30_H_42_BrNa_3_O_14_S_3_ from the (−)-HRESIMS whose peaks of singly-, doubly-, and triply-charged ions were observed (*m*/*z* 847.0736, [M_3Na_ − Na]^−^, *m*/*z* 412.0424, [M_3Na_ − 2Na]^2−^, *m*/*z* 267.0321, [M_3Na_ − 3Na]^3−^, respectively) (Figure S25). Measured intensities of the isotope peaks of **4** (412.0424 (100.0%), 412.5440 (36.3%), 413.0415 (122.0%), 413.5429 (41.6%), 414.0391 (61.7%) is in a good agreement with the calculation intensities of the isotope peaks for [M_3Na_ − 2Na]^2−^ (412.0414 (100%), 412.5430 (35.8%), 413.0406 (119.8%), 413.5420 (41.3%), 414.0404 (24.0%)). The ^1^H NMR spectrum (CD_3_OD, Table 1, Figure S26) displayed the higher frequency-shifted three pairs of doublets corresponding to H-27 and H-28 of bromo-, chloro-, and iodo- of 3-substituted furans. Comparison of the chemical shift values of these signals to those of chloro-and iodotopsentiasterol sulfates D from the literature [18] allowed us to assign the proton signals at δ_H_ 6.39 (H-27) and 7.48 (H-28) to **4** (Table 1). Using the integration of the signals corresponding to H-27 and H-28 in the ^1^H spectra of the mixture containing **4**, **5**, and **6** showed that the mixture contains about 20% bromotopsentiasterol sulfate D (**4**) and 70% and 10% of chloro- and iodo-derivatives (**5**,**6**) [18], respectively. Additional interpretation of the COSY, HSQC, and HMBC data confirmed that **4** is composed of the substructures I and VI (Figure 2, Figures S28–S30).

New trisulfated steroids, dichlorotopsentiasterol sulfate D (**8**) and bromochlorotopsentiasterol sulfate D (**9**), were isolated as an inseparable mixture. Attempts to separate **8** and **9** using repetitive HPLC failed, however, based on HRESIMS and 1D- and 2D-NMR data, it was estimated as a 9:1 mixture of **8** and **9**. The molecular formulae of the **8** and **9**, C_30_H_41_Cl_2_Na_3_O_14_S_3_ and C_30_H_41_ClBrNa_3_O_14_S_3_, were established from the [M_3Na_ − Na]^−^ ion peaks at *m*/*z* 837.0837 and 881.0340 of the (−)-HRESIMS spectrum. The predominant peaks at *m*/*z* 407.0480 and 429.0227 corresponded to a doubly-charged ions [M_3Na_ − 2Na]^2−^, similar to that in the MS of some pentacyclic guanidine alkaloids [28,29,30,31], and two-headed sphingolipids [32]. Moreover, triply-charged ions [M_3Na_ − 3Na]^3−^ in the spectra of both compounds were also observed (*m*/*z* 263.7026 and 278.3516, respectively) (Figure S32). Intensities of the isotope peaks calculated for **8** confirm the proposed molecular formula C_30_H_41_Cl_2_Na_3_O_14_S_3_ (measured: 407.0480 (100%), 407.5496 (37.2%), 408.0485 (87.6%), 408.5499 (30.8%), 409.0471 (26.6%); calculated for [M_3Na_ − 2Na]^2−^: 407.0472 (100%), 407.5488 (35.8%), 408.0461 (86.5%), 408.5474 (29.36%), 409.0452 (26.7%)). Intensities of the isotope peaks calculated for **9** confirm the proposed molecular formula C_30_H_41_ClBrNa_3_O_14_S_3_ (measured: 429.0227 (100.0%), 429.5244 (35.3%), 430.0219 (149.4%), 430.5233 (52.0%), 431.0213 (61.9%), 431.5225 (19.7%); calculated for [M_3Na_ − 2Na]^2−^: 429.0220 (100.0%), 429.5235 (35.8%), 430.0210 (151.8%), 430.5224 (52.7%), 431.0201 (62.3%), 431.5213 (19.9%)).

The ^1^H and ^13^C NMR spectra of the mixture of **8** and **9** (CD_3_OD, Table 1 and Table 2, Figures S33 and S34) closely resembled those of chlorotopsentiasterol sulfate D (**5**) [18]. The main differences between the NMR spectra of these compounds were the singlet of H-27 at δ_H_ 6.31 for **8** and δ_H_ 6.44 for **9** (integrating these signals, a ratio of **8** to **9** was established as 9:1), instead of two characteristic doublets at δ_H_ 6.39 and 7.36, corresponding to H-27 and H-28 in the ^1^H NMR spectrum of monochlorinated compound **5** [18]. Analysis of the COSY, HSQC, and HMBC spectrum confirmed the substructures I and VII (Figure 2, Figures S35–S37) in **8**.

To determine the positions of the halogen atoms in **9**, we have carried out careful analysis of the ^1^H NMR and COSY spectra of the mixture of **4** and **5** (Table 1, Figures S28 and S37a) and detected two cross-peaks δ_H_ 2.45 (H-24)/δ_H_ 1.14 (H-29) corresponding of **4** (26-bromo) and **5** δ_H_ 2.56 (H-24)/δ_H_ 1.15 (H-29) (26-chloro) in the COSY spectrum. Therefore, in the case of a bulkier bromine substituent at C-26 the chemical shifts of H-24 and H-29 were observed in a higher field. Taking into attention, that the COSY spectra of **8** + **9** (Table 1, Figures S35 and S37a) showed only one cross-peak δ_H_ 2.55 (H-24)/δ_H_ 1.15 (H-29) similar to the cross-peak in the spectrum of **4**, the position of the chlorine atom at C-26 in **9** was established. Based on this data and the HRESIMS data (see above), structure **9** was assigned to the bromochlorotopsentiasterol sulfate D. Nevertheless, the localization of Cl- at C-26 and Br at C-28 in **9** need to be further confirmed.

Compounds **8** and **9** represent the first dihalogenated trisulfated steroids found in sponges.

The molecular formula of **10**, C_27_H_45_Na_3_O_13_S_3_, was established from the [M_3Na_ − Na]^−^ ion peak at *m*/*z* 719.1819 in the (−)-HRESIMS. The base peaks at *m*/*z* 348.0969 corresponded to the doubly-charged ion [M_3Na_ − 2Na]^2−^ (Figure S38).

Detailed analysis of the ^1^H and ^13^C NMR, COSY, HSQC, HMBC, and NOESY spectra of **10** (CD_3_OD, Table 1 and Table 2, Figure 2, substructures II and VIII, Figures S39–S44) and a comparison of its ^1^H and ^13^C chemical shift values with those reported in the literature for the previously described trisulfated steroids [1,2,3,4,5,6,7,8,9,10,11,12,13,14,15,16,17,18], indicated that **10** is a previously unreported 4*β*-hydroxy derivative of halistanol sulfate C [3], which was named 4*β*-hydroxyhalistanol sulfate C.

Interestingly, unlike all the previously described trisulfated steroids containing 4*β*-hydroxy group [15,16,17,18], **10** does not contain a C-9/C-11-double bond and the α-methyl group at C-14. Thus, **10** is the first member of a new structural subgroup of trisulfated steroids from sponges.

**Scheme 1:**
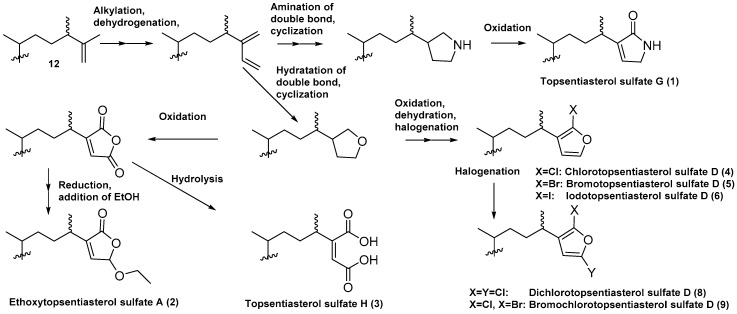
Proposed biogenesis of the side chains in **1**–**9**.

The biosynthesis of unusual side chains of trisulfated steroids, such as **1**–**9**, could be hypothesized to originate from codisterol (**12**) (Figure S45) [33]. This process could proceed via the C-27 alkylation of **12**, followed by the proton loss and several reactions such as amination or hydratation of double bonds accompanied with cyclization, oxidation, hydrolysis, and halogenation, which would result in the formation of **1**–**6**, **8**, and **9** (Scheme 1).

**Figure 5:**
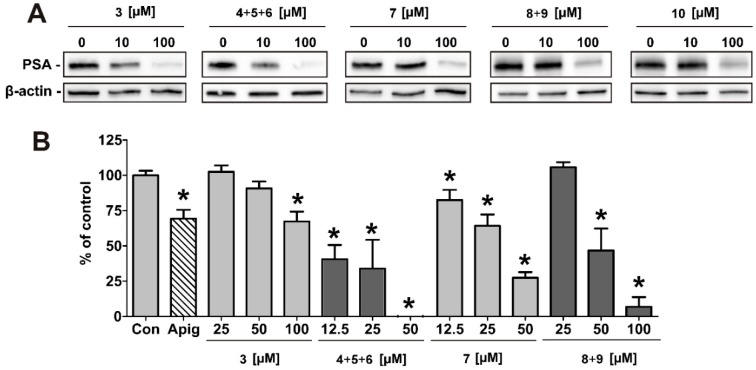
Effects of the compounds on prostate cancer cells. (**A**): Effect on the PSA expression. 22Rv1 cells were treated with the compounds for 24 h, then the proteins were extracted and examined with Western blotting. *β*-Actin was used as a loading control. (**B**): Effect on glucose uptake. PC-3 cells were seeded in the 96-well plate, treated with the test compounds for 24 h in FBS- and glucose-free media, incubated with 2-NBDG, and then the fluorescence was measured. Apigenin (50 µM) was used as a positive control (Apig). Cells treated with vehicle (DMSO) were used as a control (Con). The glucose uptake was normalized to the cell viability, measured by the MTS test. Significant difference from the control is shown as follows: * *p* < 0.05 (Student’s t-test).

The biological activities of **3**, **7**, and **10**, as well as of the mixtures of **4** + **5** + **6** and **8** + **9** were investigated using human prostate cancer cells PC-3 and 22Rv1. PC-3 cells are known to be androgen-independent as they do not express the androgen receptor (AR(-)). 22Rv1 expresses both the androgen receptor (AR(+)), and the androgen receptor splice variant 7 (AR-V7(+)), the expression of AR-V7 mediates the resistant of this cell line to androgen-deprivation therapy [34,35]. PSA is a downstream target gene of the androgen receptor (AR) pathway. Thus, suppression of PSA expression may indicate the inhibition of AR-signaling. AR-signaling is essential for the growth and survival of a significant number of prostate cancer cell types. In fact, downregulation of AR signaling mediated by androgen withdrawal is the standard first-line therapy for advanced human prostate cancer [36]. The isolated compounds and the mixtures were found to inhibit the expression of PSA (prostate-specific antigen) in human drug-resistant 22Rv1 cells (Figure 5A). Compound **3** and the mixture of **4** + **5** + **6**, suppressed PSA expression at a concentration as low as 10 µM (Figure 5A). Note, the IC_50_s for all the isolated compounds determined with the MTT assay in PC-3 and 22Rv1 cells were >100 µM, which could be due to the androgen-independent nature of these particular prostate cancer cell lines.

Additionally, **3** and **7**, as well as the mixtures of **4** + **5** + **6** and **8** + **9** suppressed glucose uptake in 22Rv1 cells (Figure 5B), whereas **10** did not exhibit this effect (data not shown). Cancer cells are characterized by increased glucose consumption, which is related to their rapid growth and metabolism [37]. Inhibition of glucose uptake either by nutrient deprivation or inhibitors, may suppress cancer cells proliferation and/or sensitize cancer cells to standard therapies. Moreover, recent studies suggested a possible crosstalk between glycolysis and AR-signaling [38]. However, cytotoxic effects and proliferation inhibition were observed only at high concentrations of the isolated compounds (data not shown). Nevertheless, due to the promising activity on AR-receptor signaling and glucose uptake, **3** and **7**, as well as the mixtures of **4** + **5** + **6** and **8** + **9** may serve as starting compounds for a development of novel prostate cancer drugs. To the best of our knowledge, this is the very first report on the ability of marine-derived steroid compounds to suppress the PSA expression/androgen receptor signaling, as well as glucose uptake in cancer cells.

## 3. Materials and Methods

### 3.1. General Procedures

Optical rotations were measured using a Perkin-Elmer 343 polarimeter. The ^1^H- and ^13^C NMR spectra were recorded on an Avance III-700 spectrometer at 700 and 175 MHz, respectively. Chemical shifts were referenced to the corresponding residual solvent signal (δ_H_ 3.30/δ_C_ 49.60 in CD_3_OD). The HRESIMS spectra were recorded on a Bruker maXis Impact II mass spectrometer (Bruker, Germany). Low-pressure column liquid chromatography was performed using YMC-GEL ODS-A. HPLC was performed using an Agilent Series 1100 Instrument equipped with a differential refractometer RIDDE14901810 and an YMC-ODS-A (250 × 10 mm) column. Sorbfil Si gel plates (4.5 × 6.0 cm, 5–17 µm, Sorbpolimer, Krasnodar, Russia) were used for thin-layer chromatography. MTT reduction was measured using the F200PRO reader (TECAN, Männedorf, Switzerland).

### 3.2. Animal Material

The sponge *Halichondria vansoesti* (order Suberitida, family Halichondriidae; Figure S46) was collected at a depth of 5–12 m by hand via scuba diving during the 49th scientific cruise on board R/V “Academik Oparin”, in the period from 10 November, 2016 to 3 January, 2017, in the South China Sea (the territorial waters of Vietnam, 12°34′02 N; 109°24′26 E). The sponge material was identified by Grebnev B.B. A voucher specimen is kept under the registration number N 049-232 in the marine invertebrate collection of the G. B. Elyakov Pacific Institute of Bioorganic Chemistry (Vladivostok, Russia).

### 3.3. Extraction and Isolation

The sample of the sponge *Halichondria vansoesti* was immediately frozen after collection and kept at −20 °C. The biological materials (dry weight 13.7 g) were chopped into small pieces and extracted with EtOH (200 mL × 3). The combined EtOH solution was concentrated to obtain the crude ethanol extract (7.9 g), which was partitioned between *n*-hexane and aqueous EtOH (9:4). The aqueous ethanol-soluble materials (6.4 g) was concentrated and further fractionated by CC on YMC-GEL (2.5 × 15 cm) and eluted successively with H_2_O→EtOH:H_2_O (3:7)→EtOH:H_2_O (2:3)→EtOH:H_2_O (1:1)→EtOH:H_2_O (3:2). Each of the subfractions obtained by elution with EtOH:H_2_O (3:7) to EtOH:H_2_O (3:2) were then concentrated (243 mg, 213 mg, 120 mg, 153 mg, respectively) and subjected to repeated preparative HPLC (YMC-ODS-A, 65:35:1 EtOH/H_2_O/1M CH_3_COONH_4_ to give 1 (3.3 mg), 2 (2.3 mg), 7 (15.0 mg), 10 (13.5 mg) and mixtures of 4 + 5 + 6 (12.8 mg) and of 8 + 9 (11.0 mg). The subfraction eluted with H_2_O was further extracted with BuOH, after which the butanol extract was concentrated (691 mg) and subjected to preparative HPLC (YMC-ODS-A, 30:70:1 EtOH/H_2_O/1M CH_3_COONH_4_ to give **3** (4.7 mg).

### 3.4. Compound Characterization Data

Topsentiasterol sulfate G (**1**). Yield 0.024% of the dry weight of the sponge; amorphous powder; ${\left[\alpha \right]}_{D}^{20}$: + 48 (*c* 0.23, MeOH); ^1^H- and ^13^C NMR data, see Table 1 and Table 2; HRESIMS *m*/*z* 784.1724 [M_3Na_ − Na]^−^, 380.5927 [M_3Na_ − 2Na]^2−^, 246.0657 [M_3Na_ − 3Na]^3−^ (calc. for C_30_H_44_NNa_3_O_14_S_3_ 784.1725, 380.5916, 246.0647, respectively).

Topsentiasterol sulfate I (**2**). Yield 0.017% of the dry weight of the sponge; amorphous powder; ${\left[\alpha \right]}_{D}^{20}$: + 69 (*c* 0.15, MeOH); ^1^H- and ^13^C NMR data, see Table 1 and Table 2; HRESIMS *m*/*z* 829.1834 [M_3Na_ − Na]^−^, 403.0980 [M_3Na_ − 2Na]^2−^, 261.0694 [M_3Na_ − 3Na]^3−^ (calc. for C_32_H_47_Na_3_O_16_S_3_, 829.1827, 403.0967, 261.0681, respectively).

Topsentiasterol sulfate H (**3**). Yield 0.034% of the dry weight of the sponge; amorphous powder; ${\left[\alpha \right]}_{D}^{20}$: + 39 (*c* 0.19, EtOH); ^1^H- and ^13^C NMR data, see Table 1 and Table 2; HRESIMS *m*/*z* 839.1253 [M_3Na_ − H]^−^, 408.0685 [M_3Na_ − Na − H]^2−^, 264.3826 [M_3Na_ − 2Na − H]^3−^ (calc. for C_30_H_43_Na_3_O_17_S_3_ 839.1283, 408.0695, 264.3833, respectively).

Bromotopsentiasterol sulfate D, (**4**, in mixture with **5** and **6**). Amorphous powder; ^1^H- and ^13^C-NMR data (of **4**), see Table 1 and Table 2; HRESIMS *m*/*z* 847.0736 [M_3Na_ − Na]^−^, 412.0424 [M_3Na_ − 2Na]^2−^, 267.0321 [M_3Na_ − 3Na]^3−^ (calc. for C_30_H_42_BrNa_3_O_14_S_3_, 847.0721, 412.0414, 267.0312, respectively).

Mixture of the dichlorotopsentiasterol sulfate D and bromochlorotophentiasterol sulfate D (**8** + **9**, 9:1). Amorphous powder; ^1^H- and ^13^C-NMR data (for **8**), see Table 1 and Table 2; HRESIMS (for **8** and **9**) *m*/*z* 837.0837 and 881.0340 [M_3Na_ − Na]^−^, 407.0480 and 429.0227 [M_3Na_ − 2Na]^2−^, 263.7026 and 278.3516 [M_3Na_ − 3Na]^3−^ (calc. for C_30_H_41_Cl_2_Na_3_O_14_S_3_ and C_30_H_41_ClBrNa_3_O_14_S_3_, 837.0836 and 881.0331, 407.0472, and 429.0220, 263.7017 and 278.3513, respectively).

4β-hydroxyhalistanol sulfate C (**10**). Yield 0.099% of the dry weight of the sponge; amorphous powder; ${\left[\alpha \right]}_{D}^{20}$: + 40 (*c* 0.28, MeOH); ^1^H- and ^13^C-NMR data, see Table 1 and Table 2; HRESIMS *m*/*z* 719.1819 [M_3Na_ − Na]^−^, 348.0969 [M_3Na_ − 2Na]^2−^, (calc. for C_27_H_45_Na_3_O_13_S_3_ 719.1823, 348.0965, respectively).

### 3.5. Methylation of ***3***

Compound **3** (0.5 mg) was converted to methyl ester **3a** by treatment with an excess (1.5 mL) of a saturated solution of diazomethane in diethyl ether. The obtained derivative **3a** was analyzed using 2D-NMR and HRESIMS (Figure 2, substructure Va, Figure S23).

### 3.6. Desulfation of ***3***

Compound **3** (<0.1 mg) was dissolved in 500 μL H_2_O and 16 μL of concentrated TFA was added and kept at 100 °C for 4 h. The reaction mixture was concentrated and purified by CC on YMC-GEL (1.5 × 2 cm) and eluted successively with H_2_O→EtOH. The subfraction eluted with EtOH was concentrated to give **11**. The obtained derivative was analyzed using HRESIMS (Figure 4, Figure S24).

### 3.7. Bioactivity Assay

#### 3.7.1. Reagents

The MTT reagent (Thiazolyl blue tetrazolium bromide) was purchased from Sigma (Taufkirchen, Germany). The MTS reagent (Cell Titer 96 Aqueous One Solution Reagent) was purchased from Promega (Madison, WI, USA).

#### 3.7.2. Cell Lines and Culture Conditions

22Rv1 and PC-3 cell lines (human prostate cancer cell lines) were purchased from ATCC. Cells were cultured in monolayer in 10% FBS/RPMI media according to the manufacture’s protocols and were regularly checked for mycoplasma contamination.

#### 3.7.3. In Vitro MTT- and MTS-Based Drug Sensitivity Assay

The in vitro cytotoxic activity of the isolated compounds was evaluated by the MTT assay (performed as described previously [39]). For glucose uptake assay (3.7.5. Glucose uptake assay, see below) the viability was determined using the MTS assay (performed as described previously [40]). Treatment time was 48 h.

#### 3.7.4. Western Blotting

Preparation of the samples and Western blotting were performed as described previously [41]. For the detection of PSA and *β*-actin expression, the anti-PSA/KLK3 (Cell Signaling, #5365, 1:1000) and anti-*β*-actin-HRP (Santa Cruz, sc-1616, 1:10,000) antibodies were used. Treatment time was 24 h.

#### 3.7.5. Glucose Uptake Assay

The examination of the effect of the compounds on glucose uptake was carried out using PC-3 cells and the Glucose Uptake Cell-Based Assay Kit (Cayman Chemicals, Ann Arbor, MI, USA) and normalized to the cell viability measured using the MTS test [40]. 12,000 cells per well were seeded in two 96-well plates in 100 µL of media per well, incubated overnight, and treated with the drugs in 100 µL of FBS-free and glucose-free RPMI media per well for 24 h. For glucose uptake measurements, 10 µL of the 2-NBDG solution in FBS-free and glucose-free RPMI media (glucose uptake measurements, final 2-NBDG concentration in the wells was 50 µg/mL) of the vehicle (for cell viability measurements) was added to each well. After 6 h of incubation, the cells were washed twice with PBS (200 µL/well). Next, for the evaluation of glucose uptake, 100 µL of PBS was added to each well. The fluorescence was measured using Infinite F200PRO reader (TECAN, Männedorf, Switzerland). For cell viability measurements, the 100 µL of culture media containing MTS reagent was added to each well, and cell viability was measured using an Infinite F200PRO reader according to the manufacture’s protocol.

#### 3.7.6. Statistical Analysis

All assays were repeated at least three times. Results are expressed as the mean ± standard deviation (SD). Student’s t-test was used to estimate the significance: * *p* < 0.05.

## 4. Conclusions

Ten polysulfated steroids **1**–**10** were isolated from the Vietnamese marine sponge *Halichondria vansoesti*. The structures of seven previously unreported compounds (**1**–**4** and **8**–**10**) were established by 1D- and 2D-NMR spectroscopy, HRESIMS, and chemical transformations. Compounds **1**–**4**, **8**, and **9** are new analogues of topsentiasterol sulfates. The characteristic Δ^9(11)^-4*β*-hydroxy-14α-methyl-2*β*, 3α, 6α-trisulfated steroid nucleus and unusual side chains, not previously described in trisulfated steroids from sponges, were found in the structures of these compounds. Compound **10** is a new analogue of halistanol sulfate, containing a 4*β*-hydroxy-2*β*, 3α, 6α-trisulfated steroid nucleus and this is the first report of this structure in sponge polar steroids. We proposed hypothetical pathways for the biosynthesis of the side chains in new topsentiasterol sulfates. Some of the isolated trisulfated steroids were able to suppress PSA expression and glucose uptake in human prostate cancer cells and thus may serve as starting compounds for the development of novel prostate cancer drugs.

## Figures and Tables

**Table 1:** ^1^H NMR data for **1**–**4**, **8** and **10**.

Position	1 ^a^ (δ_H_, Mult, *J* in Hz)	2 ^a^ (δ_H_, Mult, *J* in Hz)	3 ^b^ (δ_H_, Mult, *J* in Hz)	4 ^c^ (δ_H_, Mult, *J* in Hz)	8 ^c^ (δ_H_, Mult, *J* in Hz)	10 ^c^ (δ_H_, Mult, *J* in Hz)
1a	1.84 dd (3.6, 14.5)	1.84 brd (14.5)	1.87 dd (3.6, 14.6)	1.83 dd (3.7, 14.5)	1.84 brd (14.8)	1.46 dd (3.6, 14.5)
1b	2.39 brd (14.5)	2.41 brd (14.5)	2.37 brd (14.6)	2.40 brd (14.5)	2.38 brd (14.8)	2.29 brd (14.5)
2	4.98 m	4.98 m	4.96 m	4.97 m	4.98 m	4.87 m
3	4.78 m	4.77 m	4.77 m	4.76 m	4.80 m	4.74 m
4	4.49 m	4.49 m	4.48 m	4.49 m	4.48 m	4.45 m
5	1.51 dd (2.5, 11.4)	1.51 dd (2.7, 11.4)	1.52 dd (2.6, 11.4)	1.51 dd (2.9, 11.3)	1.50 dd (2.5, 11.5)	1.48 m
6	4.83 dt (4.5, 11.4)	4.83 dt (4.5, 11.4)	4.83 dt (4.4, 11.4)	4.83 dt (4.4, 11.3)	4.83 dt (4.5, 11.5)	4.60 dt (4.5, 11.3)
7a	1.58 q (11.9)	1.58 q (11.9)	1.57 dt (11.4, 12.9)	1.57 m	1.57 m	1.11 m
7b	2.23 dt (4.6, 11.9)	2.23 dt (4.6, 11.9)	2.22 dt (4.4, 11.8)	2.21 dt (5.0, 12.0)	2.23 dt (4.5, 11.5)	2.32 dt (4.2, 12.1)
8	2.51 m	2.51 m	2.48 m	2.49 m	2.50 m	1.58 m
9						0.71 m
11a	5.35 brd (5.6)	5.35	5.33 brd (5.8)	5.35 dt (2.0, 6.2)	5.35 dt (2.0, 6.2)	1.50 m
11b						1.34 m
12a	2.13 brd (17.6)	2.13 brd (17.3)	2.10 brd (17.1)	2.11 brd (17.5)	2.12 brd (17.5)	1.14 m
12b	1,97 dd (5.5, 17.6)	1.97 dd (5.9, 17.3)	1.95 dd (5.9, 17.1)	1.95 ddd (1.5, 6.1, 17.5)	1.95 dd (6.0, 17.5)	2.01 dt (12.5, 3.4)
14						1.11 m
15a	1.39 m	1.39 m	1.30 m	1.37 m	1.38 m	1.62 m
15b	1.47 m	1.46 m	1.41 m	1.45 m	1.46 m	1.12 m
16a	1.33 m	1.33 m	1.30 m	1.25 m	1.27 m	1.28 m
16b	1.91 m	1.91 m	1.92 m	1.86 m	1.87 m	1.85 m
17	1.66 q (9.4)	1.66 q (9.4)	1.64 q (9.3)	1.63 m	1.63 m	1.13 m
18	0.70 s	0.71 s	0.68 s	0.68 s	0.69 s	0.69 s
19	1.44 s	1.45 s	1.42 s	1.44 s	1.45 s	1.29 s
20	1.42 m	1.43 m	1.38	1.37 m	1.45 s	1.38 m
21	0.92 d (6.5)	0.92 d (6.5)	0.90 d (6.5)	0.90 d (6.5)	0.90 d (6.5)	0.93 d (6.6)
22a	1.09 m	1.09 m	1.08 m	1.04 m	1.05 m	1.01 m
22b	1.49 m	1.43 m	1.40 m	1.45 m	1.46 m	
23a	1.51 m	1.52 m	1.42 m	1.38 m	1.36 m	1.18 m
23b	1.55 m	1.55 m	1.46 m	1.62 m	1.61 m	
24a	2.48 m	2.48 m	2.69 m	2.45 m	2.55 m	1.11 m
24b						1.12 m
25						1.53 m
26						0.87 d (6.6)
27	6.85 br s	6.93 br s	5.92 br s	6.39 d (2.0)	6.31 s	0.87 d (6.6)
28	3.92 br s	5.91 br s		7.48 d (2.0)		
29	1.13 d (6.9)	1.15 d (6.9)	1.10 d (7.0)	1.14 d (7.0)	1.15 d (6.9)	
30	0.82 s	0.82 s	0.82 s	0.81 s	0.81 s	
31a		3.74 m				
31b		3.85 m				
32		1.24 t (7.1)				

^a^ Measured in CD_3_OD at 500 MHz. ^b^ Measured in a mixture of CD_3_OD + CDCl_3_ (~10:1) at 700 MHz. ^c^ Measured in CD_3_OD at 700 MHz.

**Table 2:** ^13^C NMR Data of **1**–**4**, **8**, and **10**.

Position	1 ^a^ (δ_C_, Type)	2 ^a^ (δ_C_, Type)	3 ^b^ (δ_C_, Type)	4 ^a^ (δ_C_, Type)	8 ^a^ (δ_C_, Type)	10 ^a^ (δ_C_, Type)
1	37.9, CH_2_	37.8, CH_2_	37.7, CH_2_	37.8, CH_2_	38.0, CH_2_	39.3, CH_2_
2	76.4, CH	76.4, CH	76.3, CH	76.4, CH	76.4, CH	76.3, CH
3	77.1, CH	77.2, CH	76.8, CH	77.1, CH	77.2, CH	77.3, CH
4	69.2, CH	69.2, CH	69.0, CH	69.2, CH	69.2, CH	69.0, CH
5	48.7, CH	48.6, CH	48.3, CH	48.6, CH	48.7, CH	50.8, CH
6	76.6, CH	76.4, CH	76.5, CH	76.6, CH	76.6, CH	76.4, CH
7	36.1, CH_2_	36.1, CH_2_	35.8, CH_2_	36.1, CH_2_	36.1, CH_2_	40.8, CH_2_
8	42.0, CH	42.0, CH	41.7, CH	42.0, CH	42.0, CH	35.9, CH
9	147.1, C	147.2, C	147.1, C	147.1, C	147.2, C	57.1, CH
10	40.1, C	40.2, C	40.1, C	40.2, C	40.1, C	37.6, C
11	118.2, CH	118.2, CH	118.2, CH	118.2, CH	118.2, CH	22.0, CH_2_
12	38.9, CH_2_	38.9, CH_2_	38.7, CH_2_	38.9, CH_2_	38.9, CH_2_	41.7, CH_2_
13	46.2, C	46.2, C	46.2, C	46.2, C	46.2, C	44.4, C
14	48.7, C	48.7, C	48.7, C	48.7, C	48.7, C	58.2, CH
15	35.5, CH_2_	35.5, CH_2_	35.3, CH_2_	35.5, CH_2_	35.4, CH_2_	25.8, CH_2_
16	29.5, CH_2_	29.5, CH_2_	29.3, CH_2_	29.4, CH_2_	29.5, CH_2_	29.8, CH_2_
17	52.8, CH	52.8, CH	52.7, CH	52.8, CH	52.8, CH	58.2, CH
18	15.6, CH_3_	15.7, CH_3_	15.6, CH_3_	15.6, CH_3_	15.6, CH_3_	13.1, CH_3_
19	26.0, CH_3_	26.0, CH_3_	25.8, CH_3_	26.0, CH_3_	26.0, CH_3_	18.0, CH_3_
20	37.9, CH	37.8, CH	37.7, CH	38.0, CH	38.0, CH	37.7, CH
21	19.5, CH_3_	19.4, CH_3_	19.4, CH_3_	19.6, CH_3_	19.5, CH_3_	19.8, CH_3_
22	35.3, CH_2_	35.2, CH_2_	35.4, CH_2_	35.8, CH_2_	35.7, CH_2_	37.9, CH_2_
23	33.8, CH_2_	33.4, CH_2_	34.2, CH_2_	35.4, CH_2_	35.4, CH_2_	25.5, CH_2_
24	32.4, CH	32.5, CH	39.7, CH	32.2, CH	32.5, CH	41.3, CH_2_
25	144.3, C	144.6, C	157.9, C	133.6, C	129.9, C	29.7, CH
26	177.6, C	173.7, C	171.9, C	126.4, C	132.1, C	23.5, CH_3_
27	139.4, CH	144.4, CH	125.9, CH	112.4, CH	109.6, CH	23.8, CH_3_
28	48.3, CH_2_	104.0, CH	166.8, C	146.0, CH	136.1, C	
29	20.1, CH_3_	19.5, CH_3_	20.5, CH_3_	21.7, CH_3_	21.4, CH_3_	
30	19.4, CH_3_	19.4, CH_3_	19.4, CH_3_	19.4, CH_3_	19.4, CH_3_	
31		67.1, CH_2_				
32		16.1, CH_3_				

^a^ Measured in CD_3_OD at 175 MHz. ^b^ Measured in a mixture of CD_3_OD + CDCl_3_ (~10:1) at 175 MHz.

## Supplementary Materials

The following are available online at https://www.mdpi.com/1660-3397/17/8/445/s1, Figure S1: List of the previously described polysulfated steroids, combined into subgroups in accordance with the structural features of the steroid nucleus, Figures S2–S44: Copies of HRESIMS, 1D- and 2D-NMR spectra of **1**–**10**, Figure S45: Structure of codisterol (**12**), Figure S46: Photo of the studied sample of sponge *Halichondria vansoesti* (registration number N 049-232).
