# Supplementary material for: New Trisulfated Steroids from the Vietnamese Marine Sponge Halichondria vansoesti and Their PSA Expression and Glucose Uptake Inhibitory Activities
Source: Mar Drugs. 2019 Jul 27;17(8):445. doi: 10.3390/md17080445 (PMC6723502; doi:10.3390/md17080445)

Supplementary Materials

New Trisulfated Steroids from the Vietnamese Marine Sponge *Halichondria vansoesti* and their PSA Expression and Glucose Uptake Inhibitory Activities

Kseniya M. Tabakmakher[1], Tatyana N. Makarieva[1*], Vladimir A. Denisenko[1], Roman S. Popov[1], Pavel S. Dmitrenok[1], Sergey A. Dyshlovoy[1,2], Boris B. Grebnev[1], Carsten Bokemeyer[2], Gunhild von Amsberg[2], and Nguyen X. Cuong[3]

[1] G.B. Elyakov Pacific Institute of Bioorganic Chemistry, Far Eastern Branch of the Russian Academy of Sciences, Pr. 100-let Vladivostoku 159, 690022 Vladivostok, Russia
tabakmakher_km@piboc.dvo.ru (K.M.T.); dyshlovoy@gmail.com (S.A.D.); vladenis@piboc.dvo.ru (V.A.D.); prs_90@mail.ru (R.S.P.); grebnev_bor@mail.ru (G.B.B.)

[2] Department of Oncology, Hematology and Bone Marrow Transplantation with Section Pneumology, Hubertus Wald-Tumorzentrum, University Medical Center Hamburg-Eppendorf, Hamburg, Germany; c.bokemeyer@uke.de (B.C.); g.von-amsberg@uke.de (v.A.G.)

[3] Institute of Marine Biochemistry, Vietnam Academy of Science and Technology (VAST), Hanoi, Vietnam; cuongnx@imbc.vast.vn (N.X.C.)

* Correspondence: makarieva@piboc.dvo.ru (T.N.M.) Tel.: +7-950-295-66-25

List

Figure S1. List of the previously described polysulfated steroids, combined into subgroups in accordance with the structural features of the steroid nucleus.
Figure S2. HRESIMS of topsentiasterol sulfate G (**1**).
Figure S3. ^{1}H-NMR spectrum of topsentiasterol sulfate G (**1**) in CD_3OD.
Figure S4. ^{13}C-NMR spectrum of topsentiasterol sulfate G (**1**) in CD_3OD.
Figure S5. ^{1}H-^{1}H-COSY spectrum of topsentiasterol sulfate G (**1**) in CD_3OD.
Figure S6. HSQC spectrum of topsentiasterol sulfate G (**1**) in CD_3OD.
Figure S7. HMBC spectrum of topsentiasterol sulfate G (**1**) in CD_3OD.
Figure S8. NOESY spectrum of topsentiasterol sulfate G (**1**) in CD_3OD.
Figure S9. HRESIMS of topsentiasterol sulfate I (**2**).
Figure S10. ^{1}H-NMR spectrum of topsentiasterol sulfate I (**2**) in CD_3OD.
Figure S11. ^{13}C-NMR spectrum of topsentiasterol sulfate I (**2**) in CD_3OD.
Figure S12. ^{1}H-^{1}H-COSY spectrum of topsentiasterol sulfate I (**2**) in CD_3OD.
Figure S13. HSQC spectrum of topsentiasterol sulfate I (**2**) in CD_3OD.
Figure S14. HMBC spectrum of topsentiasterol sulfate I (**2**) in CD_3OD.
Figure S15. NOESY spectrum of topsentiasterol sulfate I (**2**) in CD_3OD.
Figure S16. HRESIMS of topsentiasterol sulfate H (**3**).
Figure S17. ^{1}H-NMR spectrum of topsentiasterol sulfate H (**3**) in CD_3OD+$CDCl_3$ (~10:1).
Figure S18. ^{13}C-NMR spectrum of topsentiasterol sulfate H (**3**) in CD_3OD+$CDCl_3$ (~10:1).
Figure S19. ^{1}H-^{1}H-COSY spectrum of topsentiasterol sulfate H (**3**) in CD_3OD+$CDCl_3$ (~10:1).
Figure S20. HSQC spectrum of topsentiasterol sulfate H (**3**) in CD_3OD+$CDCl_3$ (~10:1).
Figure S21. HMBC spectrum of topsentiasterol sulfate H (**3**) in DMSO-d_6.
Figure S22. NOESY spectrum of topsentiasterol sulfate H (**3**) in CD_3OD+$CDCl_3$ (~10:1).
Figure S23. HRESIMS of the topsentiasterol sulfate H methylation reaction product.
Figure S24. HRESIMS of the topsentiasterol sulfate H desulfation reaction product (**11**).
Figure S25. HRESIMS of bromotopsentiasterol sulfate D (**4**) (in mixture with **5** and **6**).
Figure S26. ^{1}H-NMR spectrum of bromotopsentiasterol sulfate D (**4**) (in mixture with **5** and **6**) in CD_3OD.
Figure S27. ^{13}C-NMR spectrum of bromotopsentiasterol sulfate D (**4**) (in mixture with **5** and **6**) in CD_3OD.
Figure S28. ^{1}H-^{1}H-COSY spectrum of bromotopsentiasterol sulfate D (**4**) (in mixture with **5** and **6**) in CD_3OD.
Figure S29. HSQC spectrum of bromotopsentiasterol sulfate D (**4**) (in mixture with **5** and **6**) in CD_3OD.
Figure S30. HMBC spectrum of bromotopsentiasterol sulfate D (**4**) (in mixture with **5** and **6**) in CD_3OD.
Figure S31. NOESY spectrum of bromotopsentiasterol sulfate D (**4**) (in mixture with **5** and **6**) in CD_3OD.
Figure S32. HRESIMS of dichlorotopsentiasterol sulfate D (**8**) (in mixture with **9**) in CD_3OD.
Figure S33. ^{1}H-NMR spectrum of dichlorotopsentiasterol sulfate D (**8**) (in mixture with **9**) in CD_3OD.
Figure S34. ^{13}C-NMR spectrum of dichlorotopsentiasterol sulfate D (**8**) (in mixture with **9**) in CD_3OD.
Figure S35. ^{1}H-^{1}H-COSY spectrum of dichlorotopsentiasterol sulfate D (**8**) (in mixture with **9**) in CD_3OD.
Figure S36. HSQC spectrum of dichlorotopsentiasterol sulfate D (**8**) (in mixture with **9**) in CD_3OD.
Figure S37. HMBC spectrum of dichlorotopsentiasterol sulfate D (**8**) (in mixture with **9**) in CD_3OD.
Figure S37a. Fragments of COSY spectra: **A**: for bromotopsentiasterol sulfate D (**4**) (in mixture with **5** and **6**) in CD_3OD; **B**: for a mixture of dichlorotopsentiasterol sulfate D and bromochlorotopsentiasterol sulfate D (**8**+**9**) in CD_3OD.
Figure S38. HRESIMS of 4β-hydroxy-halistanol sulfate C (**10**).
Figure S39. ^{1}H-NMR spectrum of 4β-hydroxyhalistanol sulfate C (**10**) in CD_3OD.
Figure S40. ^{13}C-NMR spectrum of 4β-hydroxyhalistanol sulfate C (**10**) in CD_3OD.
Figure S41. ^{1}H-^{1}H-COSY spectrum of 4β-hydroxyhalistanol sulfate C (**10**) in CD_3OD.
Figure S42. HSQC spectrum of 4β-hydroxyhalistanol sulfate C (**10**) in CD_3OD.
Figure S43. HMBC spectrum of 4β-hydroxyhalistanol sulfate C (**10**) in CD_3OD.
Figure S44. NOESY spectrum of 4β-hydroxyhalistanol sulfate C (**10**) in CD_3OD.
Figure S45. Structure of codisterol (**12**).
Figure S46. Photo of the studied sample of sponge *Halichondria vansoesti* (registration number № 049-232).

Figure S1. List of the previously described polysulfated steroids, combined into subgroups in accordance with the structural features of the steroid nucleus.

Subgroup of steroids with a 2β,3α,6α-trisulfoxy steroid nucleus:

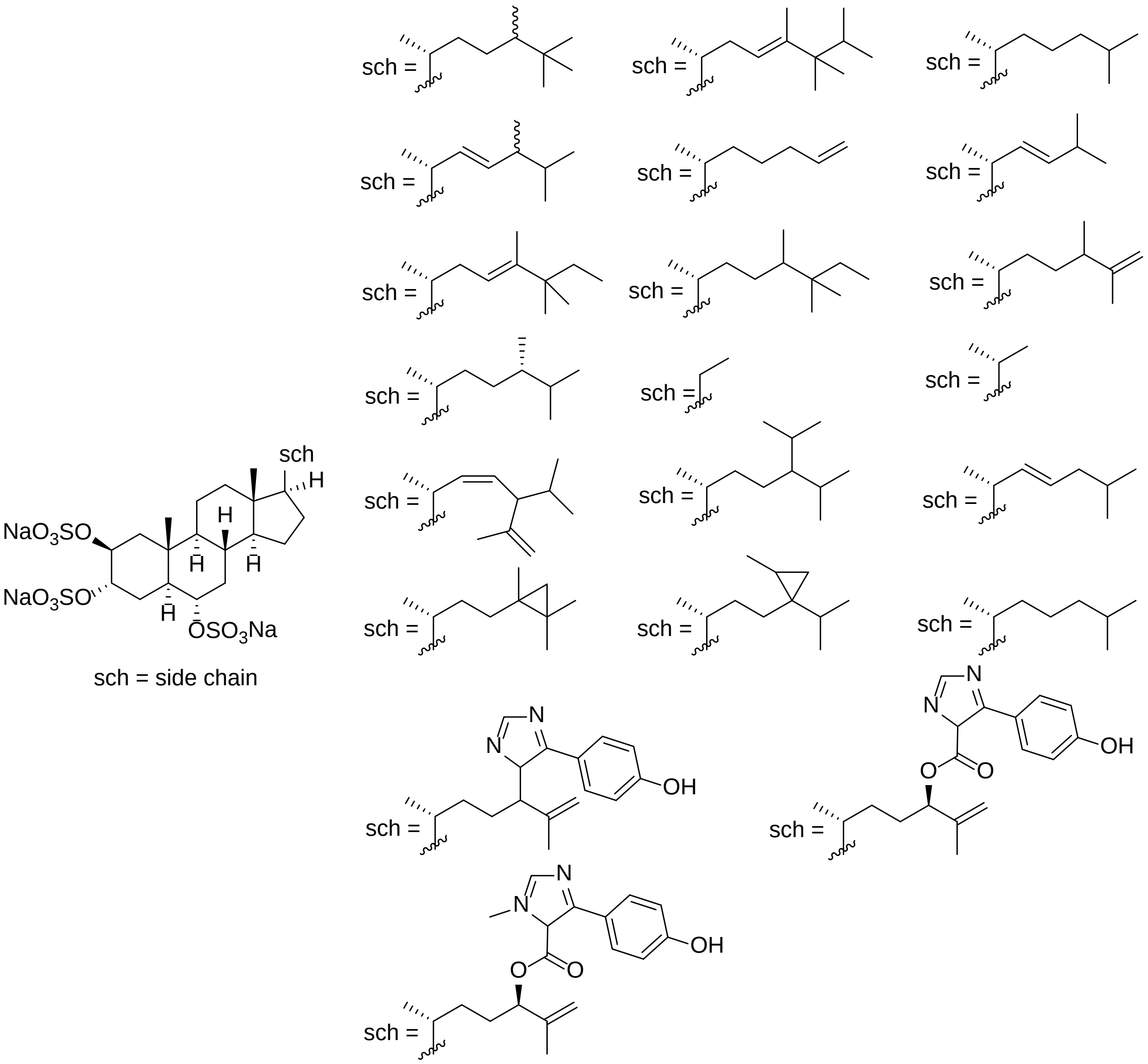

References:

1. Fusetani, N.; Matsunaga, S.; Konosu, S. Bioactive marine metabolites II. Halistanol sulfate, an antimicrobial novel steroid sulfate from the marine sponge *Halichondria* cf. *moorei* Bergquist. *Tetrahedron Lett.* **1981**, *22*, 1985–1988, doi: 10.1016/S0040-4039(01)92885-0.
2. Makarieva, T.N.; Shubina, L.K.; Kalinovsky, A.I.; Stonik, V.A; Elyakov, G.B. Steroids in porifera. II. Steroid derivatives from two sponges of the family *Halichondriidae*. Sokotrasterol sulfate, a marine steroid with a new pattern of side chain alkylation. *Steroids*. **1983**, *42*, 267-281, doi: 10.1016/0039-128X(83)90039-9.
3. Kanazawa, S.; Fusetani, N.; Matsunaga, S. Halistanol sulfates A-E, new steroid sulfates, from a marine sponge, *Epipolasis* sp. *Tetrahedron*. **1992**, *48*, 5467-5472, doi: 10.1016/S0040-4020(01)88300-9.
4. Umeyama, A.; Adachi, K.; Ito, S.; Arihara S. New 24-Isopropylcholesterol and 24-Isopropenylcholesterol sulfate from the marine sponge *Epipolasis* Species. *J. Nat. Prod.* **2000,** *63*, 1175-1177, doi: 10.1021/np000024j.
5. Bifulco, G.; Bruno, I.; Minale, L.; Riccio, R. Novel HIV-inhibitory halistanol sulfates F-H from a marine sponge, *Pseudoaxinissa digitata*. *J. Nat. Prod.* **1994**, *57*, 164-167, doi: 10.1021/np50103a026.

6. Nakamura, F.; Kudo, N.; Tomachi, Y.; Nakata, A.; Takemoto, M.; Ito, A.; Tabei, H.; Arai, D.; de Voogd, N.; Yoshida, M.; Yoichi, N.; Nobuhiro, F. Halistanol sulfates I and J, new SIRT1–3 inhibitory steroid sulfates from a marine sponge of the genus *Halichondria*. *J. Antibiot.* **2018**, *71*, 273–278, doi: 10.1038/ja.2017.145.
7. Gunasekera, S.P.; Sennett, S.H.; Kelly-Borges, M.; Bryant, R.W. Ophirapstanol trisulfate, a new biologically active steroid sulfate from the deep water marine sponge *Topsentia ophiraphidites*. *J. Nat. Prod.* **1994**, *57*, 1751–1754, doi: 0.1021/np50114a024.
8. Makarieva, T.N.; Stonik, V.A; Dmitrenok, A.S.; Krasokhin, V.B.; Svetashev, V.I.; Vysotskii, M.V. New polar steroids from the sponges *Trachyopsis halichondroides* and *Cymbastela coralliophila*. *Steroids*. **1995**, *60*, 316-320, doi: 10.1016/0039-128X(94)00056-I.
9. Morinaka, B.I.; Masuno, M.N.; Pawlik, J.R.; Molinski, T.F. Amaranzole A, a new *N*-imidazolyl steroid from *Phorbasam aranthus*. *Org. Lett.* **2007**, *9*, 5219-5222, doi: 10.1021/ol702325e.
10. Morinaka, B.I.; Pawlik, J.R.; Molinski, T.F. Amaranzoles B-F, imidazole-2-carboxy steroids from the marine sponge *Phorbasam aranthus*. C24-N- and C24-O-analogues from a divergent oxidative biosynthesis. *J. Org. Chem.* **2010**, *75*, 2453–2460, doi: 10.1021/jo1000324.

Subgroup of steroids with $\Delta^{9(11)}$-unsaturated, 14α-methyl, 2β,3α,6α-trisulfoxy steroid nucleus:

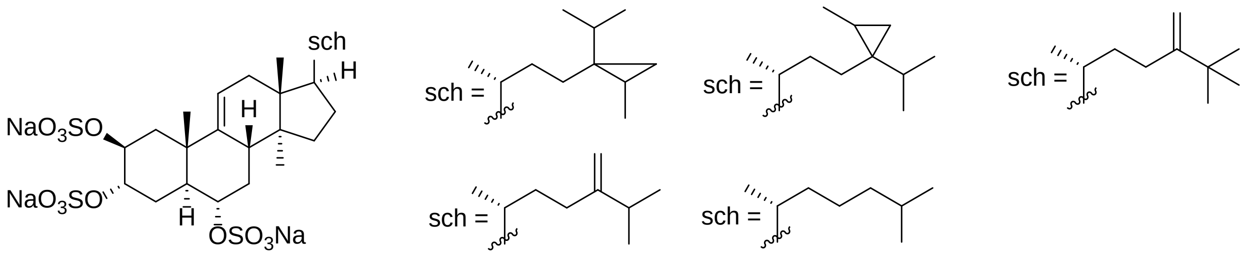

sch = side chain

References:

11. McKee, T.C.; Cardellinaii, J.H.; Tischler, M.; Snader, K.M.; Boyd, M.R. Ibisterol sulfate, a novel HIV-inhibitory sulfated sterol from the deep water sponge *Topsentia* sp. *Tetrahedron Lett.* **1993**, *34*, 389–392, doi: 10.1016/0040-4039(93)85083-9.
12. Lerch, M.L.; Faulkner, D.J. Unusual polyoxygenated sterols from a Philippines sponge *Xestospongia* sp. *Tetrahedron*. **2001**, *57*, 4091–4094, doi: 10.1016/S0040-4020(01)00296-4.
13. Aoki, S.; Naka, Y.; Itoh, T.; Furukawa, T.; Rachmat, R.; Akiyama, S.; Kobayashi, M. Lembehsterols A and B, novel sulfated sterols inhibiting thymidine phosphorylase, from the marine sponge *Petrosia strongylata*. *Chem. Pharm. Bull.* **2002**, *50*, 827–830, doi: 10.1248/cpb.50.827.

Subgroup of steroids with $\Delta^{9(11)}$-unsaturated, 4β-hydroxy-14α-methyl, 2β,3α,6α-trisulfoxy steroid nucleus:

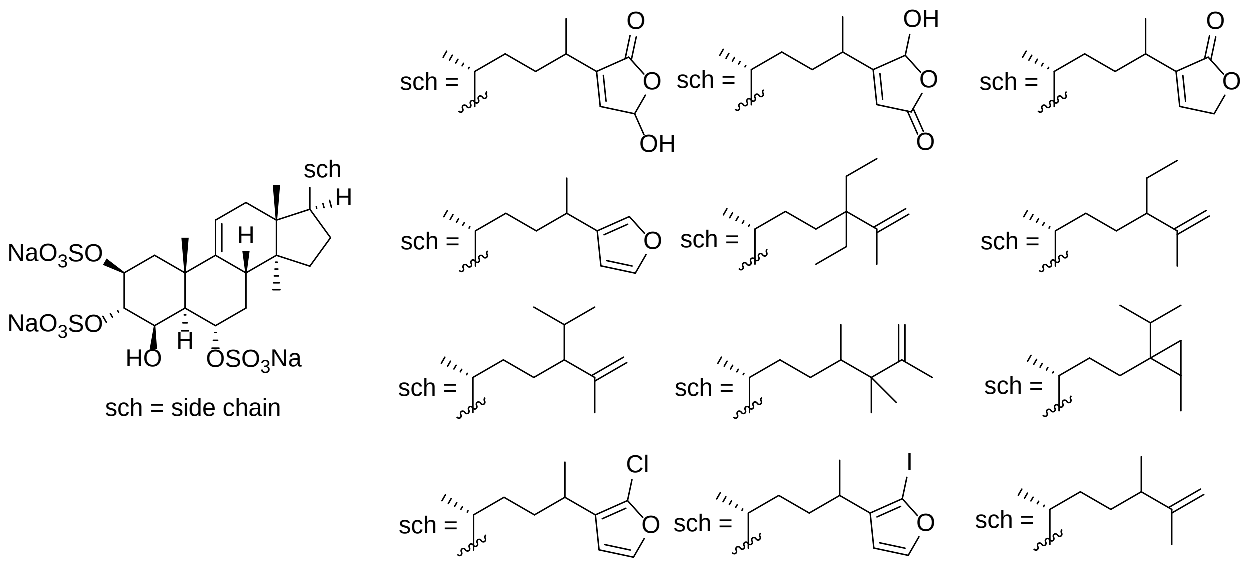

References:

14. Fusetani, N.; Takahashi, M.; Matsunaga, S. Topsentiasterol sulfates, antimicrobial sterol sulfates possessing novel side chains, from a marine sponge, *Topsentia* sp. *Tetrahedron*. **1994**, *50*, 7765–7770, doi: 10.1016/S0040-4020(01)85260-1.
15. Yang, S.W.; Chan, T.M.; Pomponi, S.A.; Chen, G.; Loebenberg, D.; Wright, A.; Patel, M.; Gullo, V.; Pramanik, B.; Chu, M. Structure elucidation of a new antifungal sterol sulfate, Sch 575867, from a deep-water marine sponge (Family: Astroscleridae). *Journal of Antibiotics*. **2003**, *56*, 186–189, doi: 10.7164/antibiotics.56.186.
16. Whitson, E.L.; Bugni, T.S.; Chockalingam, P.S.; Concepcion, G.P.; Harper, M.K.; He, M.; Hooper, J.N.A.; Mangalindan, G.C.; Ritacco, F.; Ireland, C.M. Spheciosterol sulfates, PKCζ inhibitors from a Philippine sponge *Spheciospongia* sp. *J. Nat. Prod*. **2008**, *71*, 1213–1217, doi: 10.1021/np8001628.
17. Guzii, A.G.; Makarieva, T.N.; Denisenko, V.A.; Dmitrenok, P.S.; Burtseva, Y.V.; Krasokhin, V.B.; Stonik, V.A. Topsentiasterol sulfates with novel iodinated and chlorinated side chains from the marine sponge *Topsentia* sp. *Tetrahedron Lett*. **2008**, *49*, 7191–7193, doi: 10.1016/j.tetlet.2008.10.007.

Figure S2. HRESIMS of topsentiasterol sulfate G (**1**).

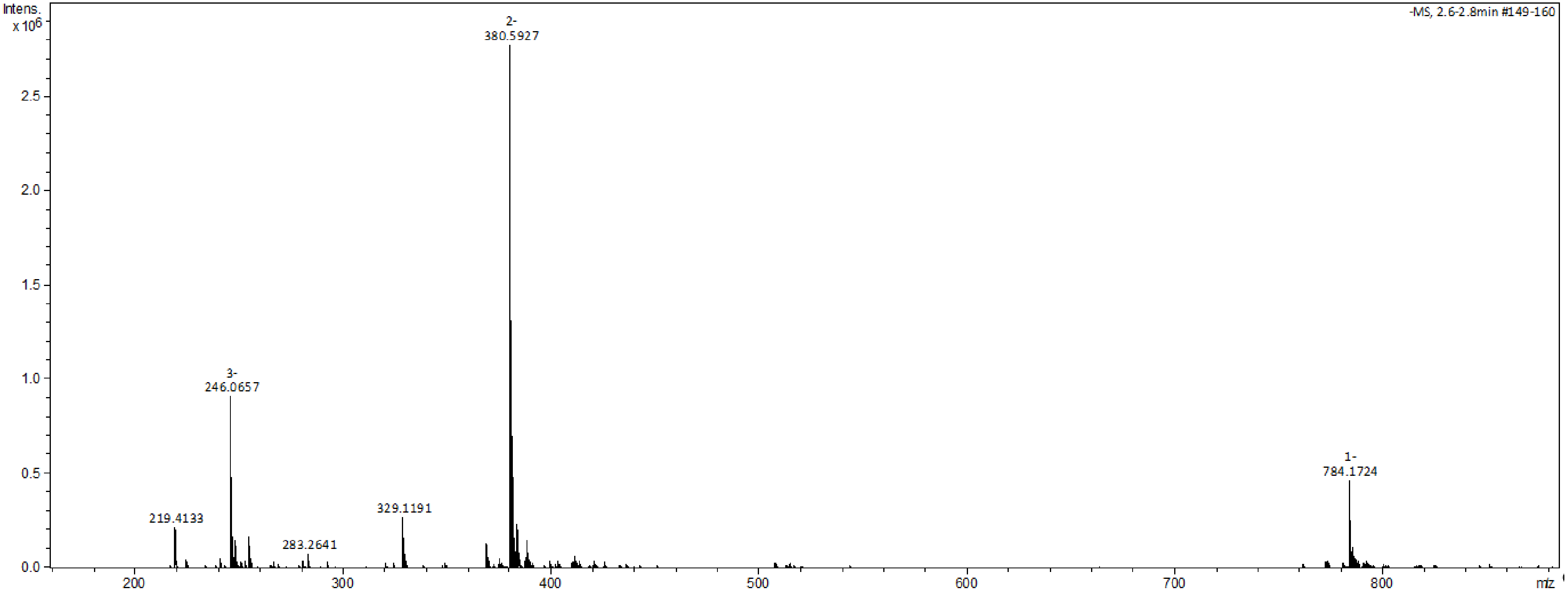

Figure S3. ^{1}H-NMR spectrum of topsentiasterol sulfate G (**1**) in CD_3OD.

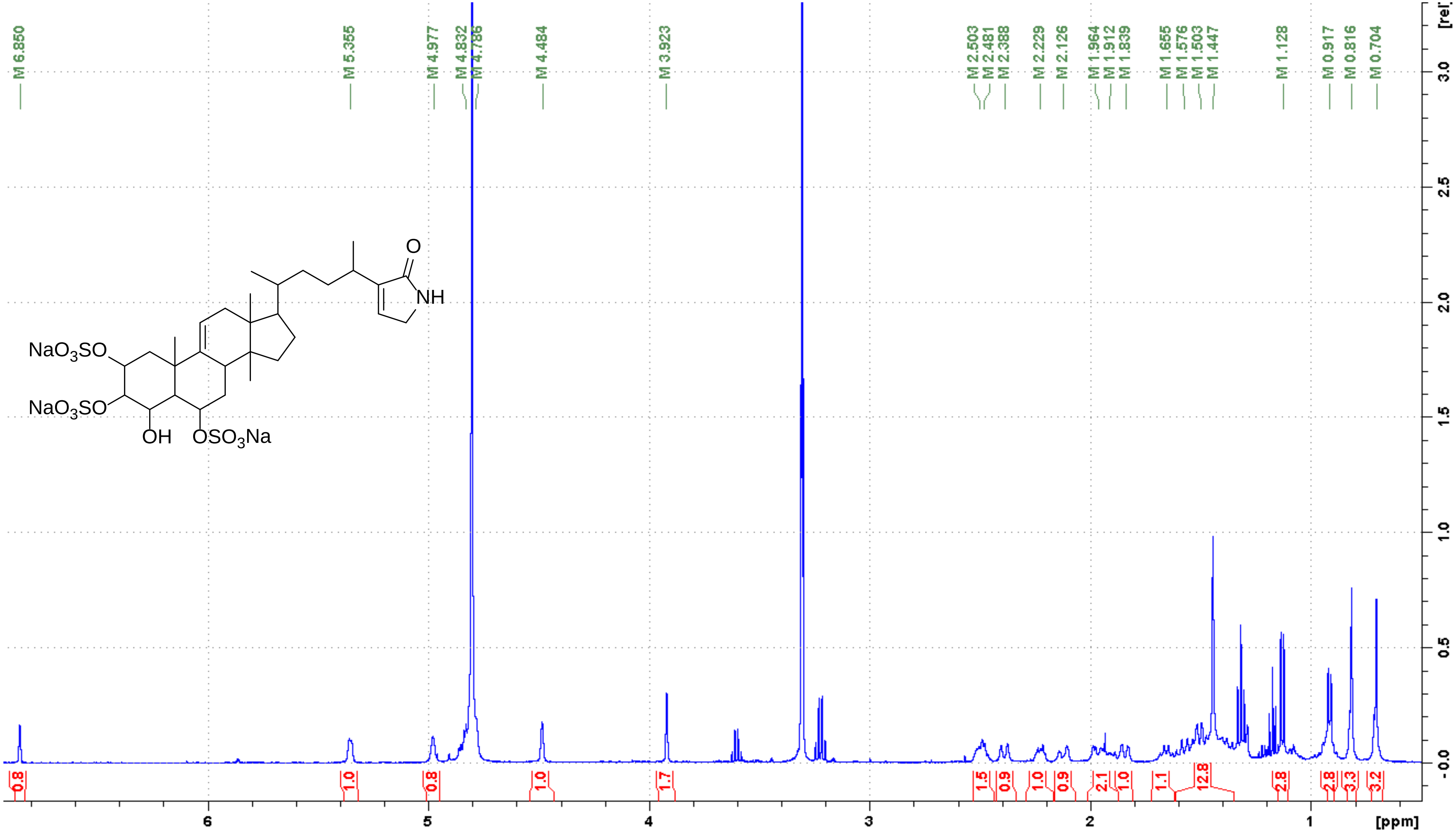

Figure S4. ^{13}C-NMR spectrum of topsentiasterol sulfate G (**1**) in CD_3OD.

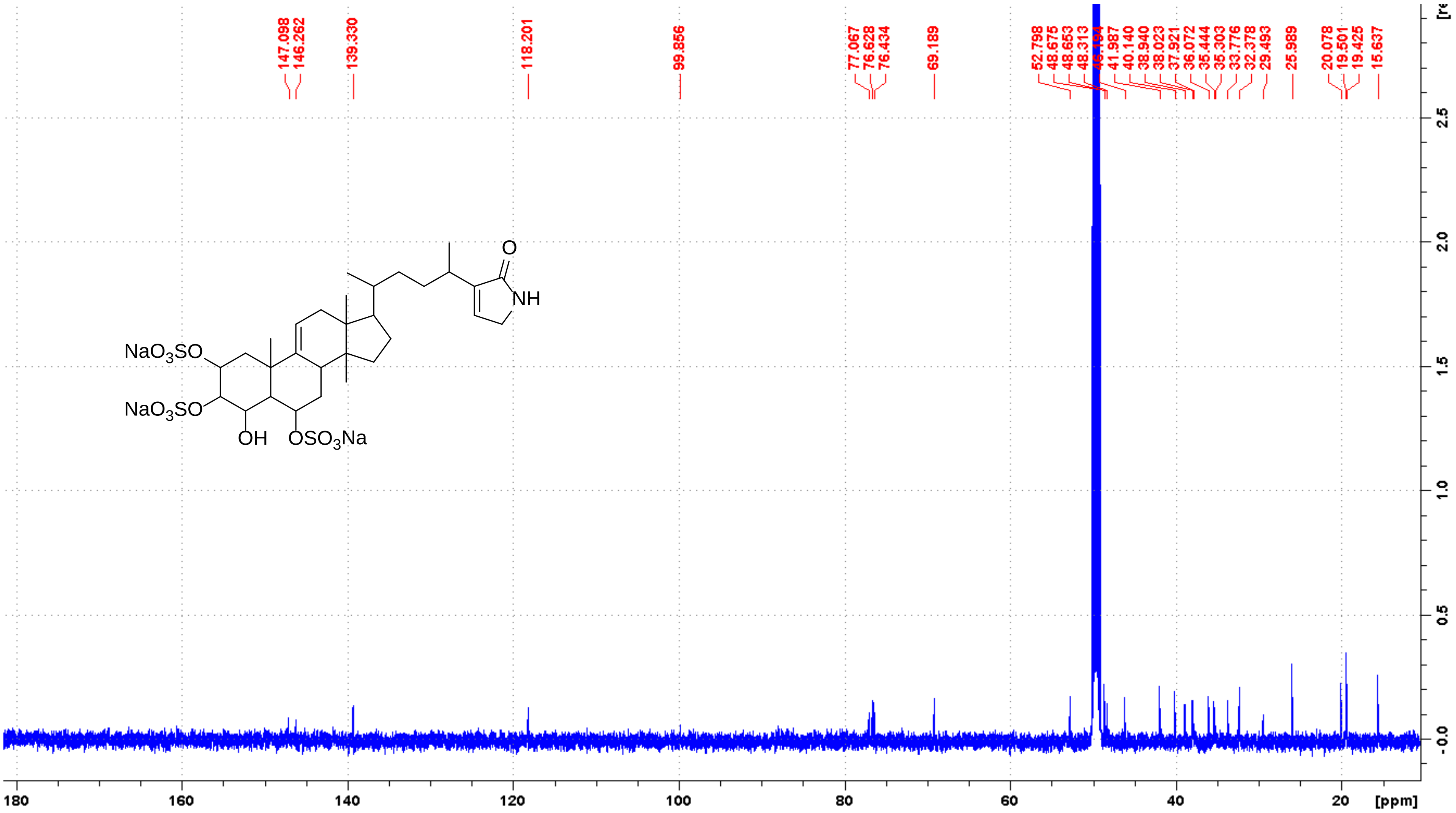

Figure S5. ^1H-^1H-COSY spectrum of topsentiasterol sulfate G (**1**) in CD_3OD.

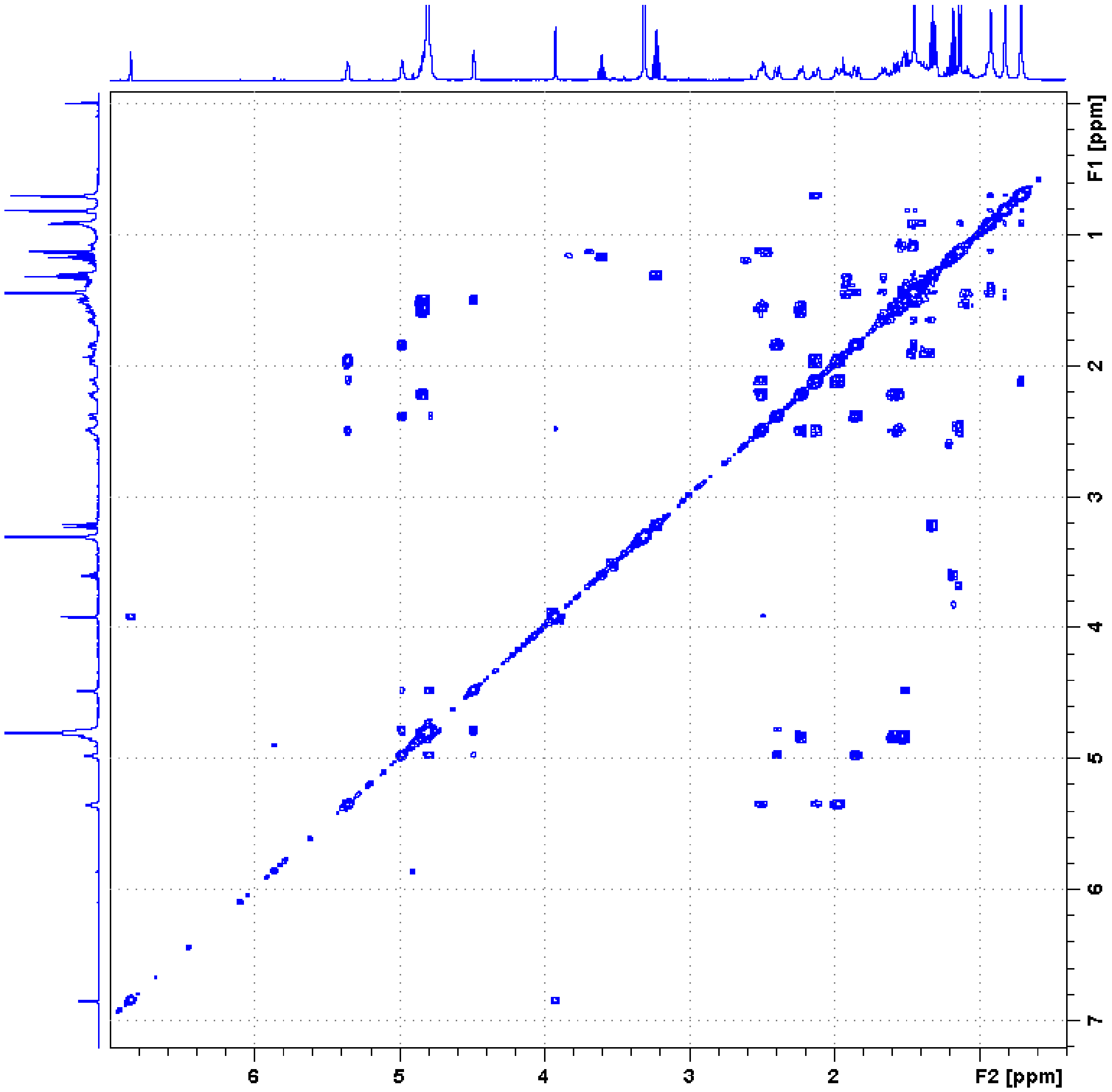

Figure S6. HSQC spectrum of topsentiasterol sulfate G (**1**) in CD_3OD.

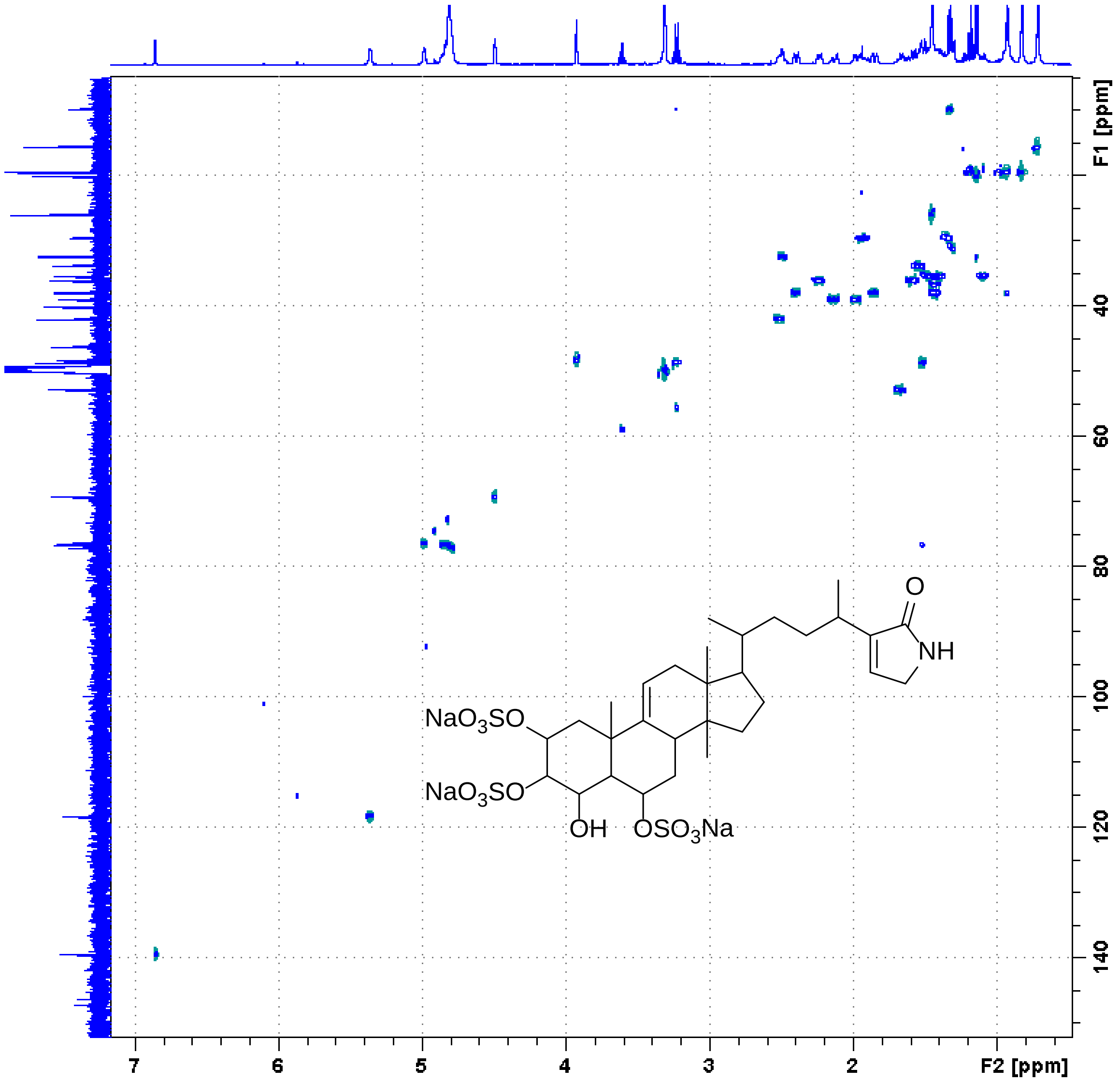

Figure S7. HMBC spectrum of topsentiasterol sulfate G (**1**) in CD3OD.

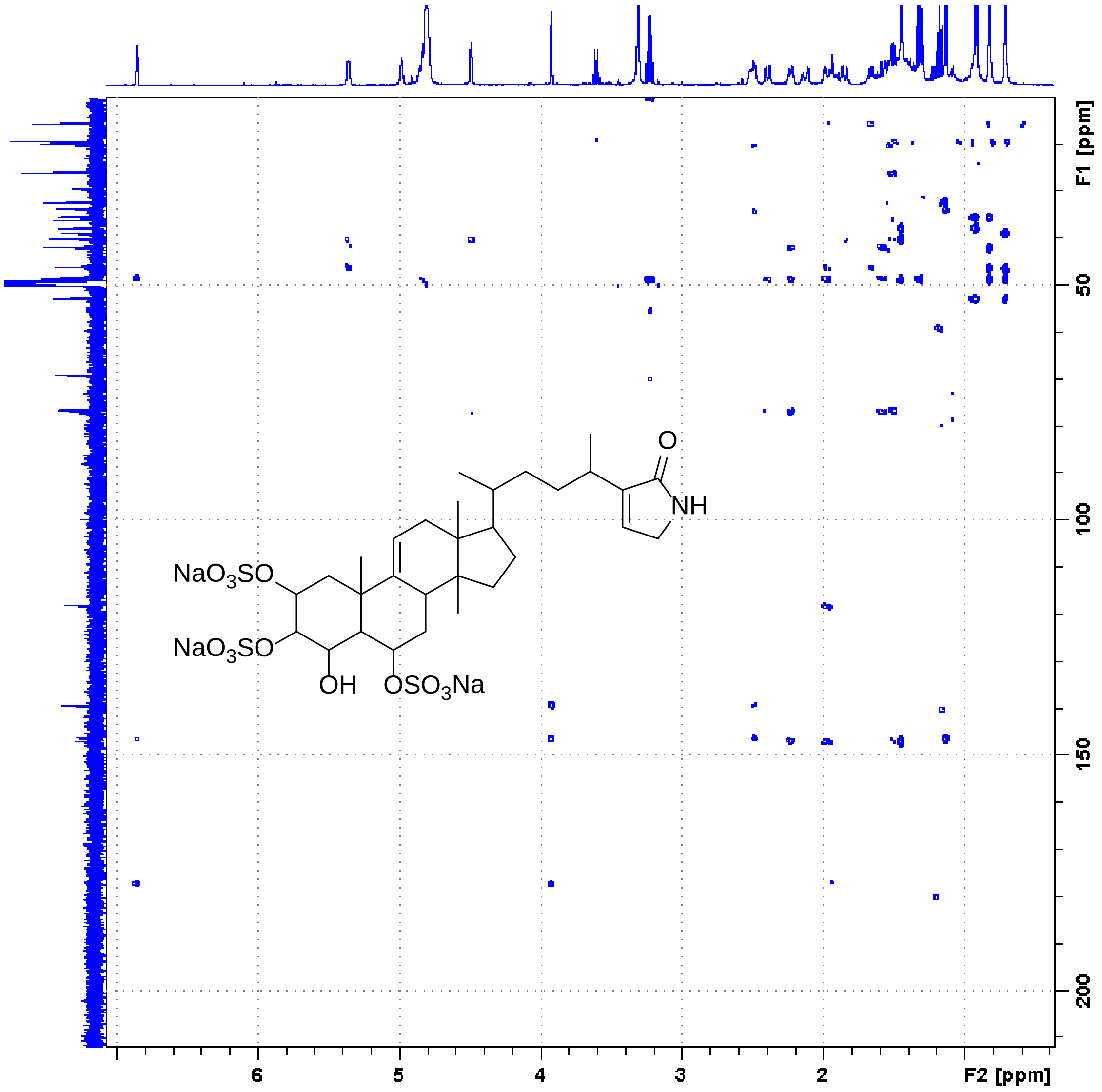

Figure S8. NOESY spectrum of topsentiasterol sulfate G (**1**) in CD3OD.

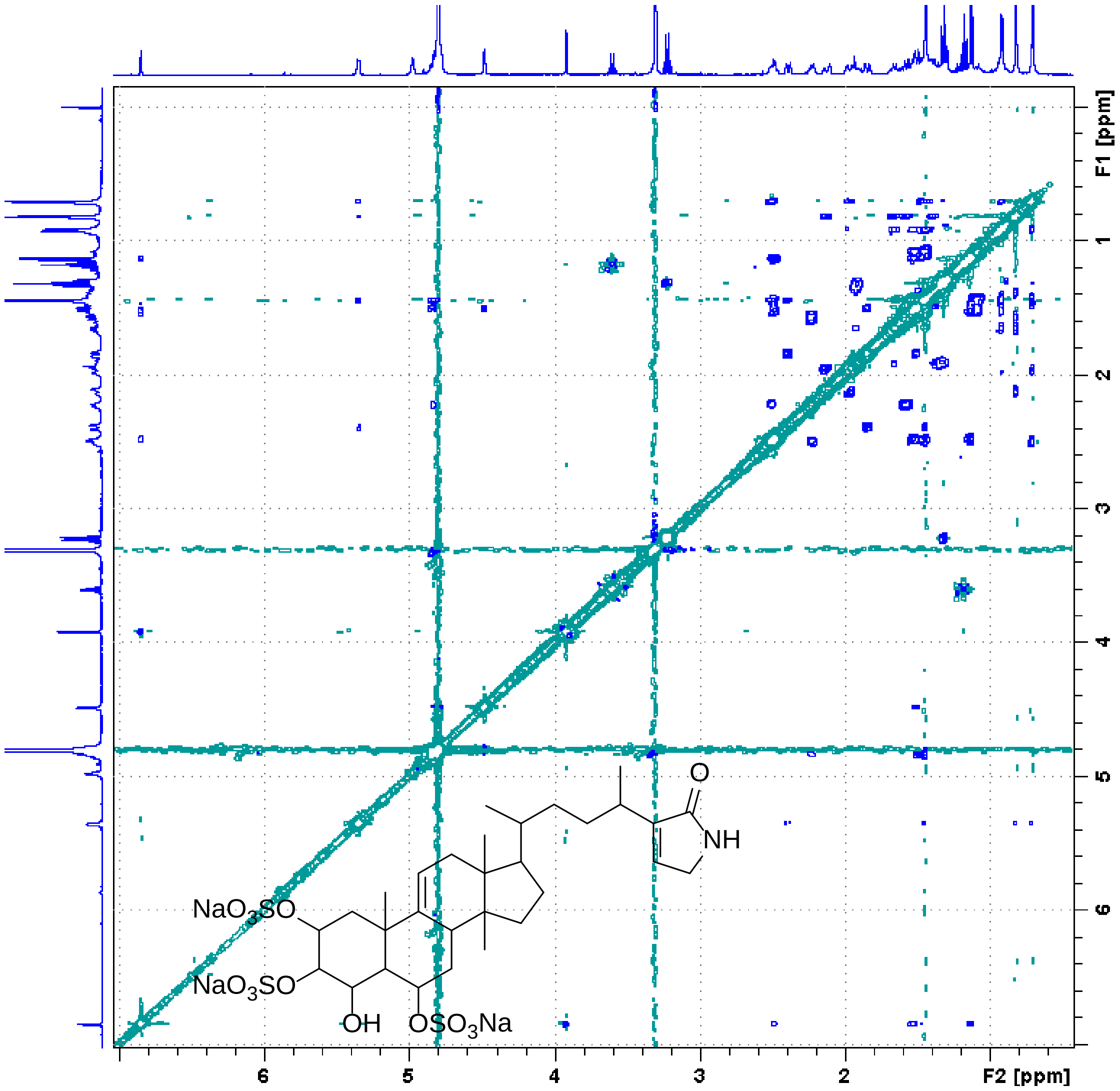

Figure S9. HRESIMS of topsentiasterol sulfate I (**2**).

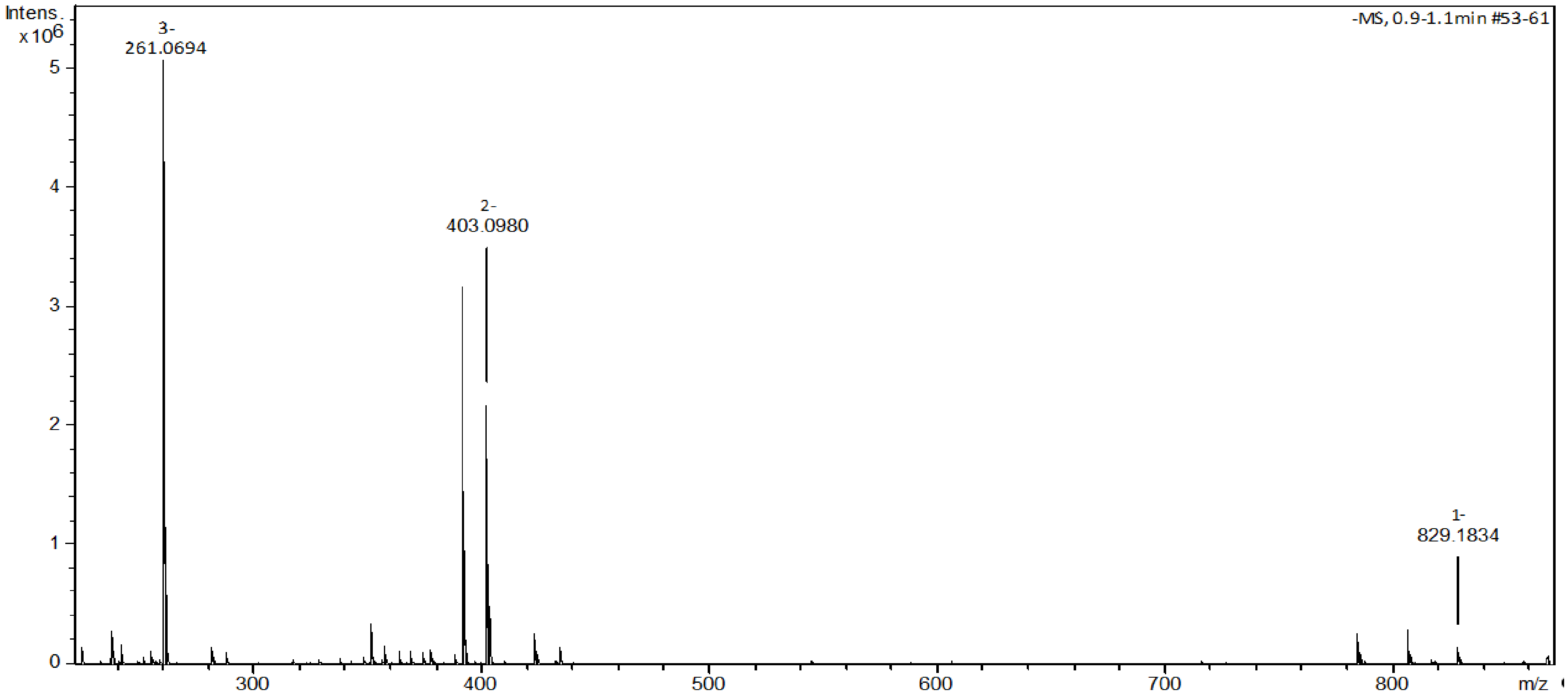

Figure S10. ^{1}H-NMR spectrum of topsentiasterol sulfate I (**2**) in CD_3OD.

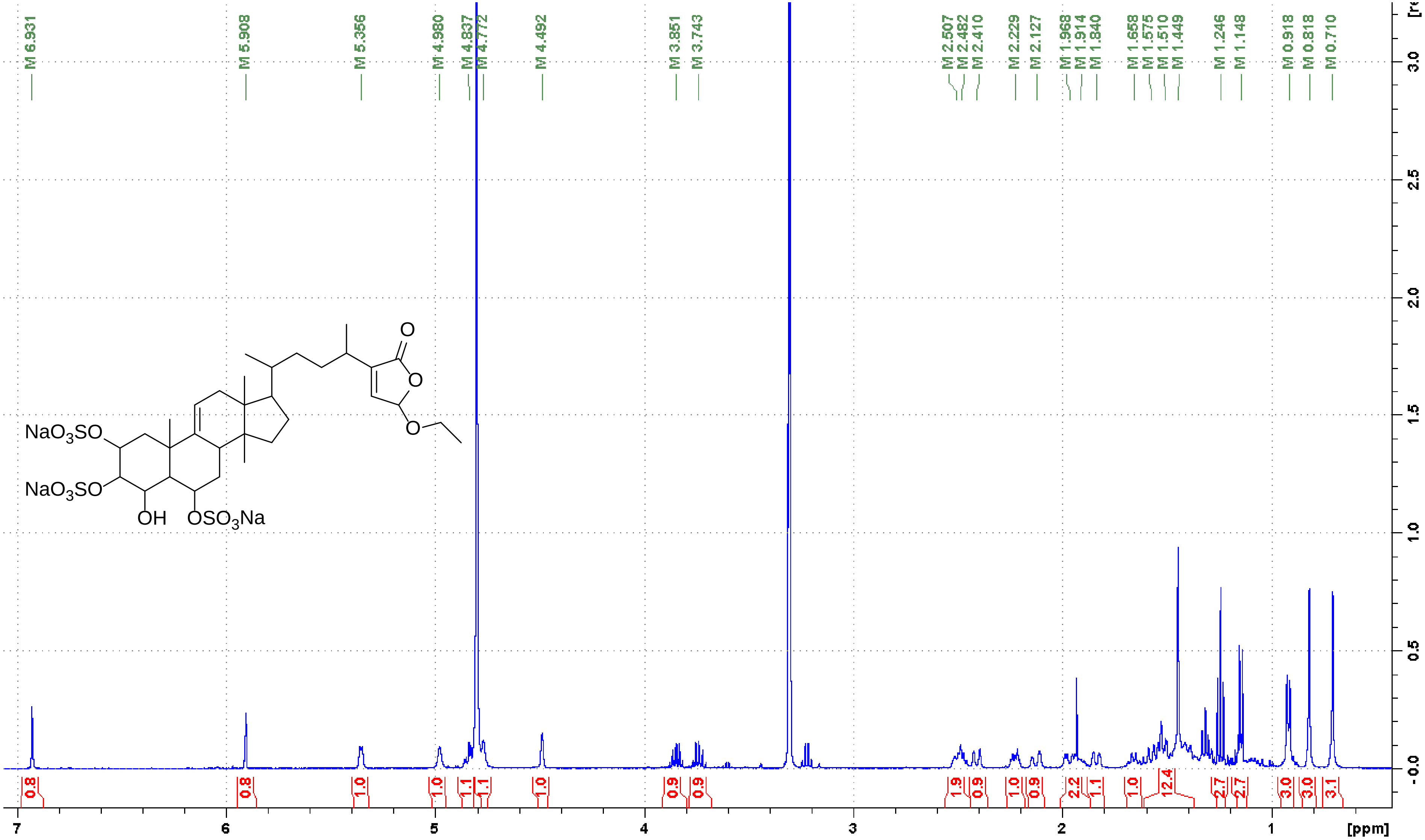

Figure S11. ^{13}C-NMR spectrum of topsentiasterol sulfate I (**2**) in CD_3OD.

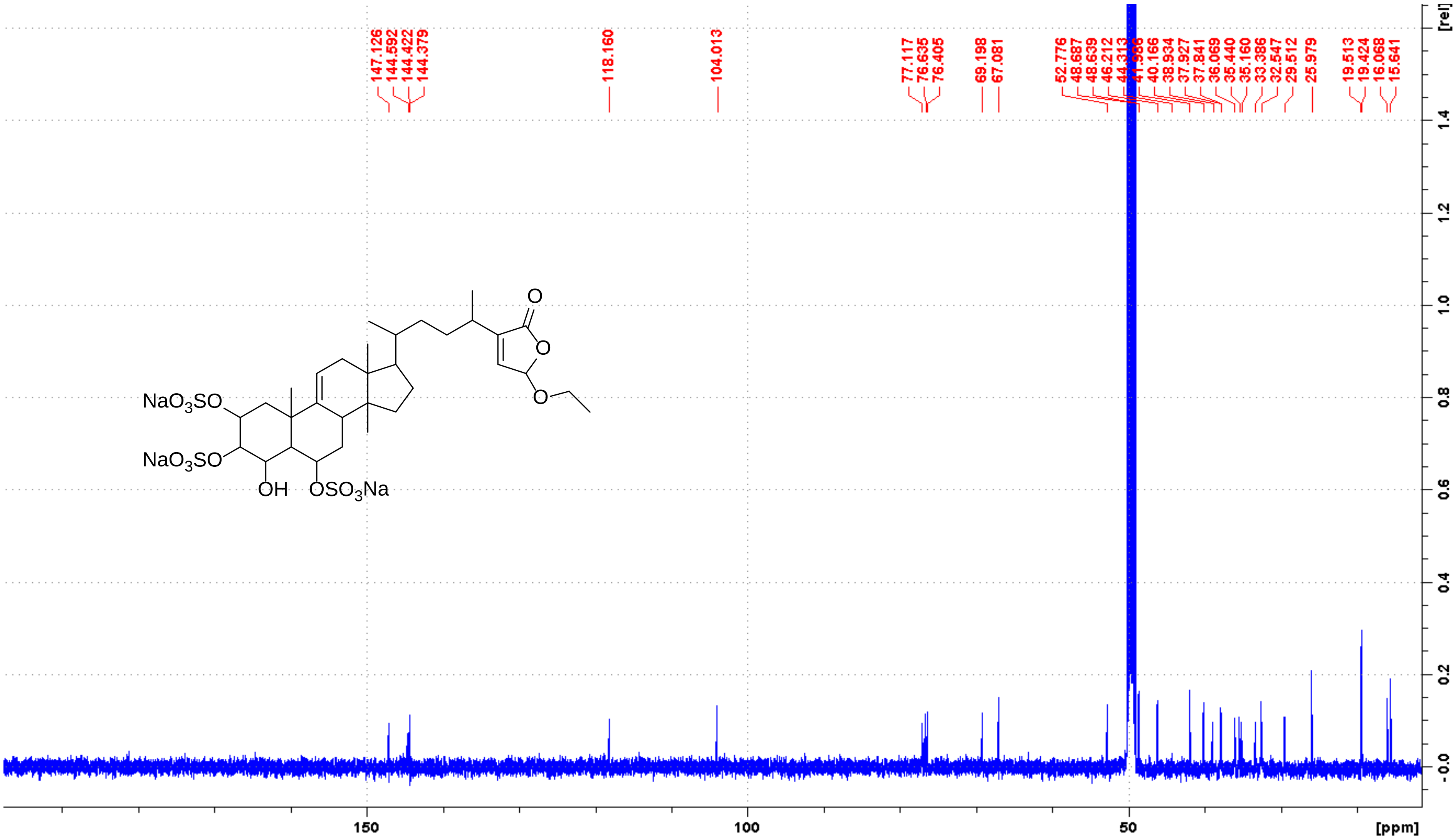

Figure S12. ^{1}H-^{1}H-COSY spectrum of topsentiasterol sulfate I (**2**) in CD_3OD.

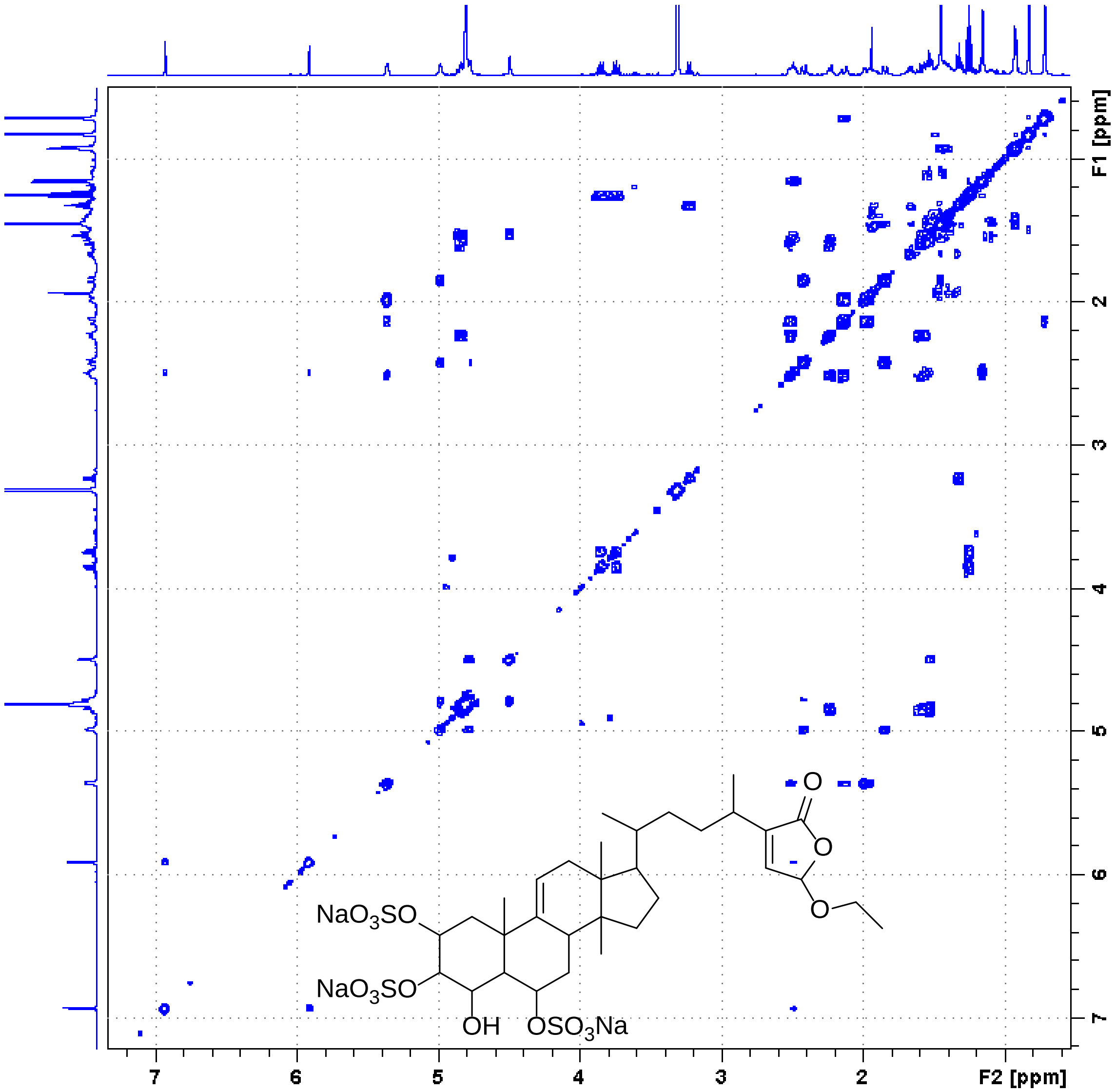

Figure S13. HSQC spectrum of topsentiasterol sulfate I (**2**) in CD3OD.

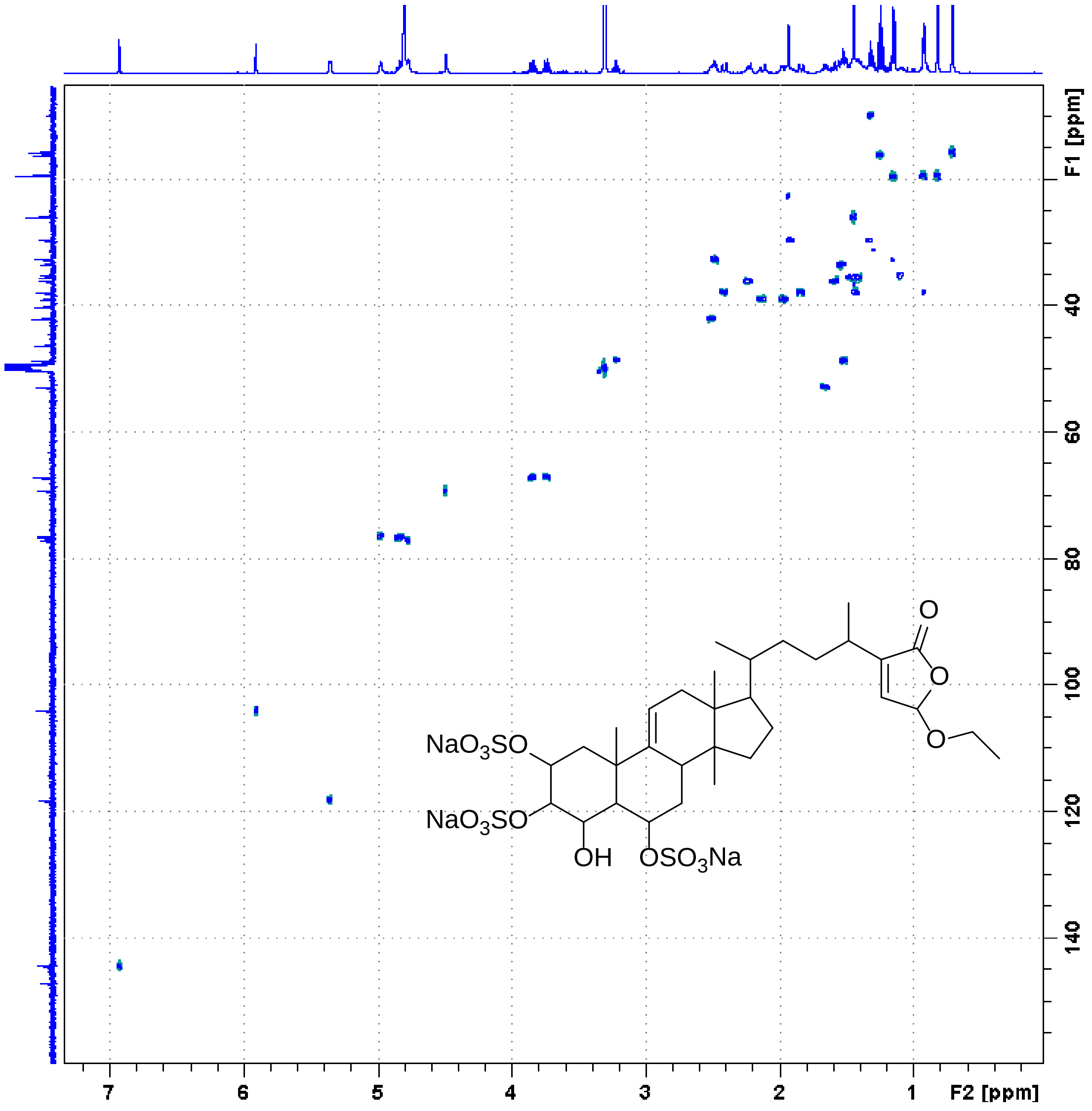

Figure S14. HMBC spectrum of topsentiasterol sulfate I (**2**) in CD_3OD.

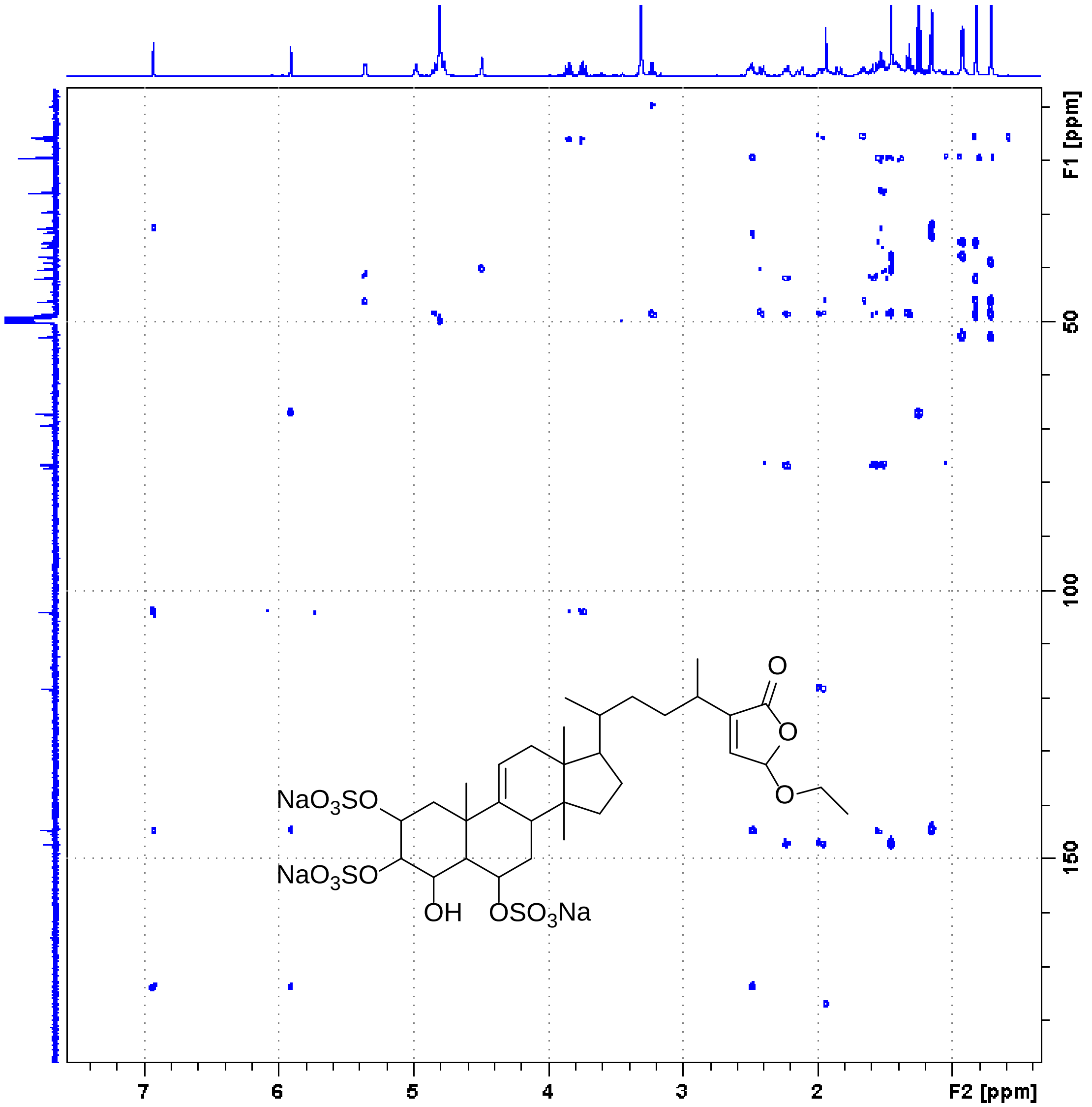

Figure S15. NOESY spectrum of topsentiasterol sulfate I (**2**) in CD_3OD.

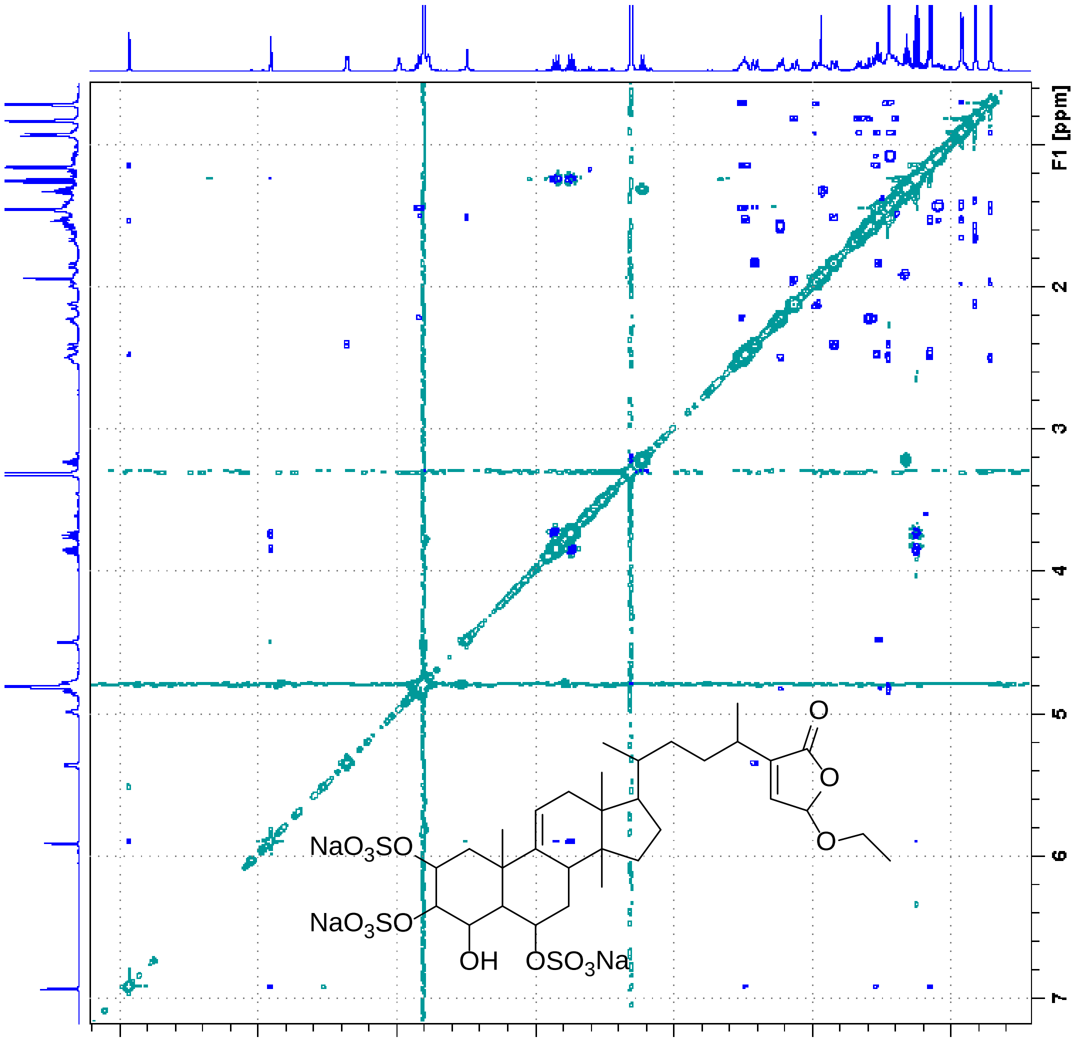

Figure S16. HRESIMS of topsentiasterol sulfate H (**3**).

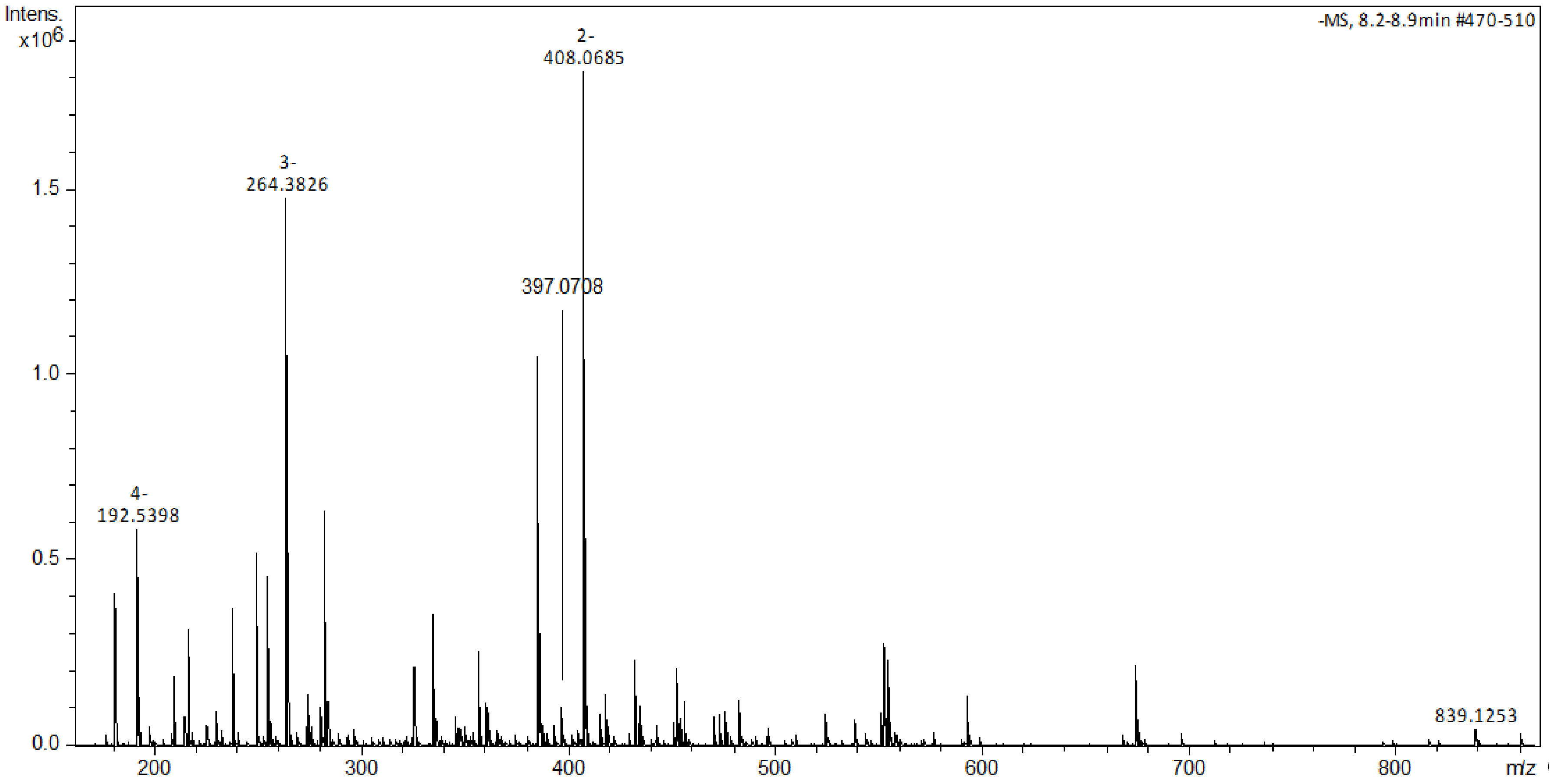

Figure S17. ^{1}H-NMR spectrum of topsentiasterol sulfate H (**3**) in $CD_3OD+CDCl_3$ (~10:1).

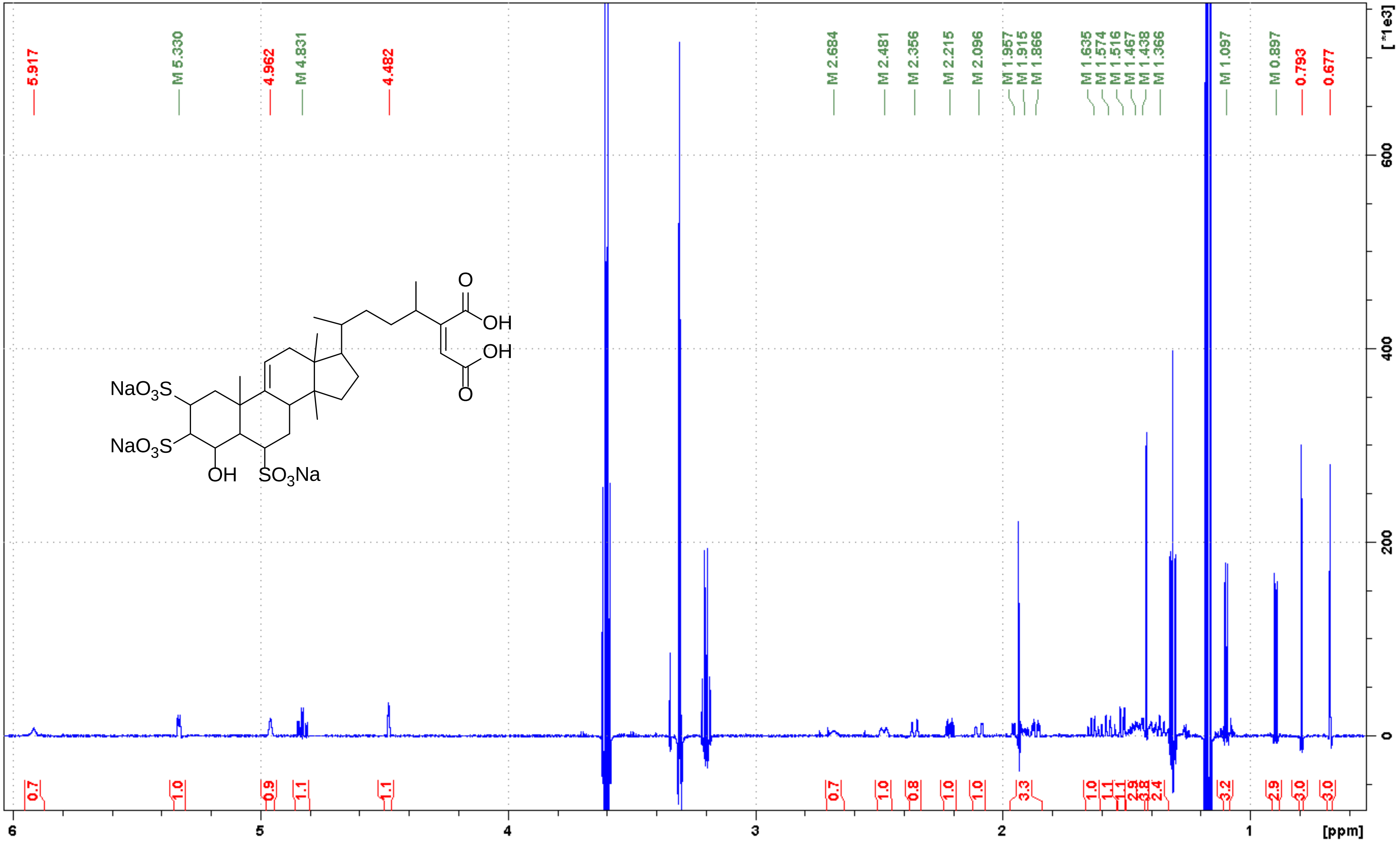

Figure S18. ^{13}C-NMR spectrum of topsentiasterol sulfate H (**3**) in CD_3OD+$CDCl_3$ (~10:1).

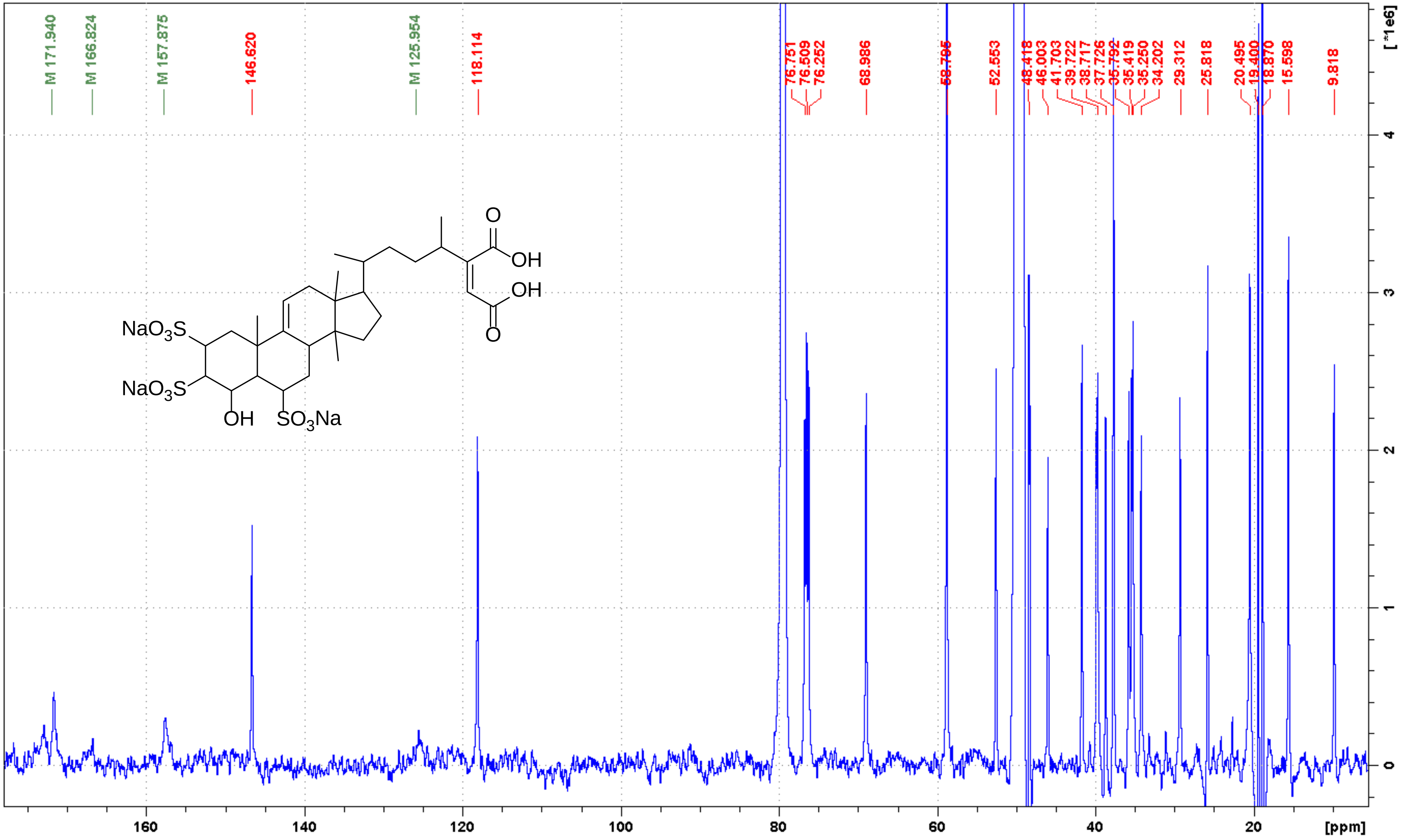

Figure S19. ^{1}H-^{1}H-COSY spectrum of topsentiasterol sulfate H (**3**) in CD_3OD+$CDCl_3$ (~10:1).

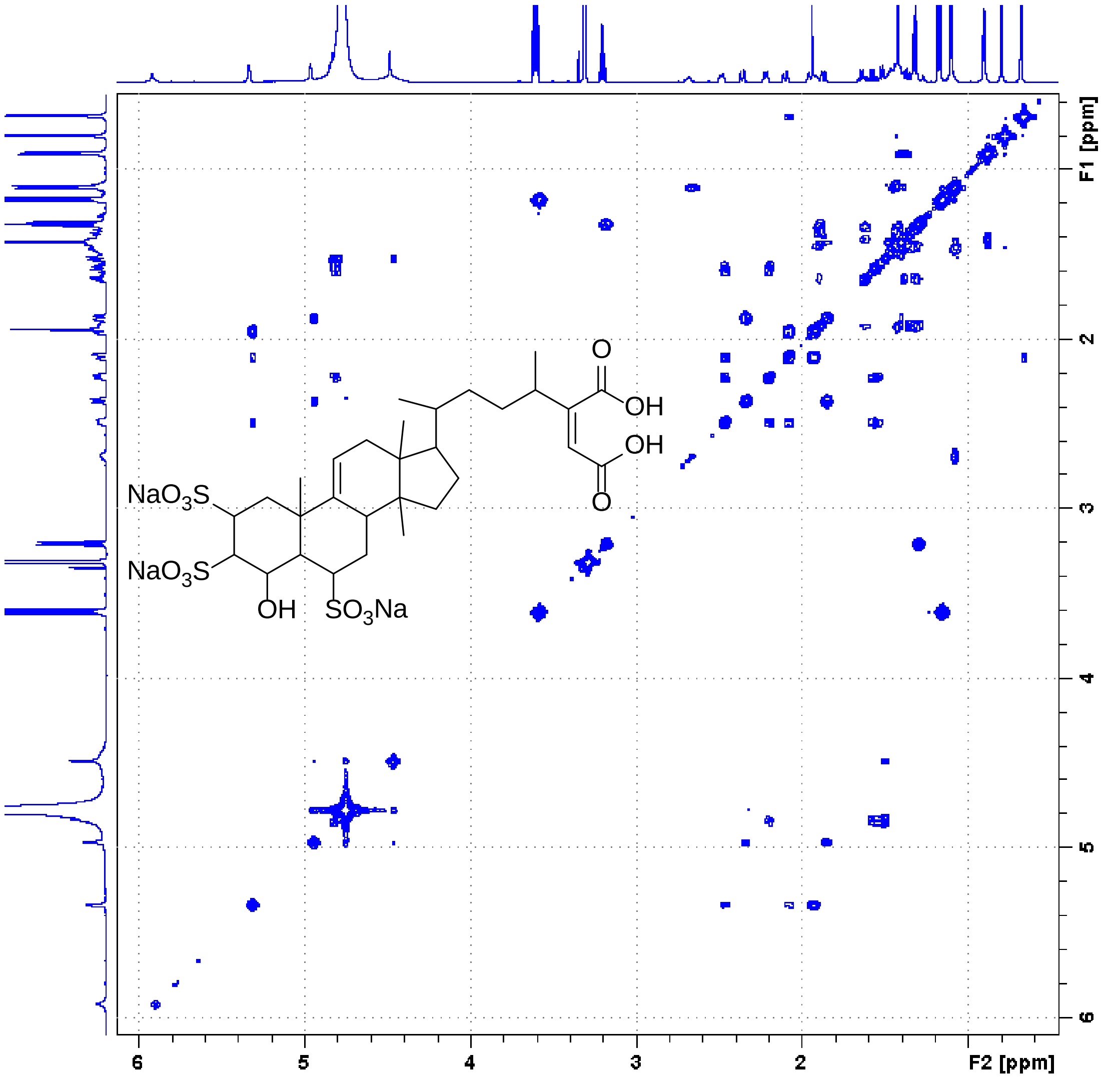

Figure S20. HSQC spectrum of topsentiasterol sulfate H (**3**) in CD_3OD+$CDCl_3$ (~10:1).

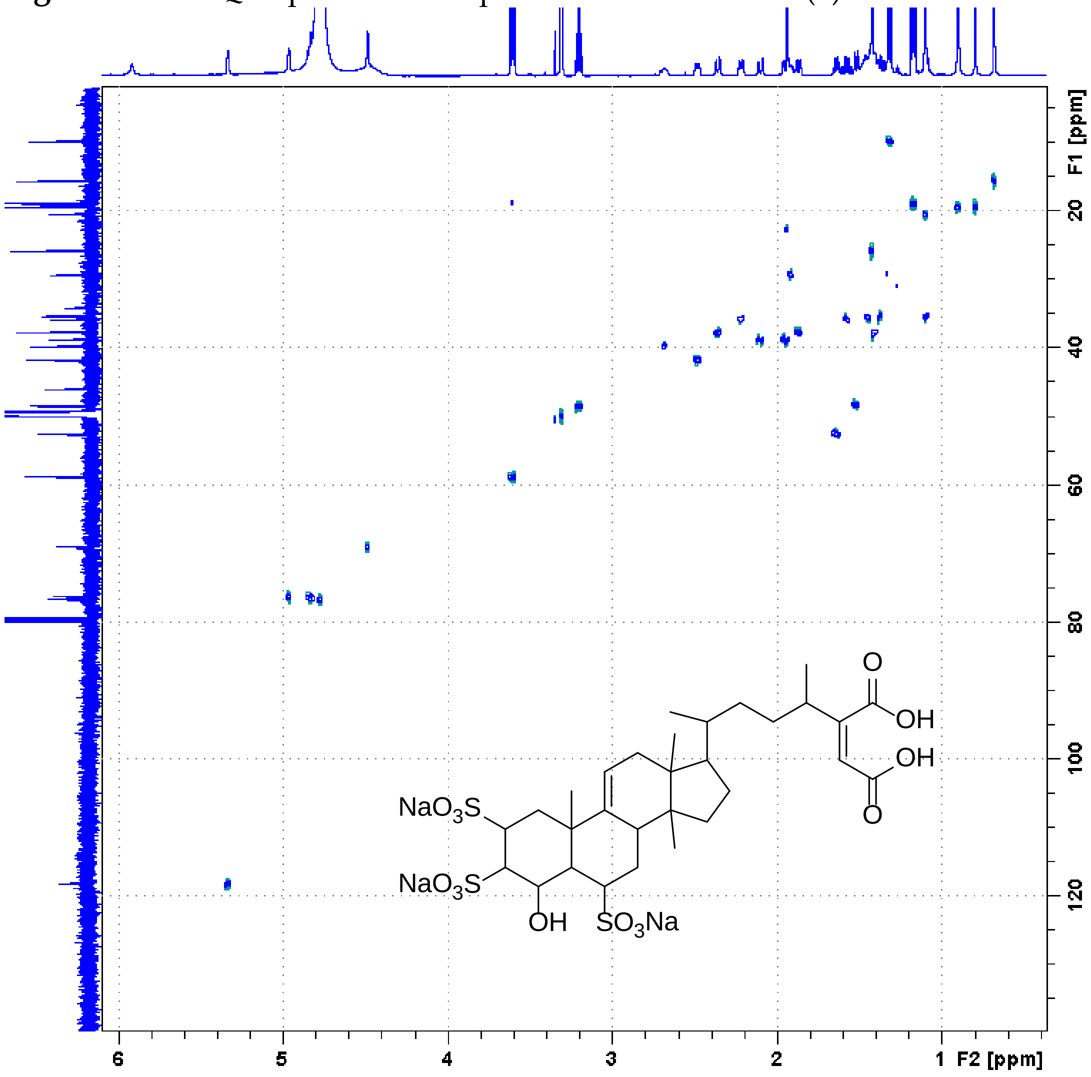

Figure S21. HMBC spectrum of topsentiasterol sulfate H (**3**) in DMSO-d_6.

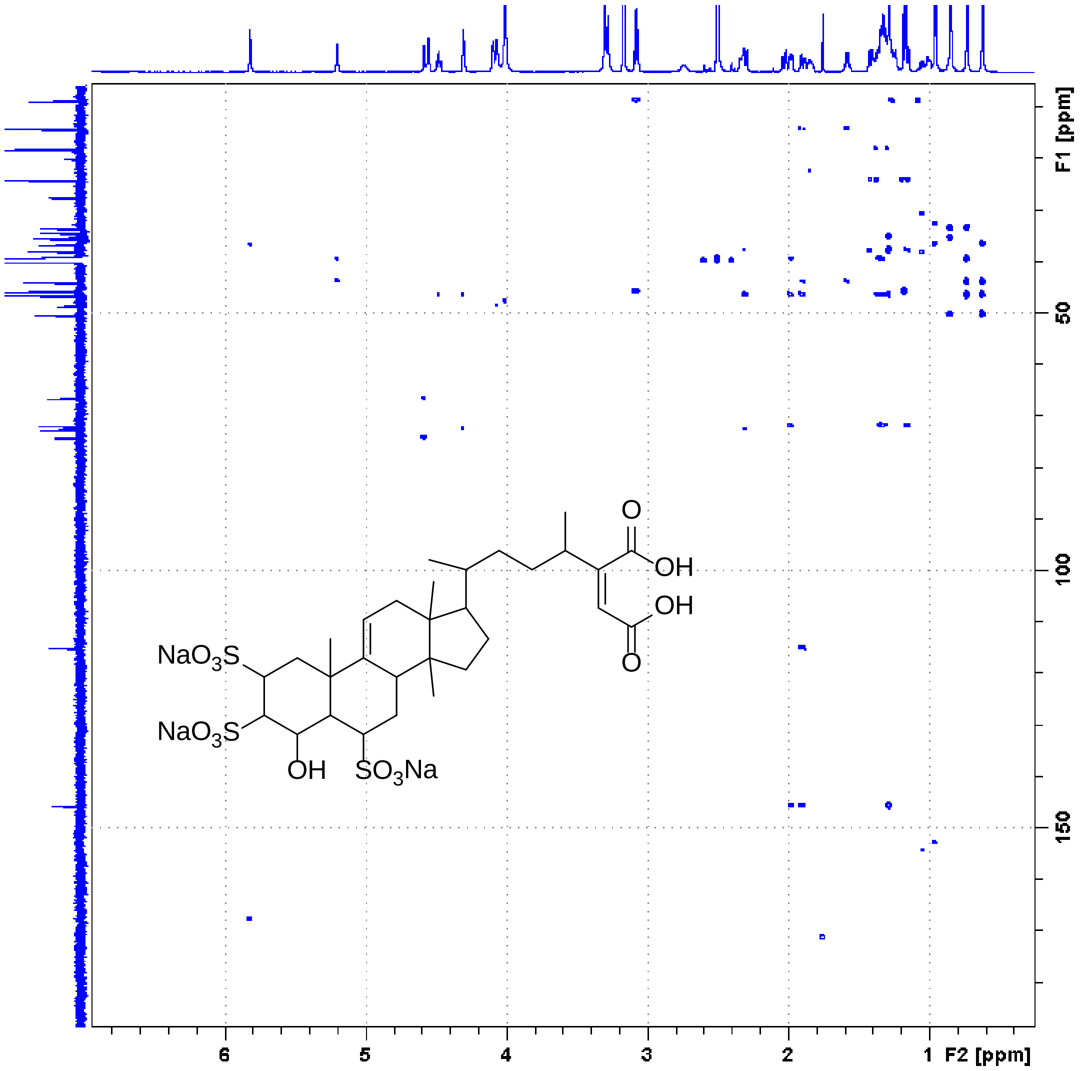

Figure S22. NOESY spectrum of topsentiasterol sulfate H (**3**) in $CD_3OD+CDCl_3$ (~10:1).

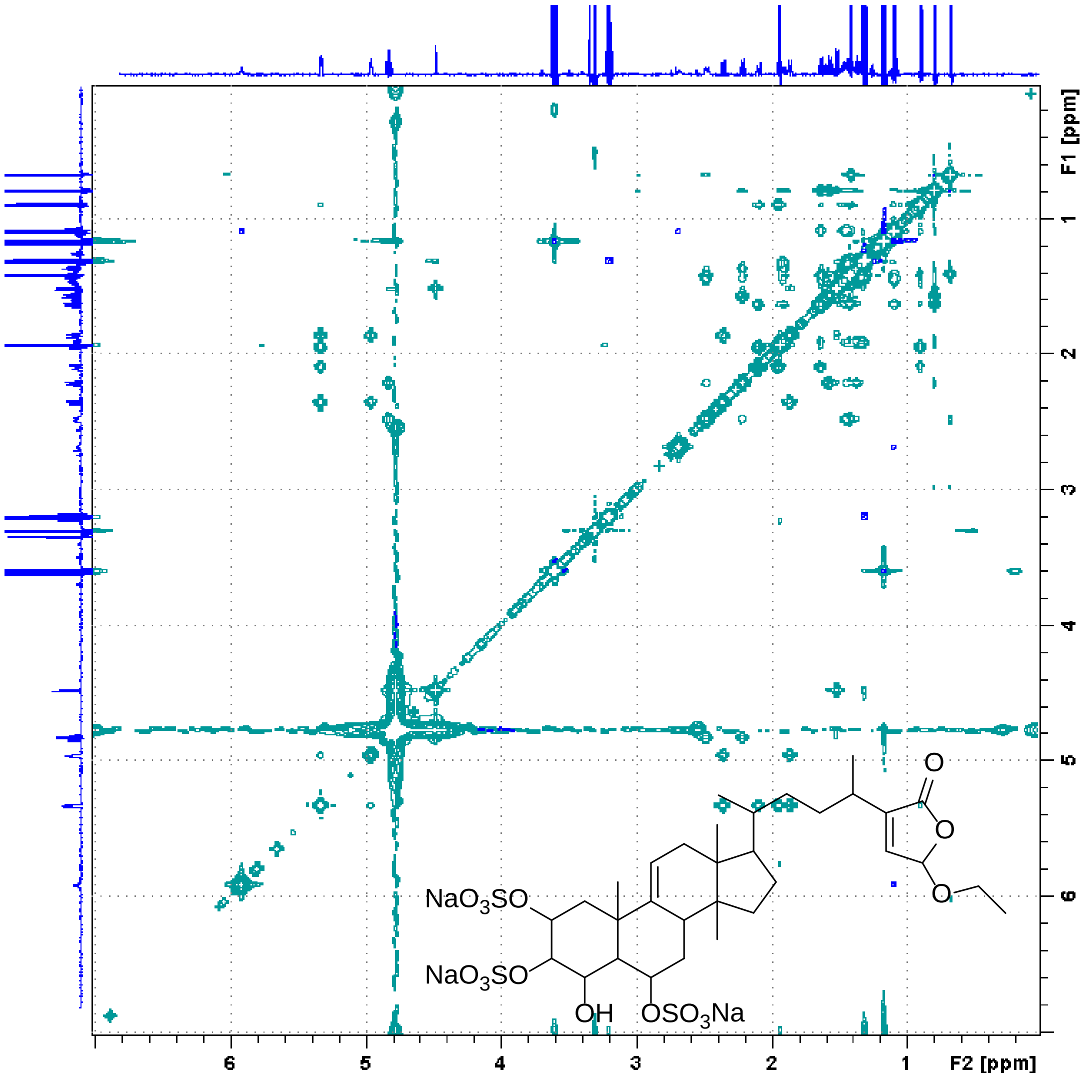

Figure S23. HRESIMS of the topsentiasterol sulfate H methylation reaction product.

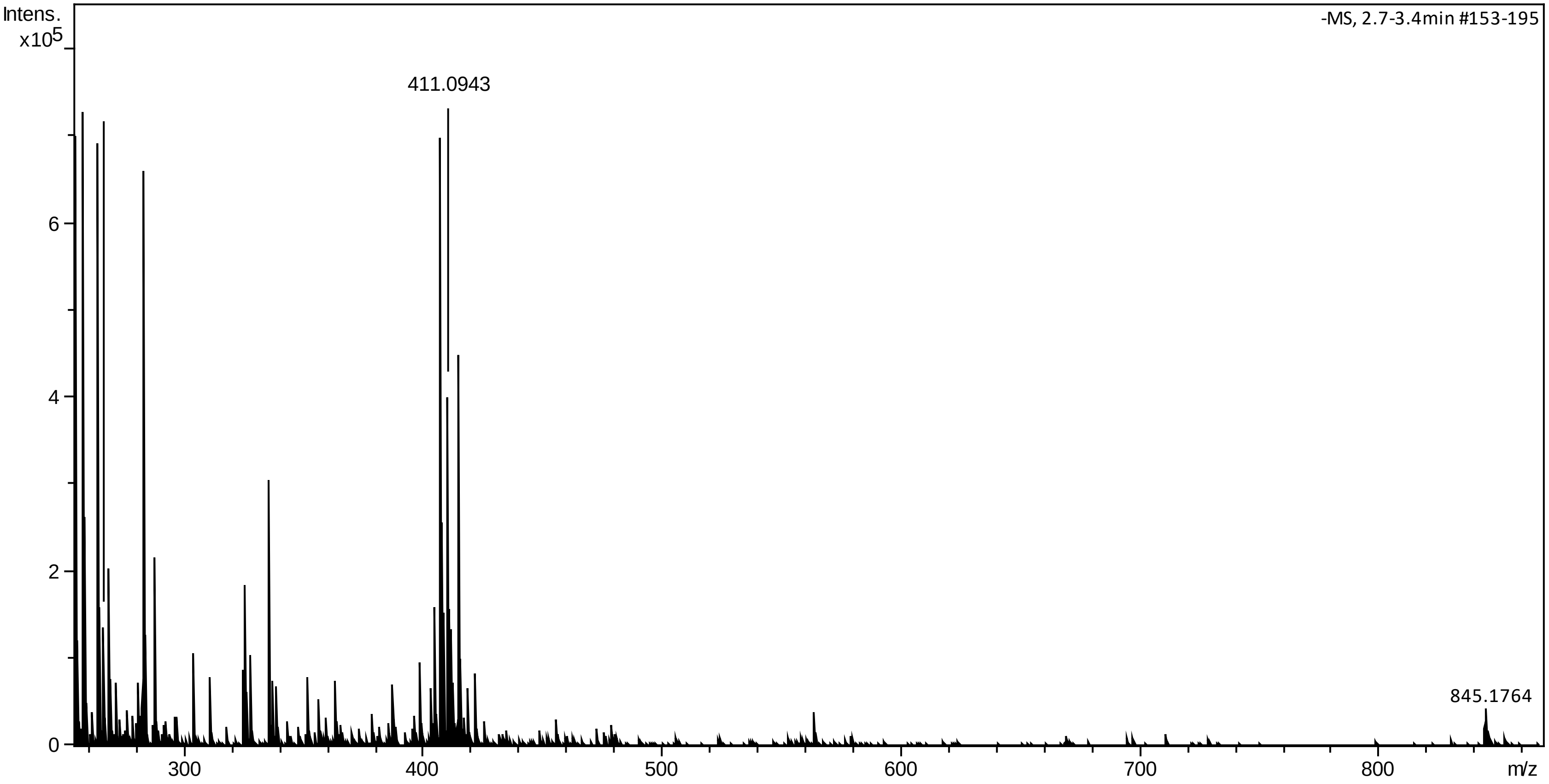

Figure S24. HRESIMS of the topsentiasterol sulfate H desulfation reaction product (**11**).

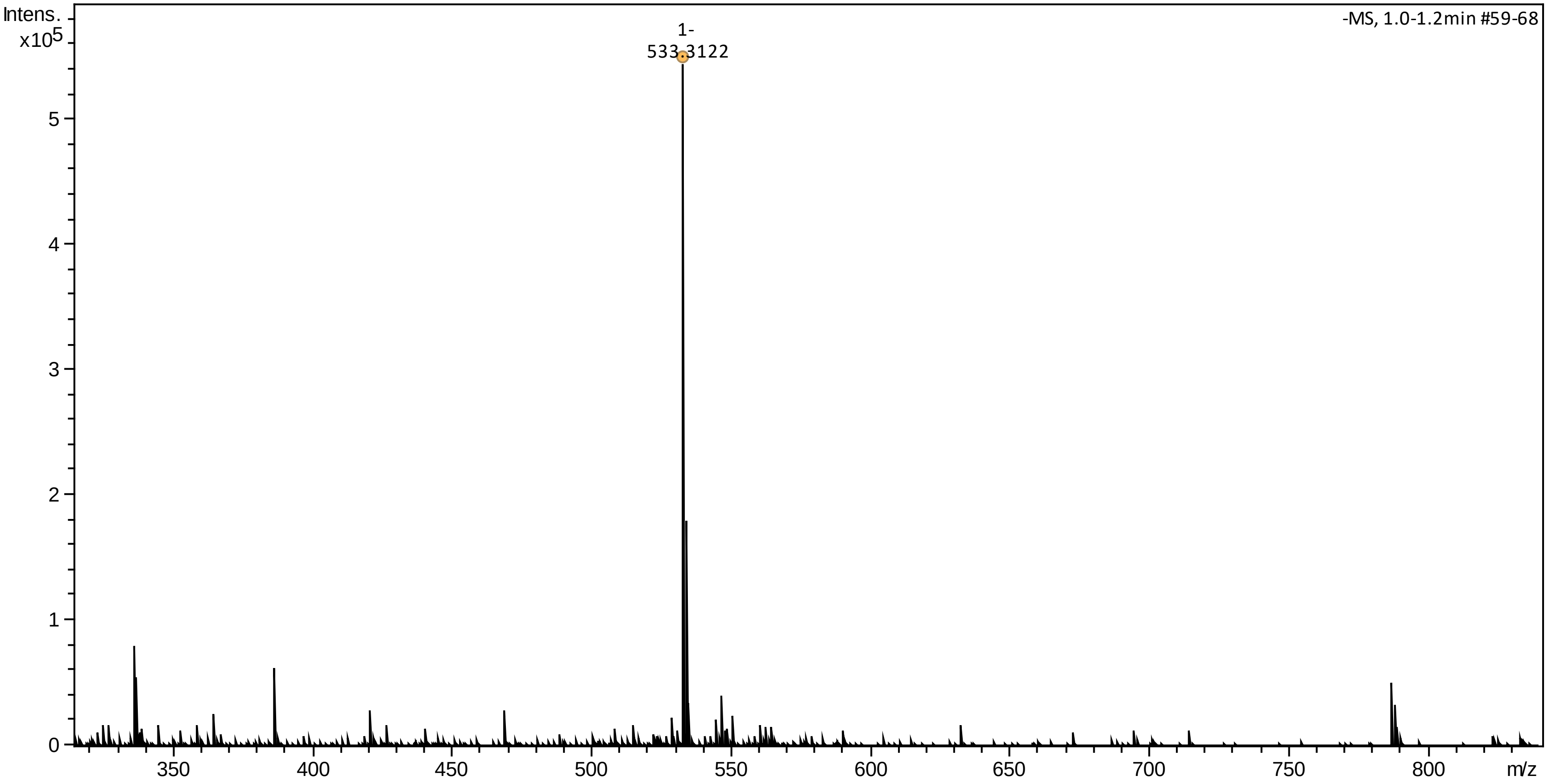

Figure S25. HRESIMS of bromotopsentiasterol sulfate D (**4**) (in mixture with **5** and **6**).

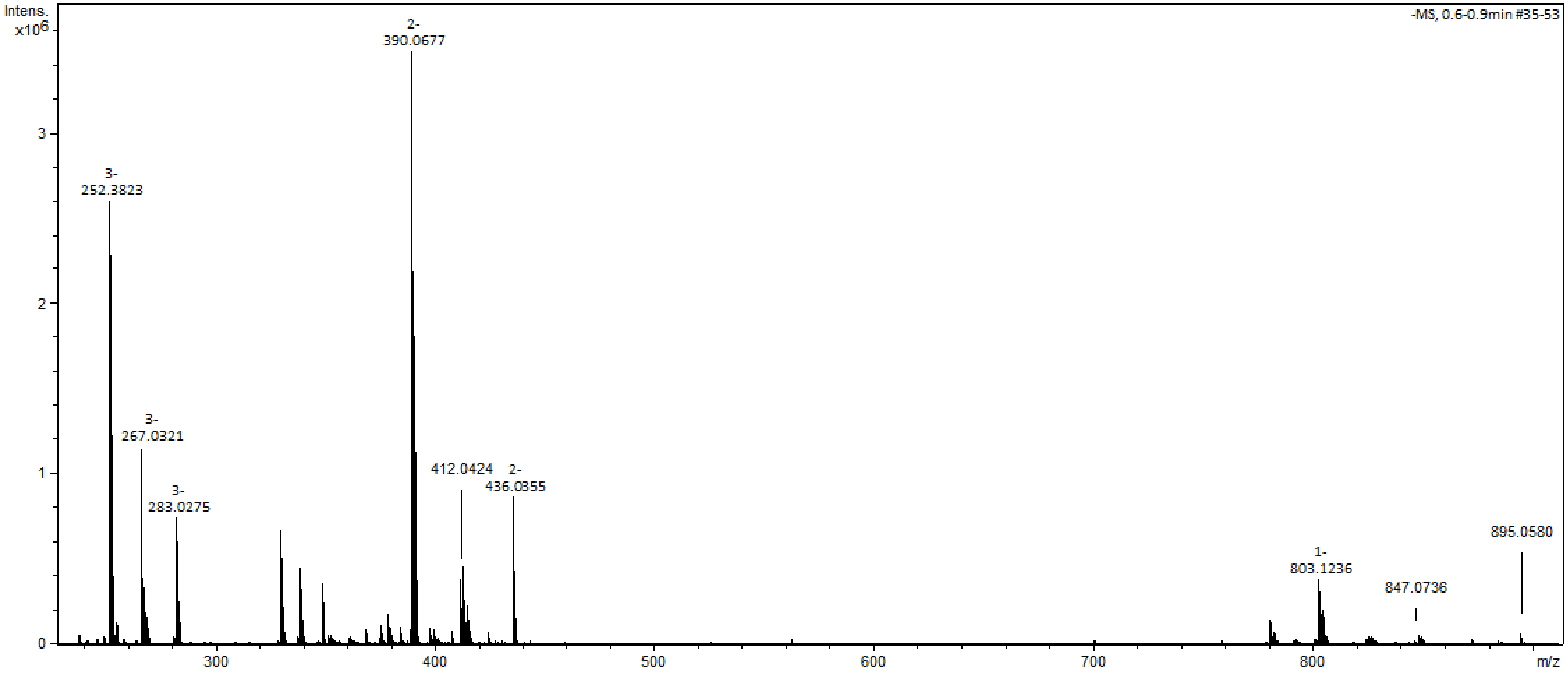

Figure S26. ^{1}H-NMR spectrum of bromotopsentiasterol sulfate D (**4**) (in mixture with **5** and **6**) in CD_3OD.

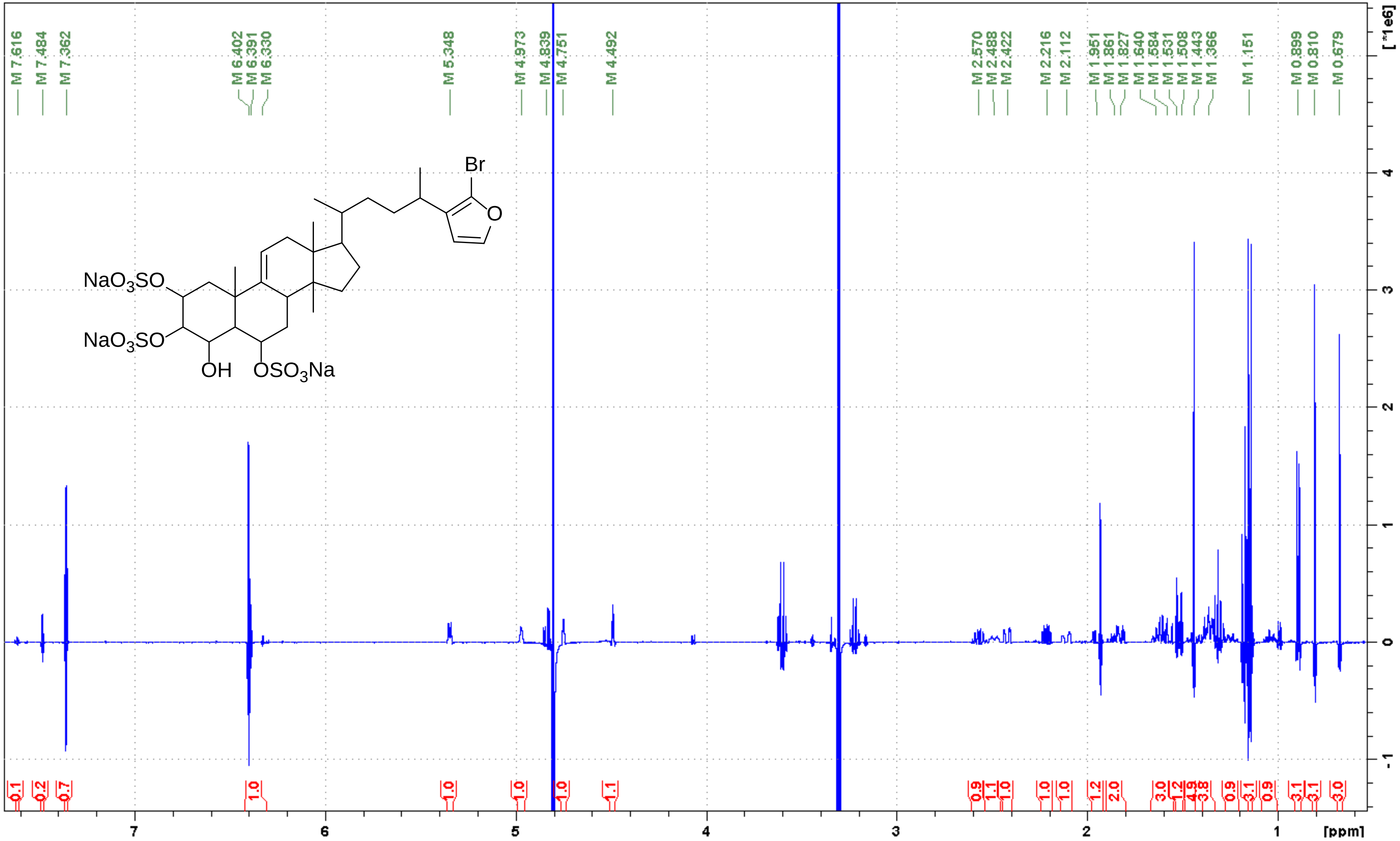

Figure S27. ^{13}C-NMR spectrum of bromotopsentiasterol sulfate D (**4**) (in mixture with **5** and **6**) in CD3OD.

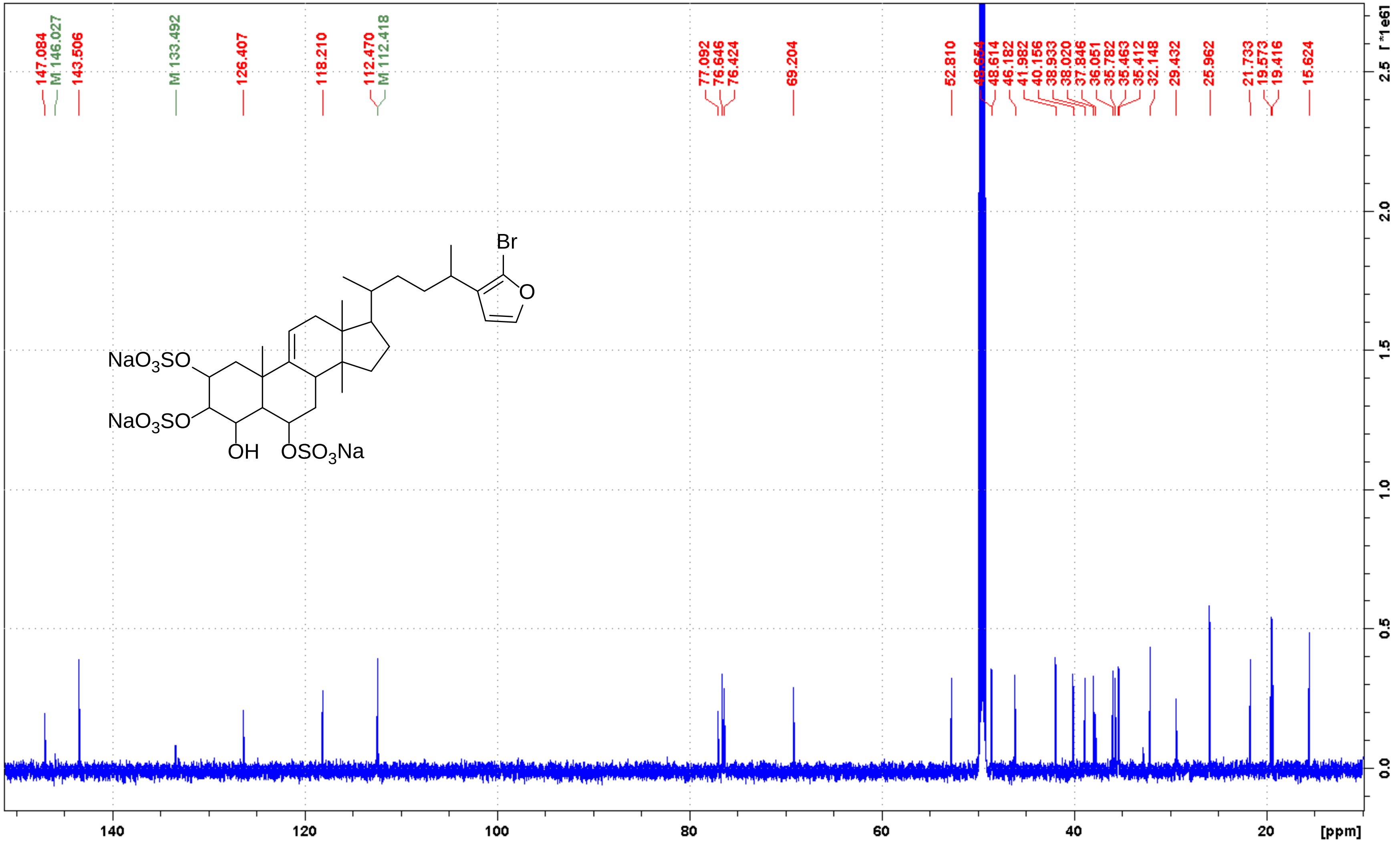

Figure S28. ¹H-¹H-COSY spectrum of bromotopsentiasterol sulfate D (**4**) (in mixture with **5** and **6**) in CD₃OD.

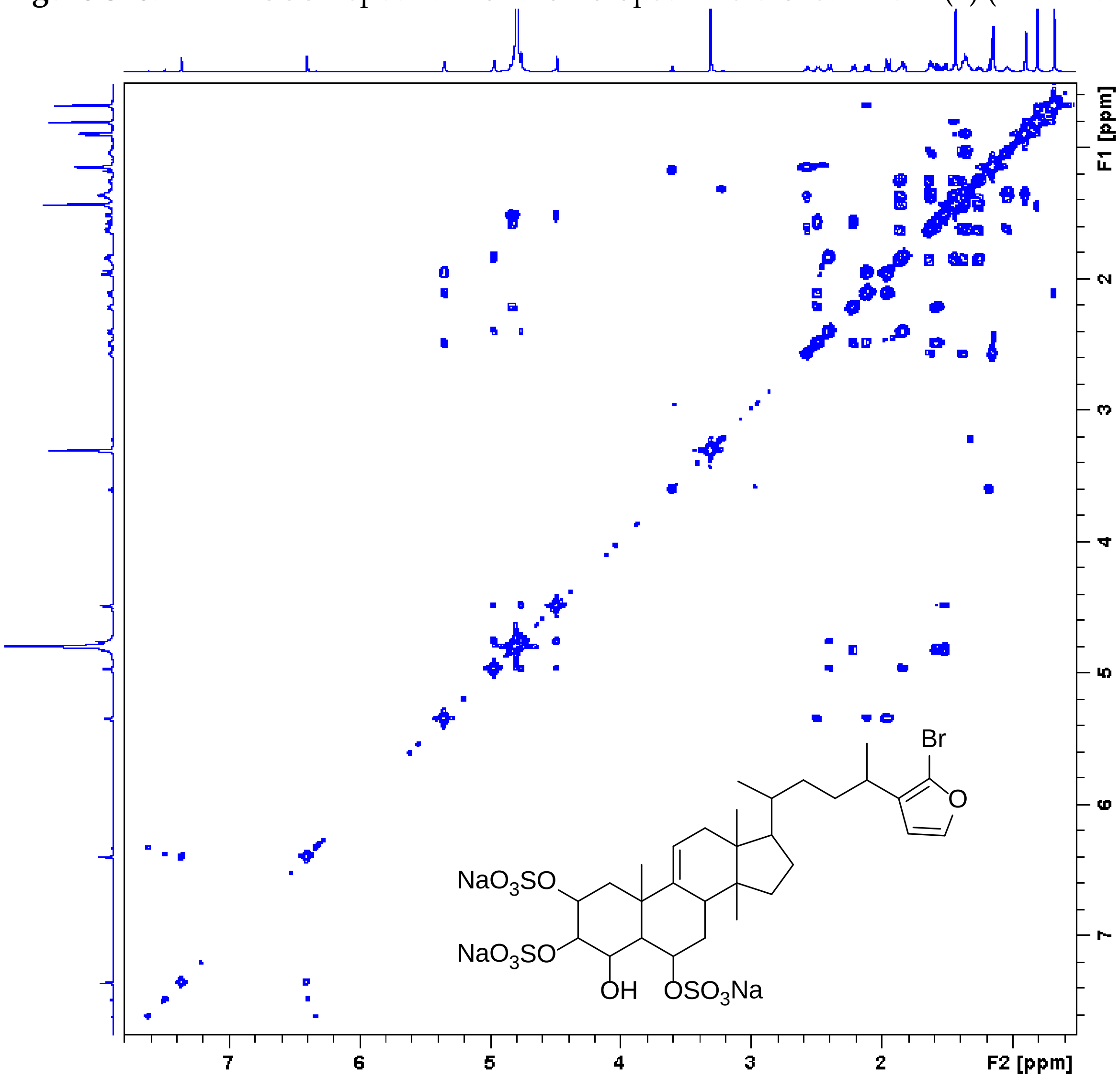

Figure S29. HSQC spectrum of bromotopsentiasterol sulfate D (**4**) (in mixture with **5** and **6**) in CD3OD.

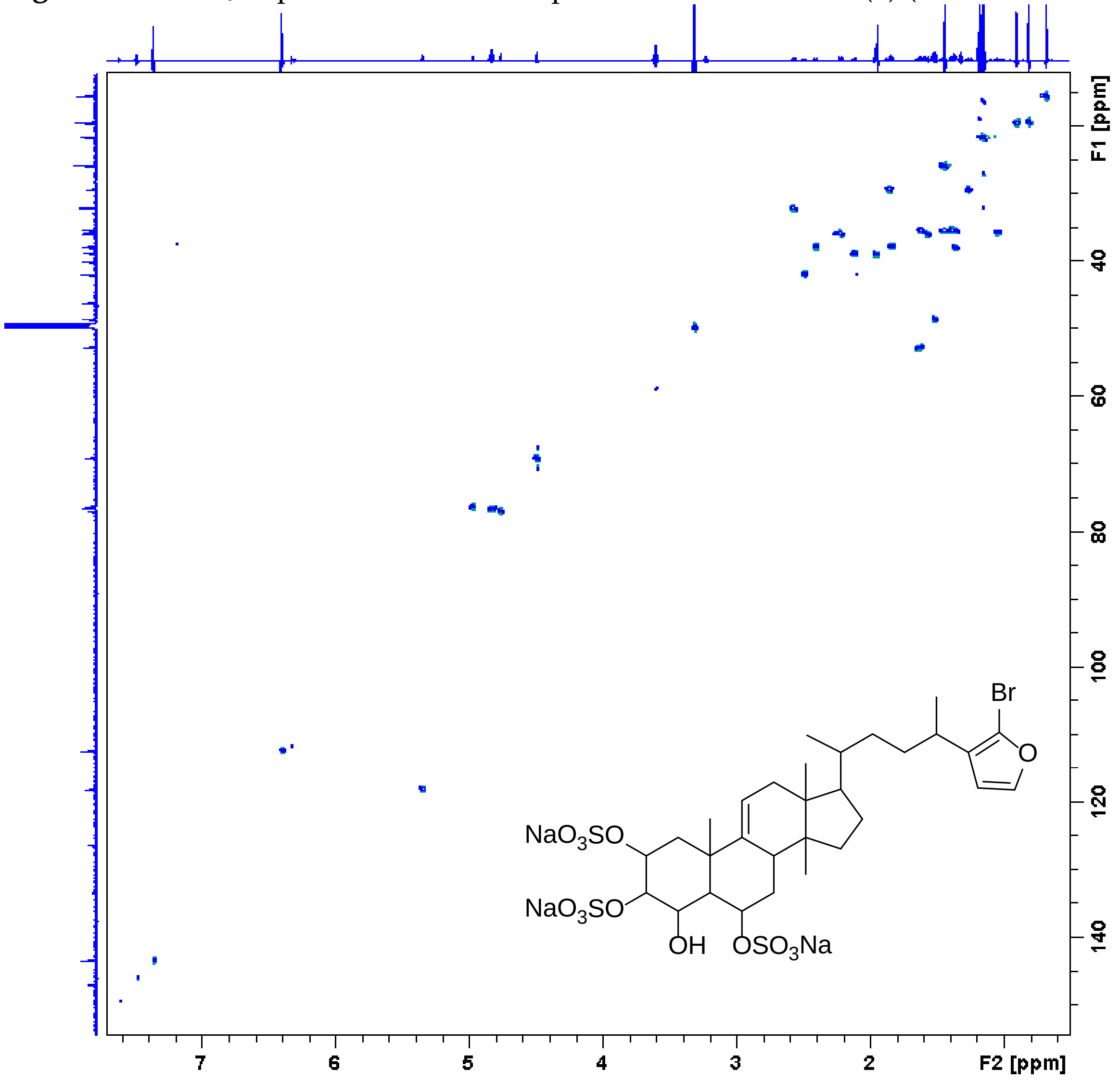

Figure S30. HMBC spectrum of bromotopsentiasterol sulfate D (**4**) (in mixture with **5** and **6**) in CD_3OD.

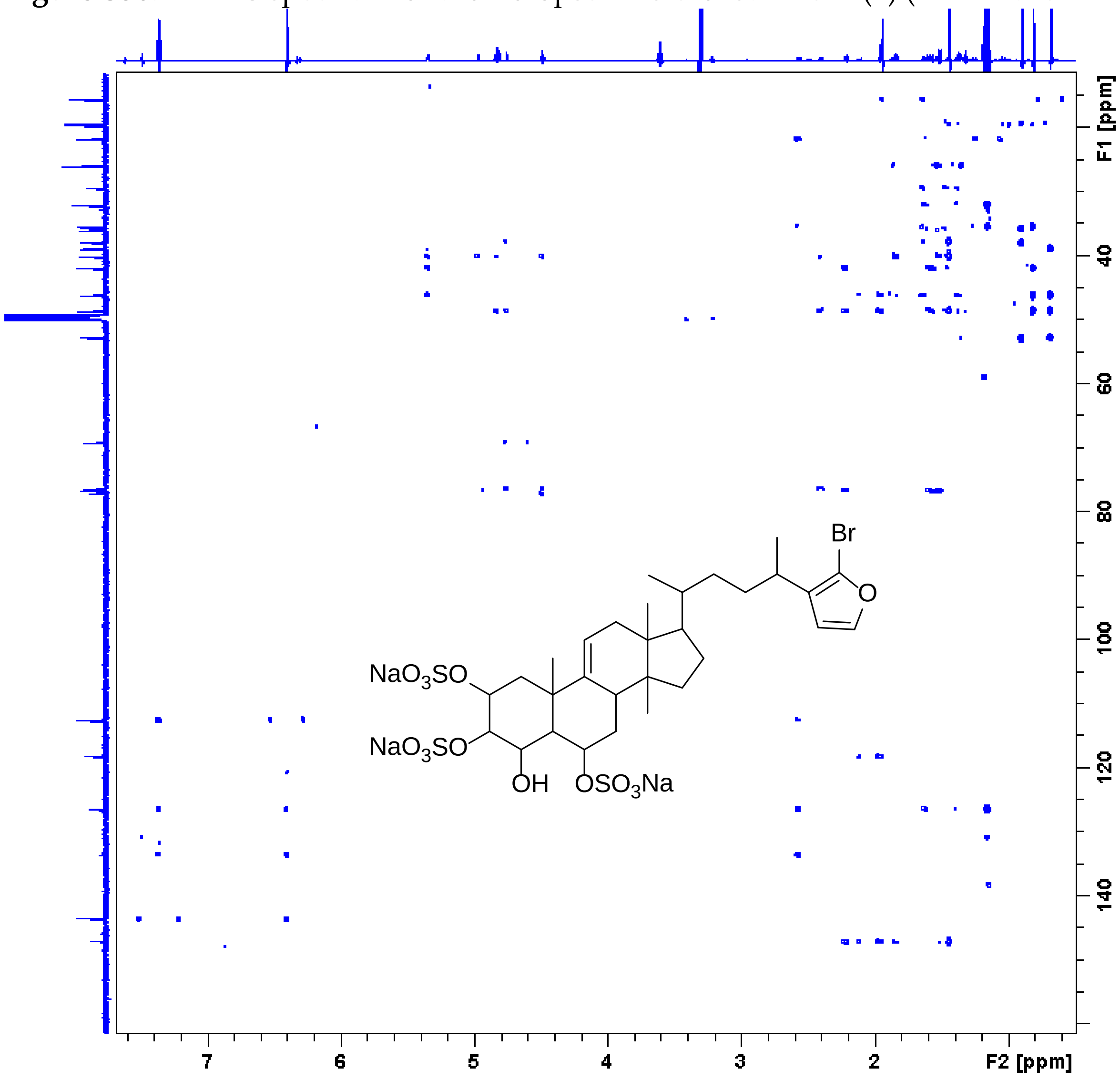

Figure S31. NOESY spectrum of bromotopsentiasterol sulfate D (**4**) (in mixture with **5** and **6**) in CD_3OD.

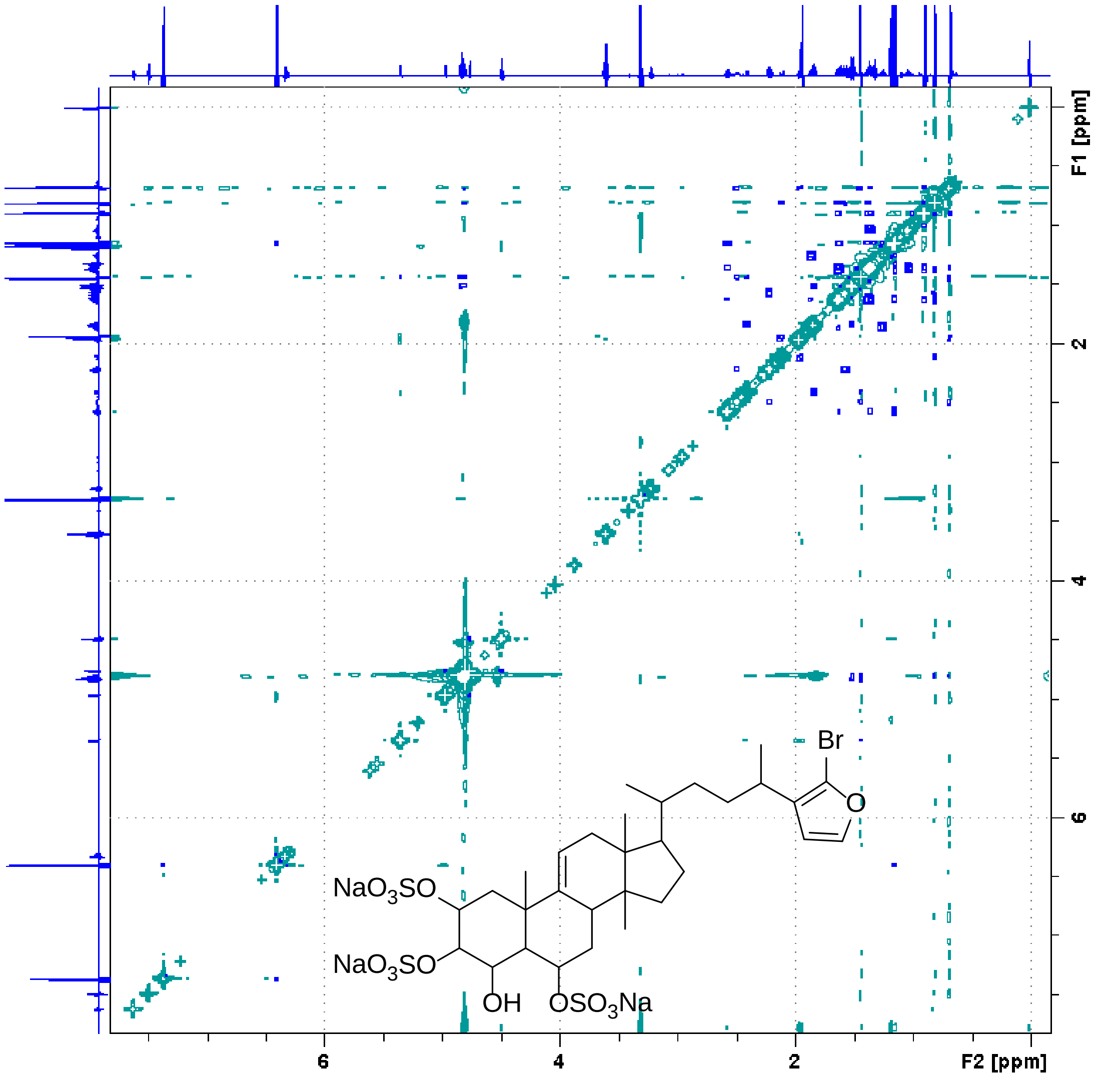

Figure S32. HRESIMS of dichlorotopsentiasterol sulfate D (**8**) (in mixture with **9**) in CD_3OD.

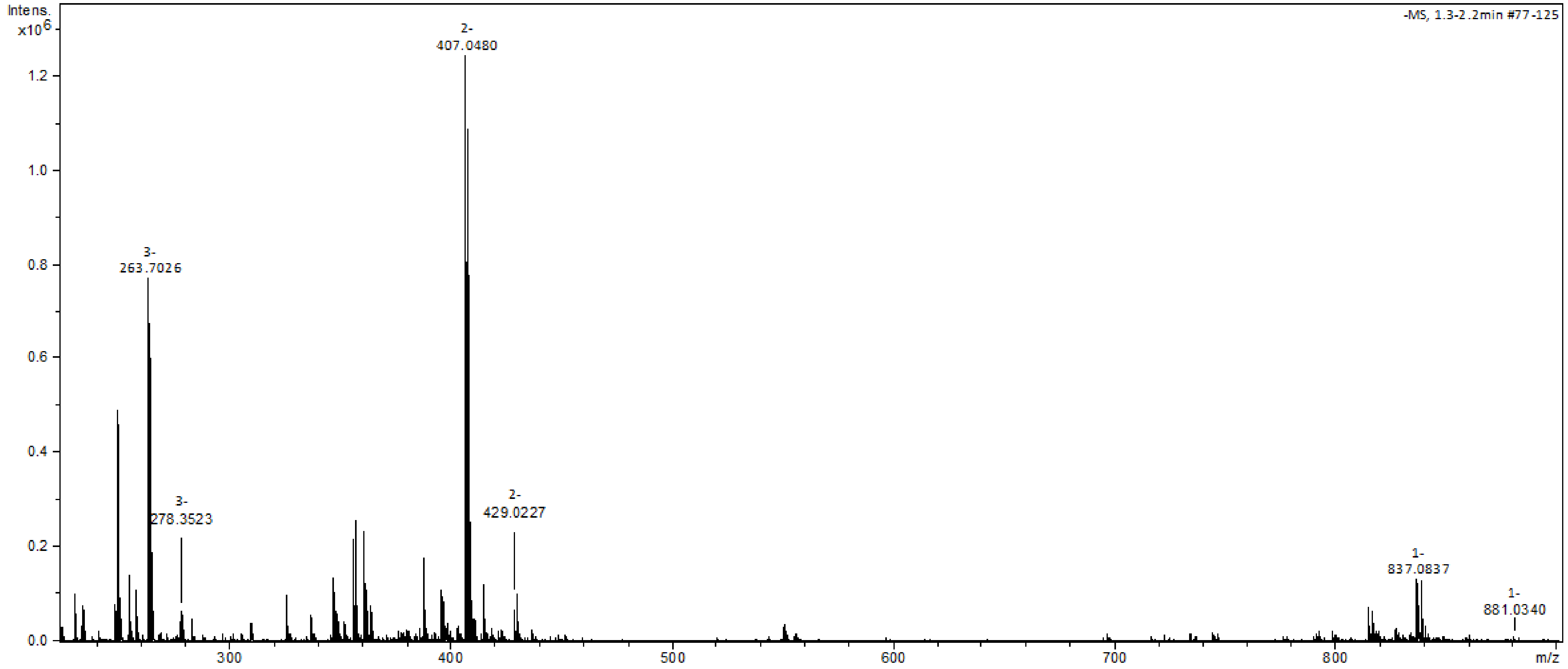

Figure S33. ^{1}H-NMR spectrum of dichlorotopsentiasterol sulfate D (**8**) (in mixture with **9**) in CD_3OD.

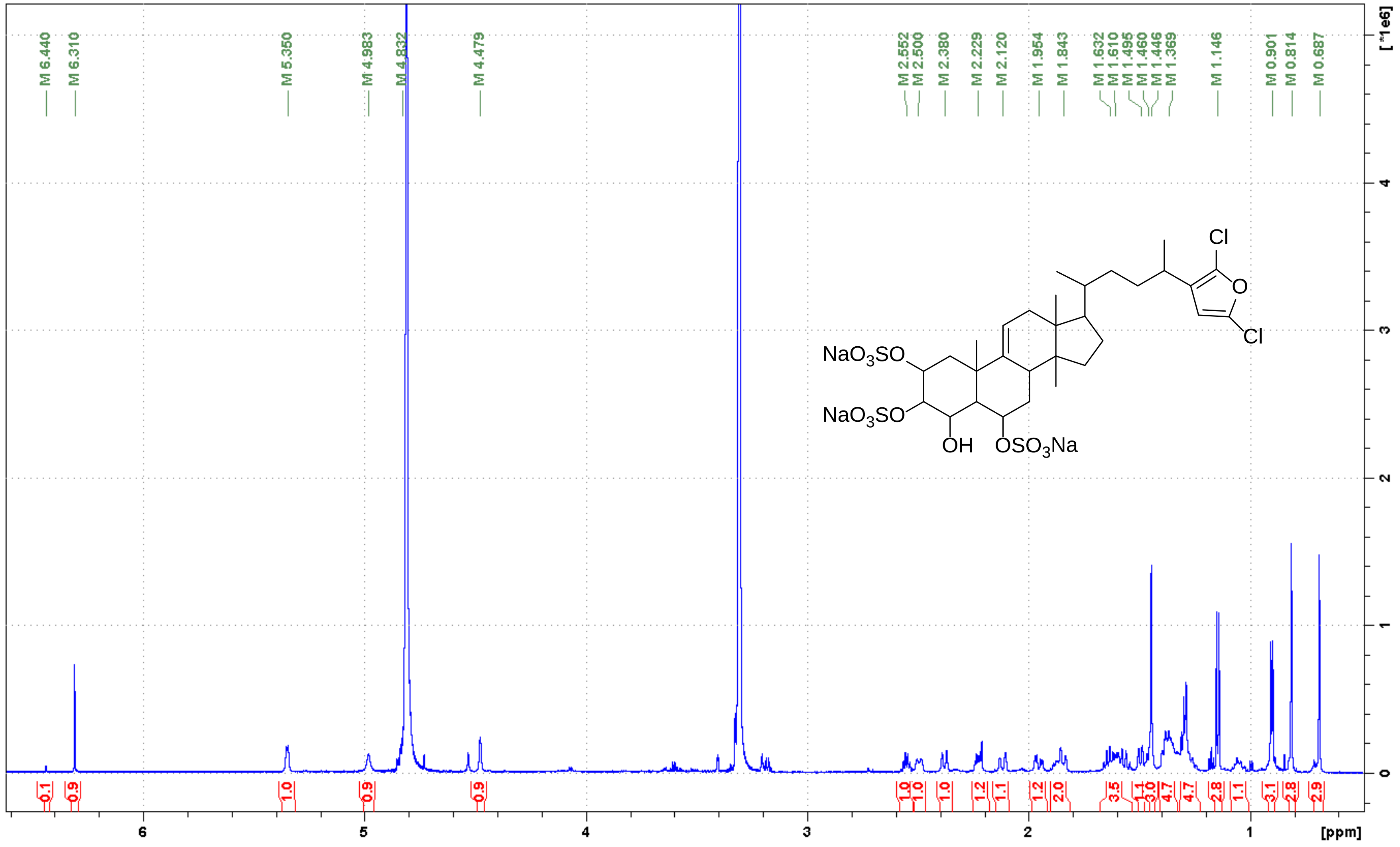

Figure S34. ^{13}C-NMR spectrum of dichlorotopsentiasterol sulfate D (**8**) (in mixture with **9**) in CD_3OD.

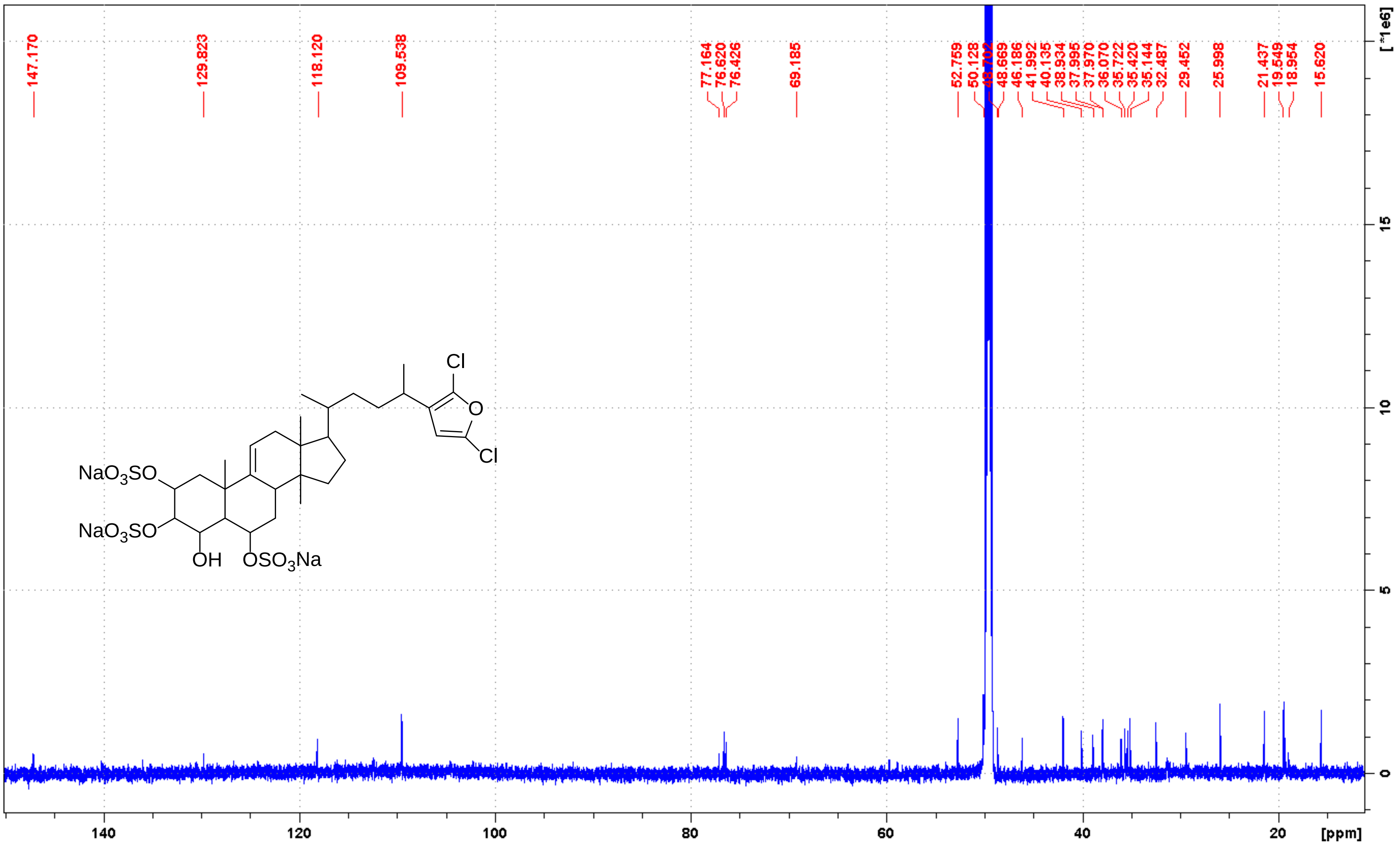

Figure S35. ^{1}H-^{1}H-COSY spectrum of dichlorotopsentiasterol sulfate D (**8**) (in mixture with **9**) in CD_3OD.

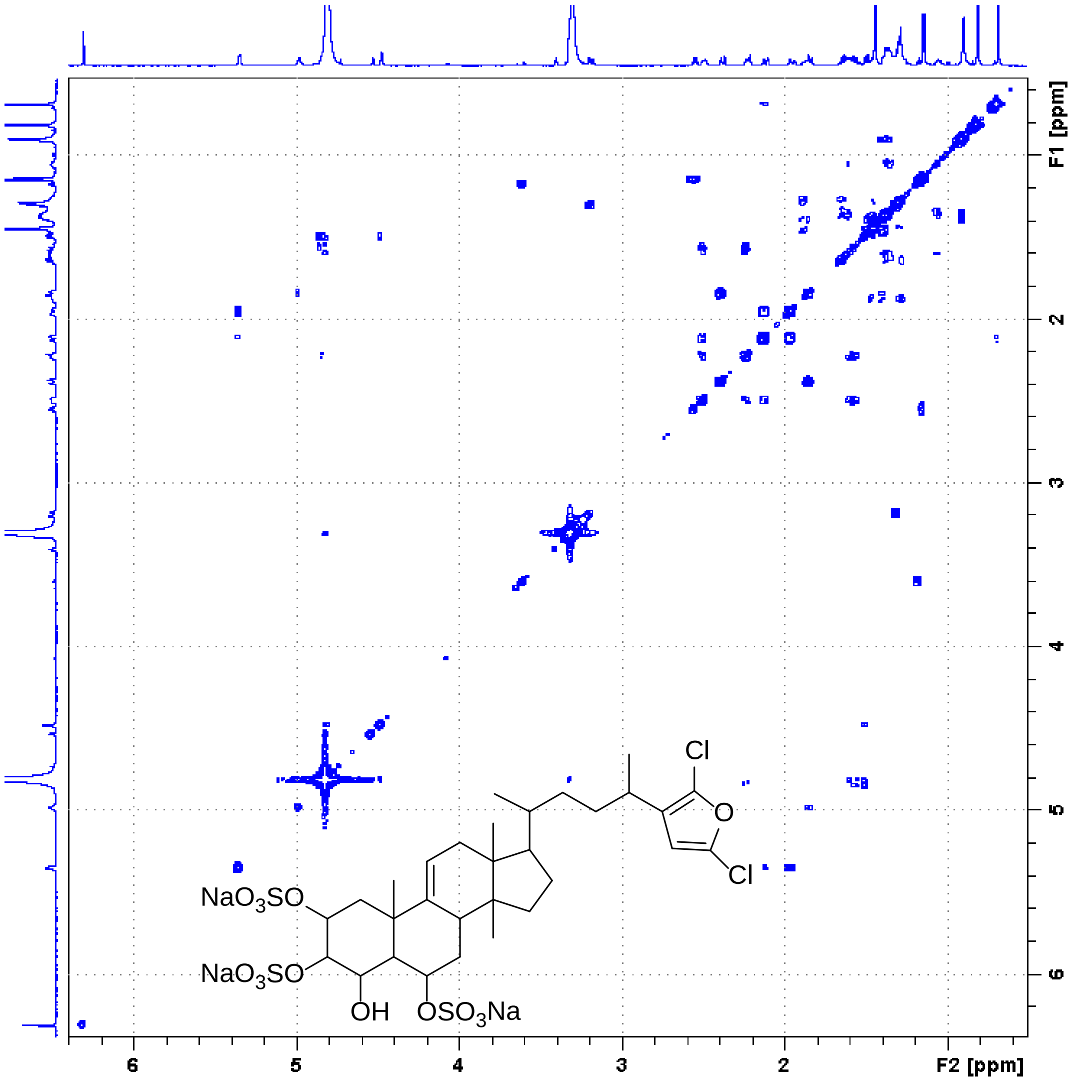

Figure S36. HSQC spectrum of dichlorotopsentiasterol sulfate D (**8**) (in mixture with **9**) in CD_3OD.

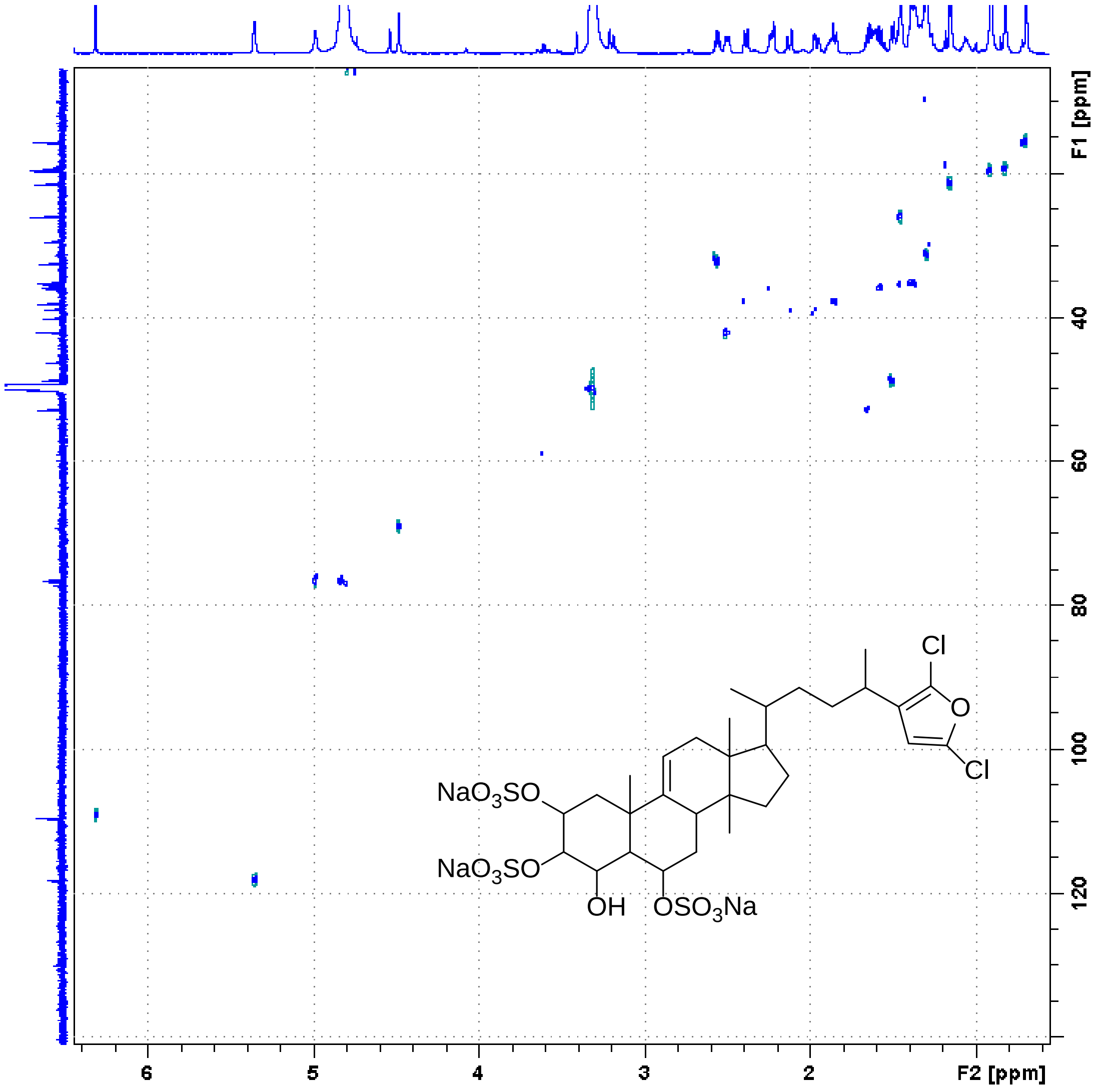

Figure S37. HMBC spectrum of dichlorotopsentiasterol sulfate D (**8**) (in mixture with **9**) in CD_3OD.

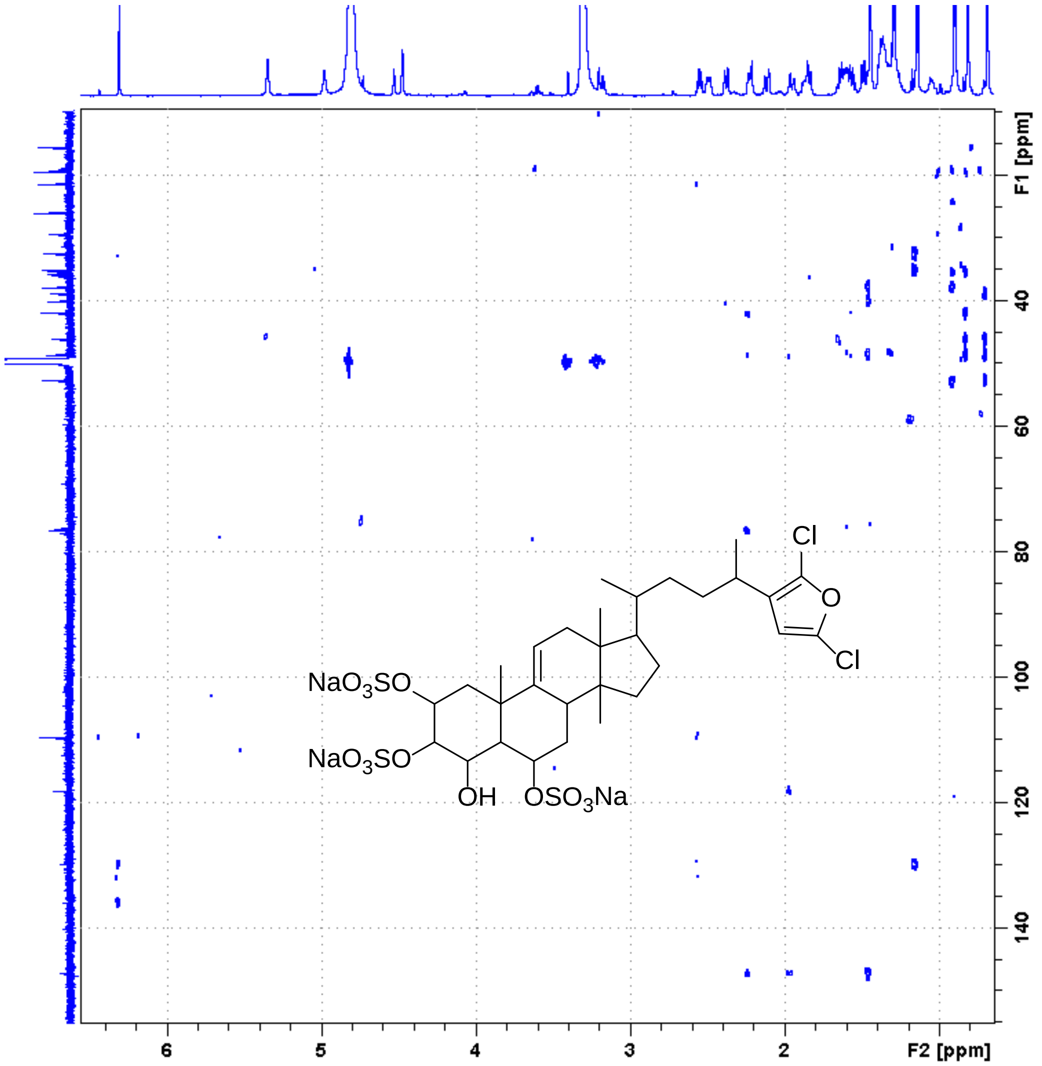

Figure S37a. Fragments of COSY spectra: **A**: for bromotopsentiasterol sulfate D (**4**) (in mixture with **5** and **6**) in CD_3OD; **B**: for a mixture of dichlorotopsentiasterol sulfate D and bromochlorotopsentiasterol sulfate D (**8**+**9**) in CD_3OD.

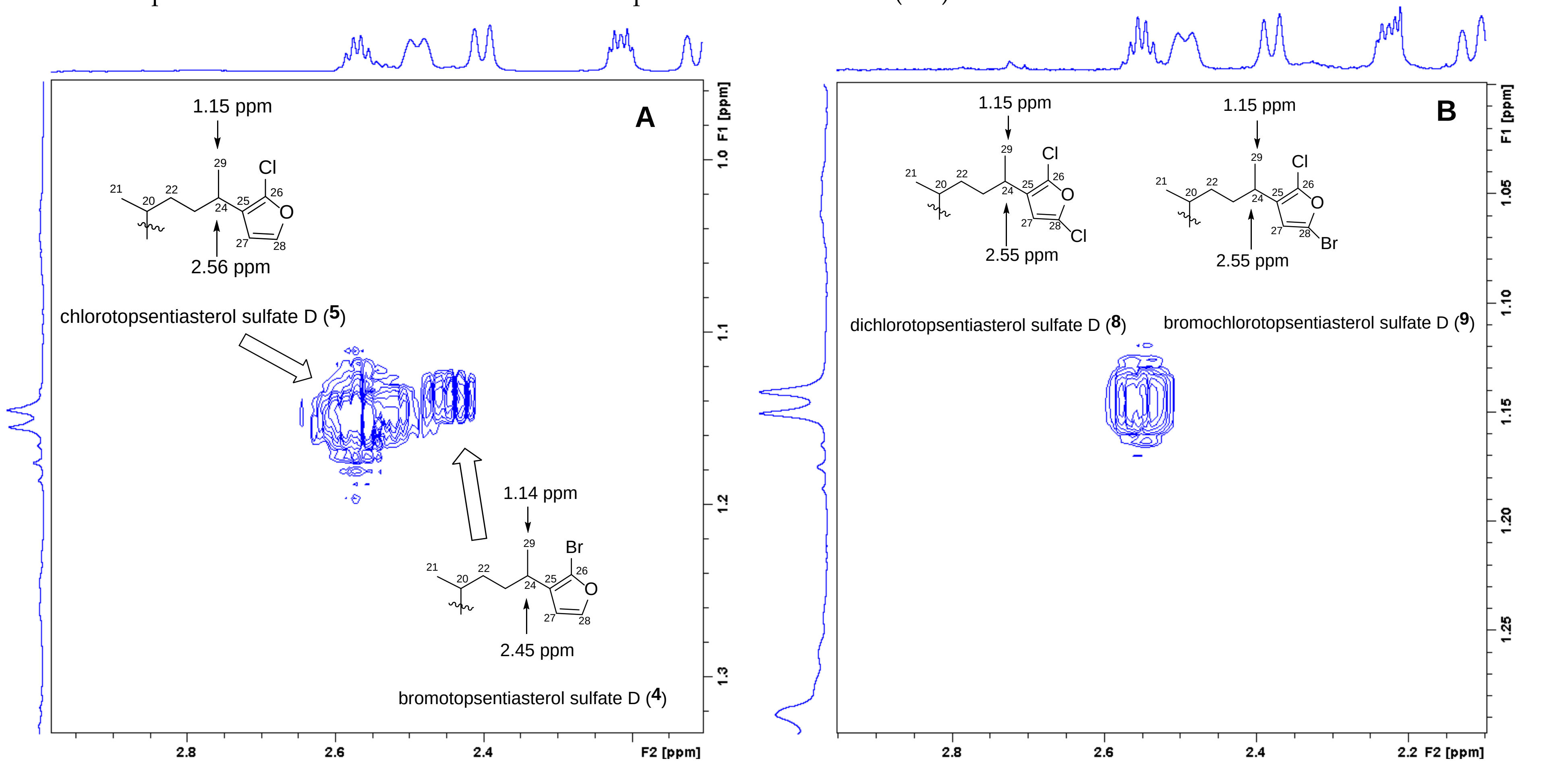

Figure S38. HRESIMS of 4β-hydroxyhalistanol sulfate C (**10**).

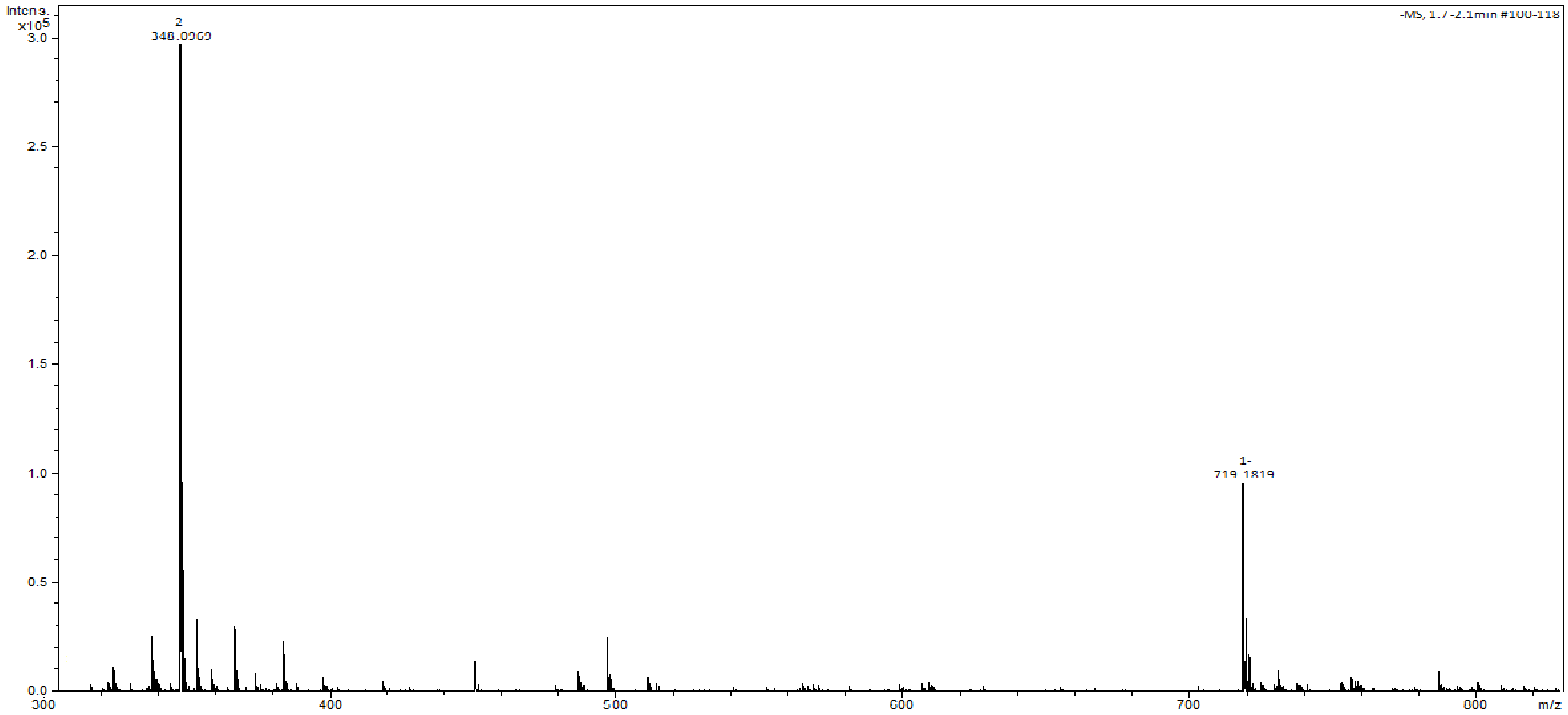

Figure S39. ^{1}H-NMR spectrum of 4β-hydroxyhalistanol sulfate C (**10**) in CD_3OD.

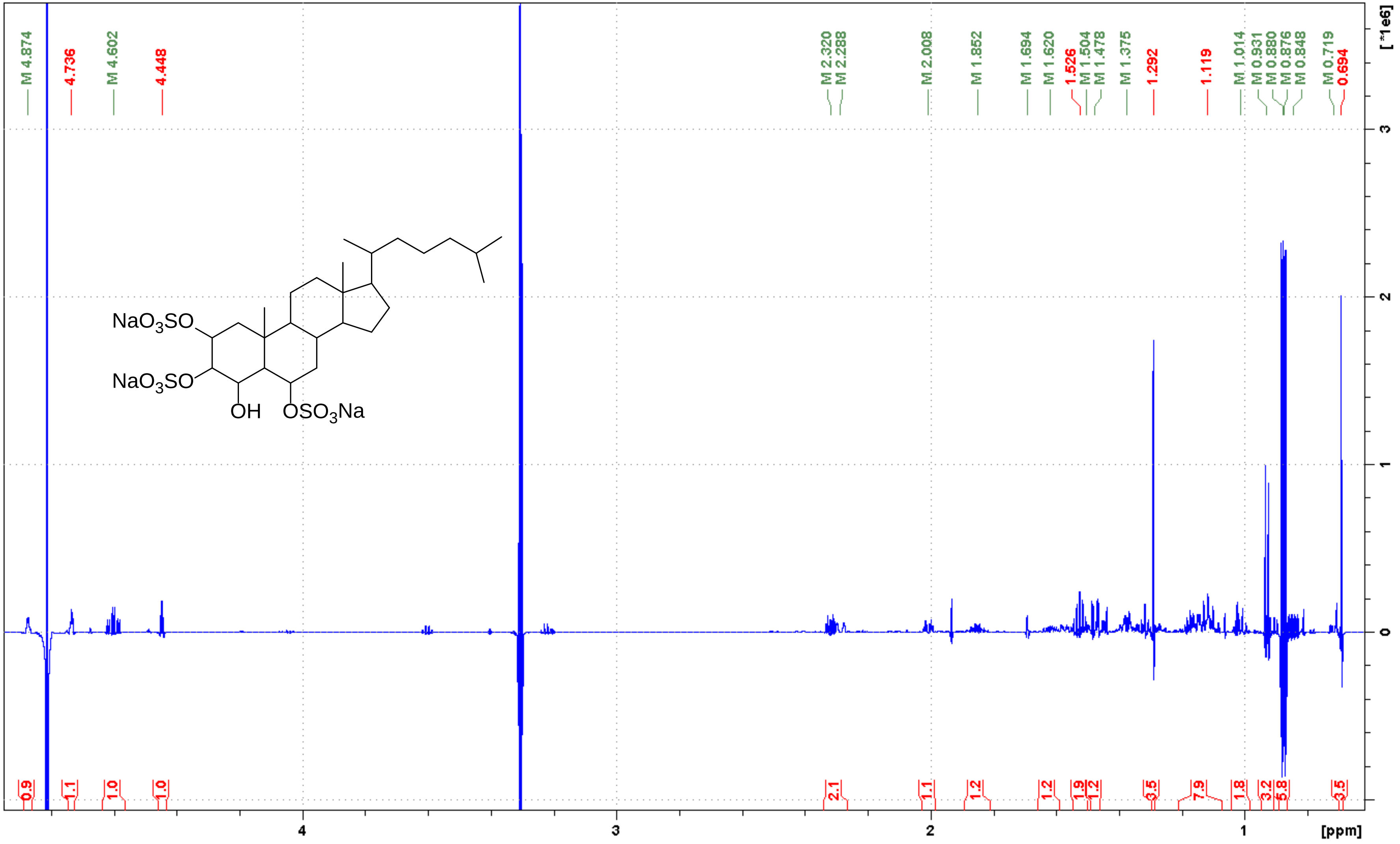

Figure S40. ^{13}C-NMR spectrum of 4β-hydroxyhalistanol sulfate C (**10**) in CD_3OD.

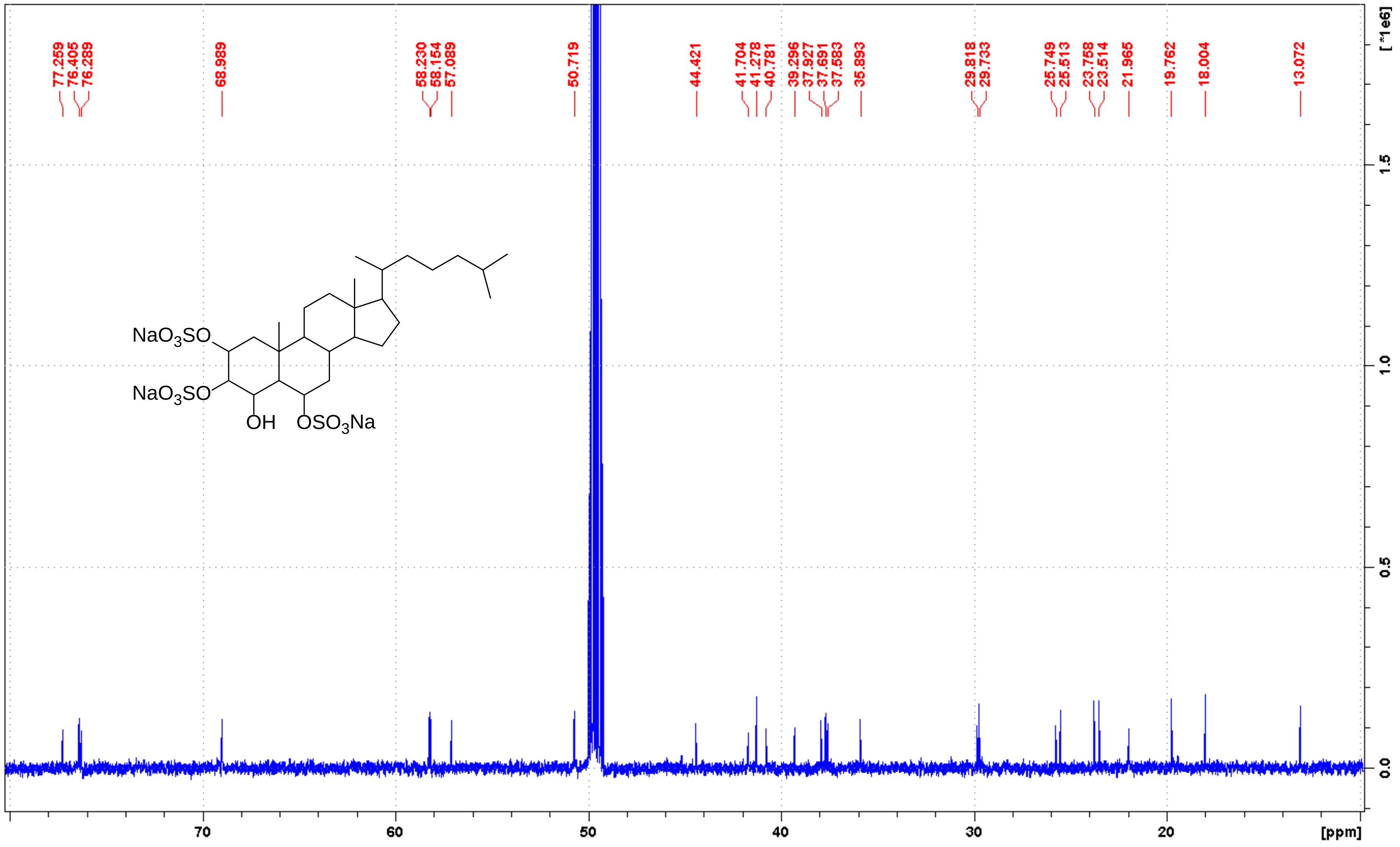

Figure S41. ^{1}H-^{1}H-COSY spectrum of 4β-hydroxyhalistanol sulfate C (**10**) in CD_3OD.

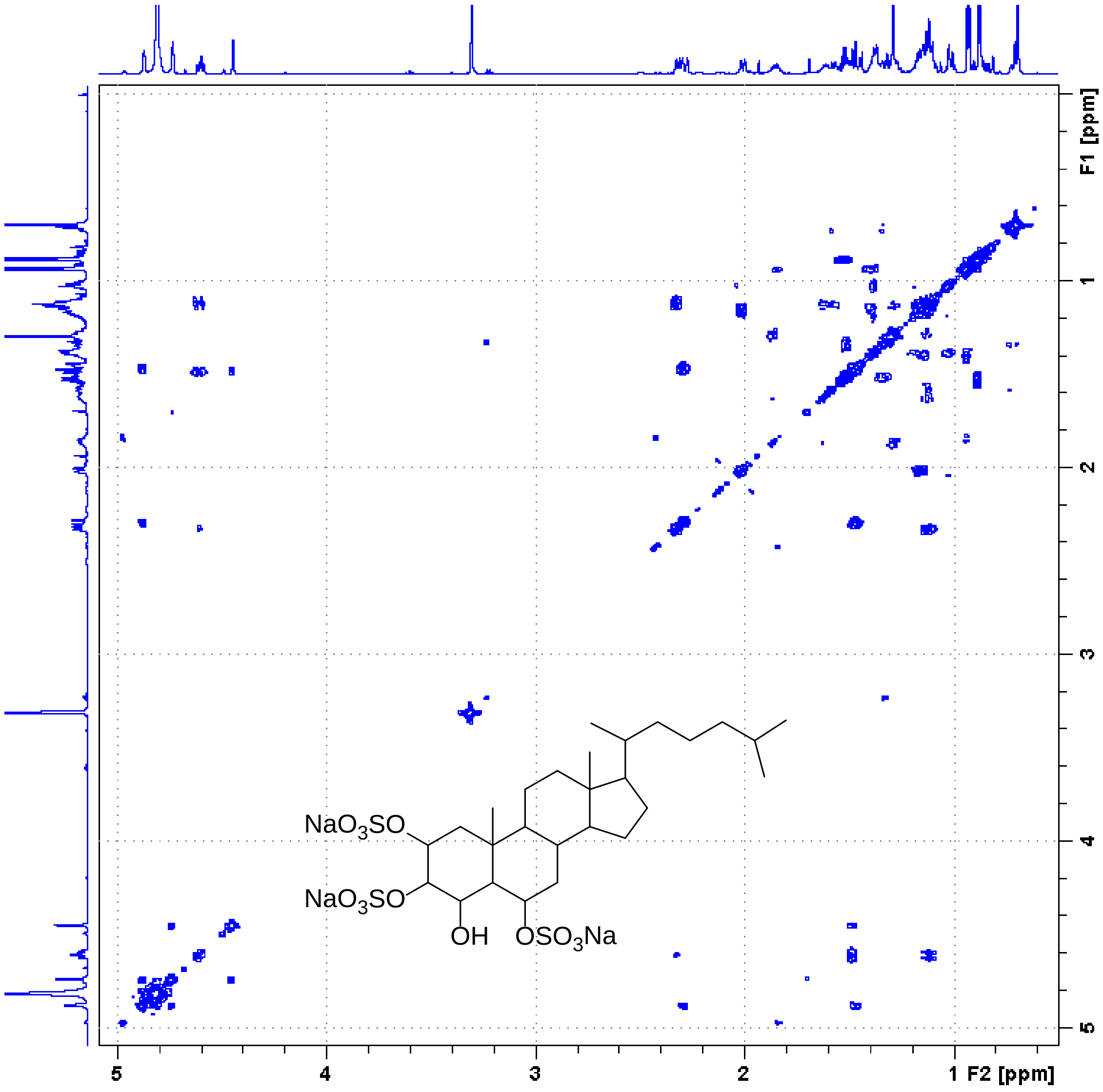

Figure S42. HSQC spectrum of 4β-hydroxyhalistanol sulfate C (**10**) in CD3OD.

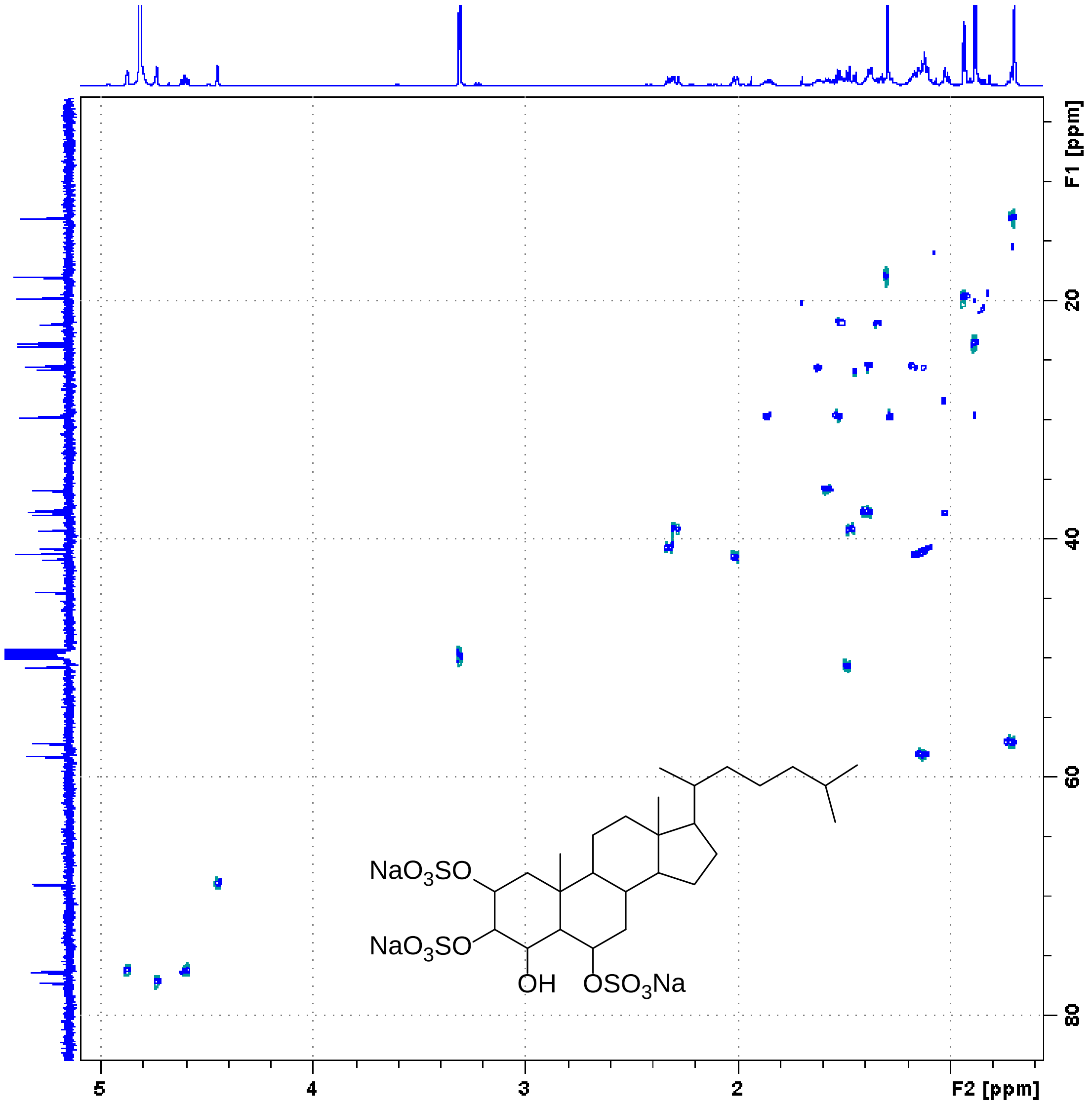

Figure S43. HMBC spectrum of 4β-hydroxyhalistanol sulfate C (**10**) in CD3OD.

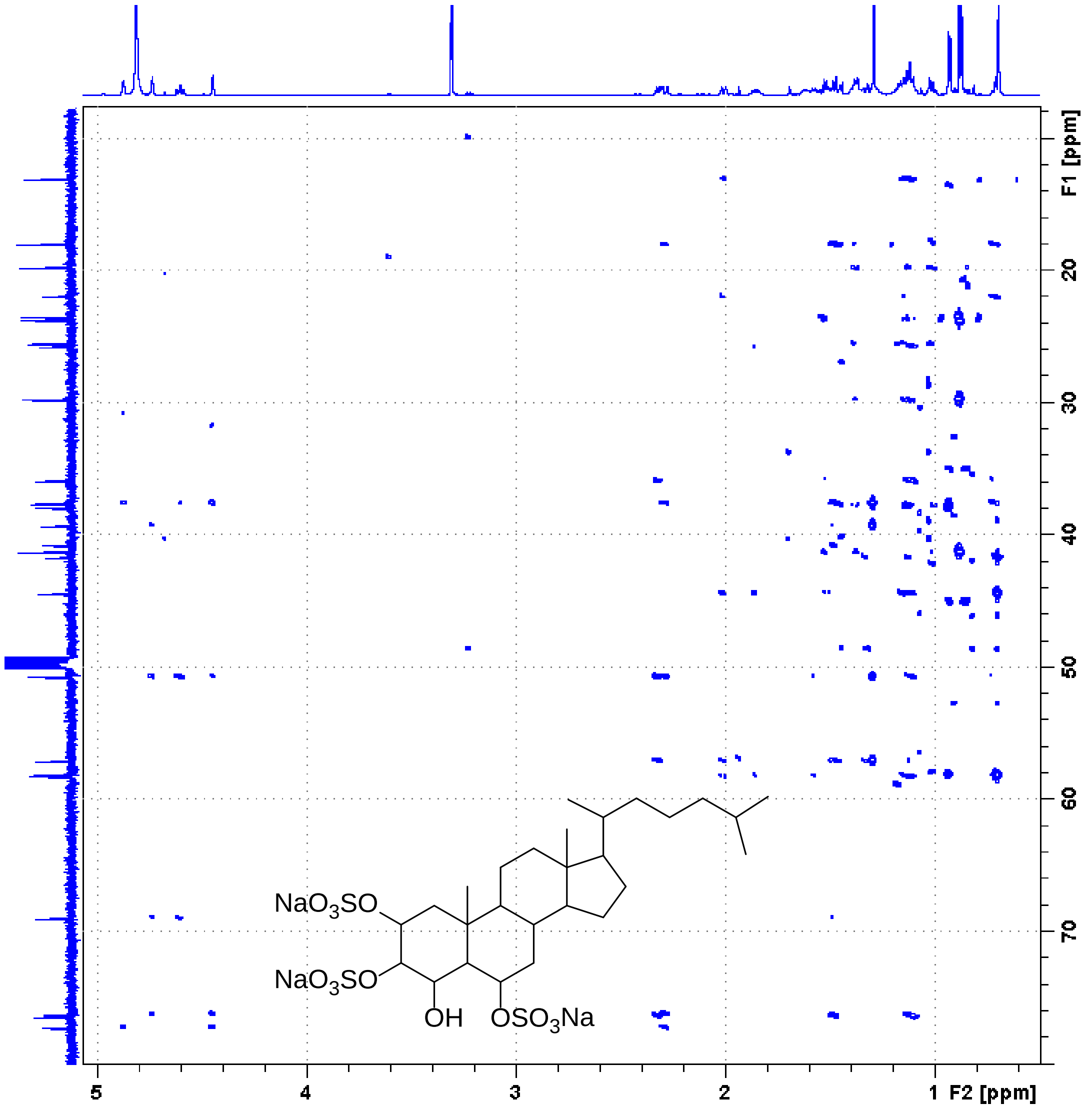

Figure S44. NOESY spectrum of 4β-hydroxyhalistanol sulfate C (**10**) in CD3OD.

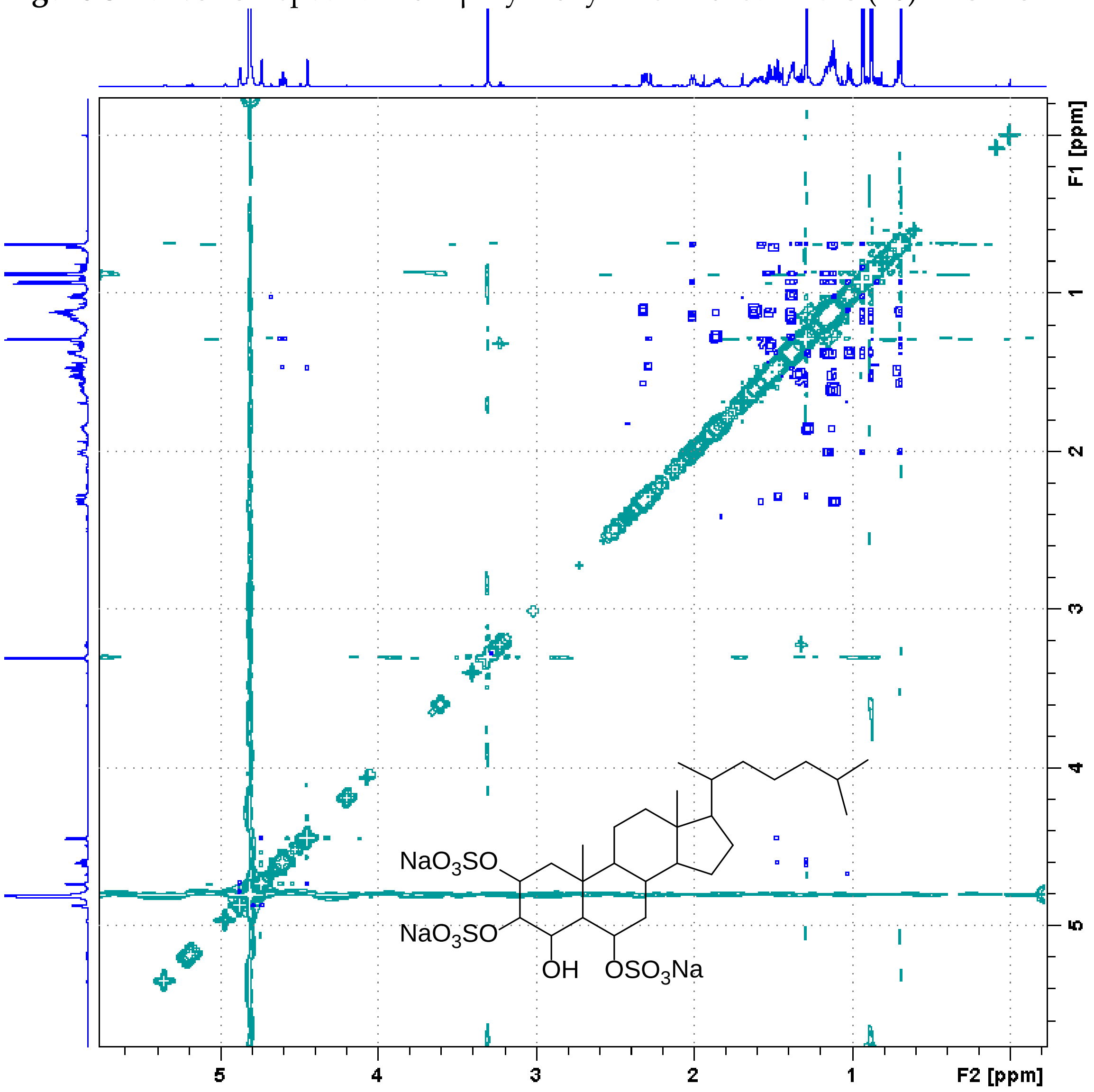

Figure S45. Structure of codisterol (**12**).

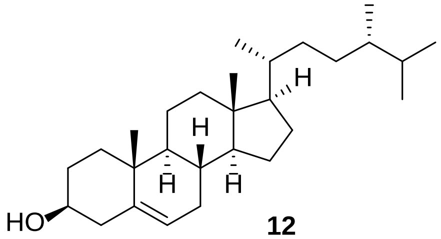

12

Figure S46. Photo of the studied sample of sponge *Halichondria vansoesti* (registration number № 049-232).

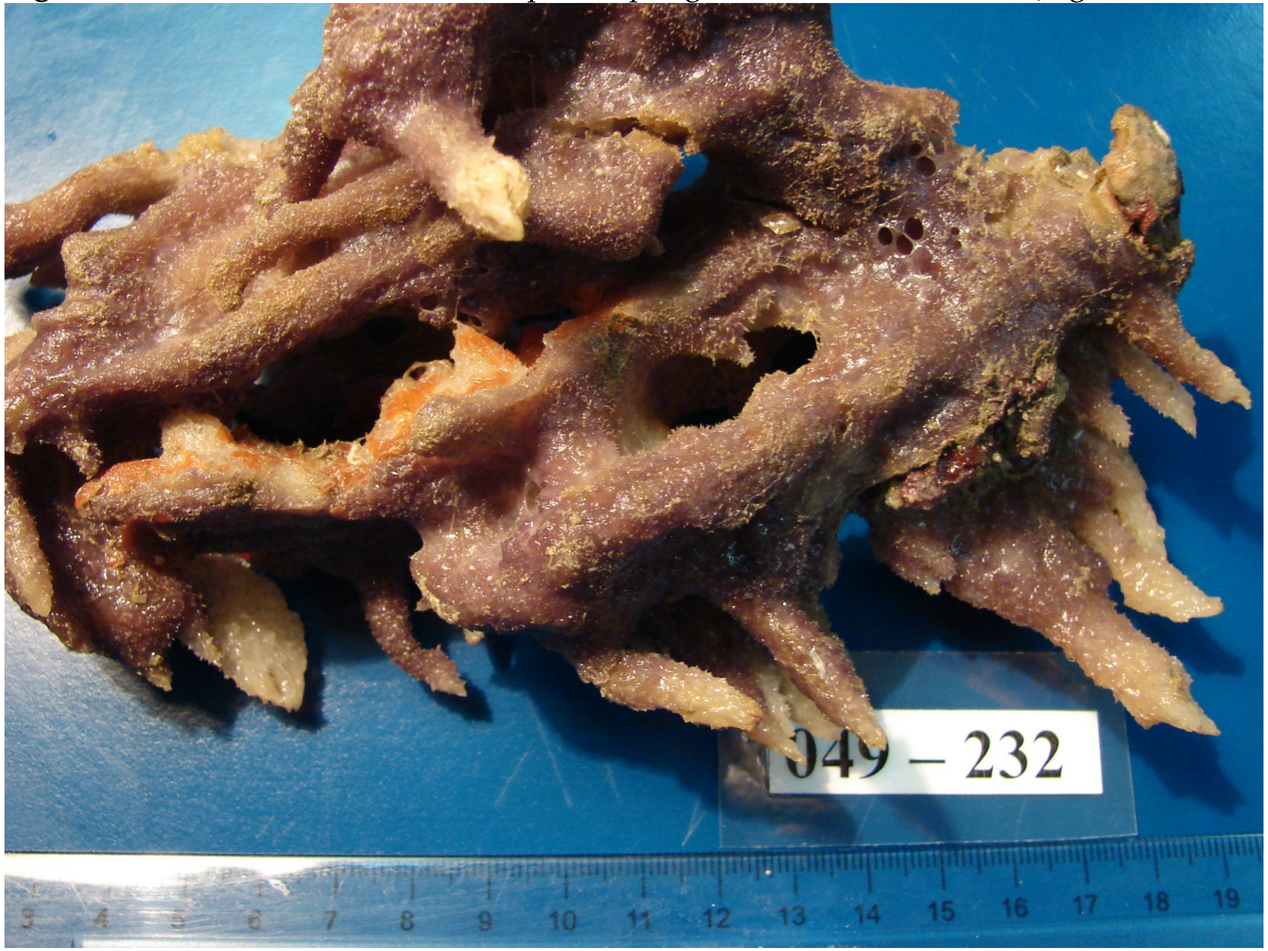

Supplement: Supplementary file 1 [file marinedrugs-17-00445-s001.pdf]
